# Notch Activity Modulates the Responsiveness of Neural Progenitors to Sonic Hedgehog Signaling

**DOI:** 10.1016/j.devcel.2015.03.005

**Published:** 2015-05-26

**Authors:** Jennifer H. Kong, Linlin Yang, Eric Dessaud, Katherine Chuang, Destaye M. Moore, Rajat Rohatgi, James Briscoe, Bennett G. Novitch

**Affiliations:** 1 Department of Neurobiology, David Geffen School of Medicine at UCLA, Los Angeles, CA 90095, USA; 2 Eli and Edythe Broad Center of Regenerative Medicine and Stem Cell Research, David Geffen School of Medicine at UCLA, Los Angeles, CA 90095, USA; 3 The Francis Crick Institute, Mill Hill Laboratory, London NW7 1AA, UK; 4 Department of Medicine, Stanford University School of Medicine, Stanford, CA 94305, USA; 5 Department of Biochemistry, Stanford University School of Medicine, Stanford, CA 94305, USA

## Abstract

Throughout the developing nervous system, neural stem and progenitor cells give rise to diverse classes of neurons and glia in a spatially and temporally coordinated manner. In the ventral spinal cord, much of this diversity emerges through the morphogen actions of Sonic hedgehog (Shh). Interpretation of the Shh gradient depends on both the amount of ligand and duration of exposure, but the mechanisms permitting prolonged responses to Shh are not well understood. We demonstrate that Notch signaling plays an essential role in this process, enabling neural progenitors to attain sufficiently high levels of Shh pathway activity needed to direct the ventral-most cell fates. Notch activity regulates subcellular localization of the Shh receptor Patched1, gating the translocation of the key effector Smoothened to primary cilia and its downstream signaling activities. These data reveal an unexpected role for Notch shaping the interpretation of the Shh morphogen gradient and influencing cell fate determination.

## Highlights

• Changes in Notch signaling alter the dorsoventral identity of neural progenitors
• Activation and inactivation of Notch signaling alter cellular responses to Shh
• Notch activity is required for efficient trafficking of Smo to primary cilia
• Notch activity regulates the subcellular distribution of the Shh receptor Ptch1

## Graphical Abstract

**Figure:**
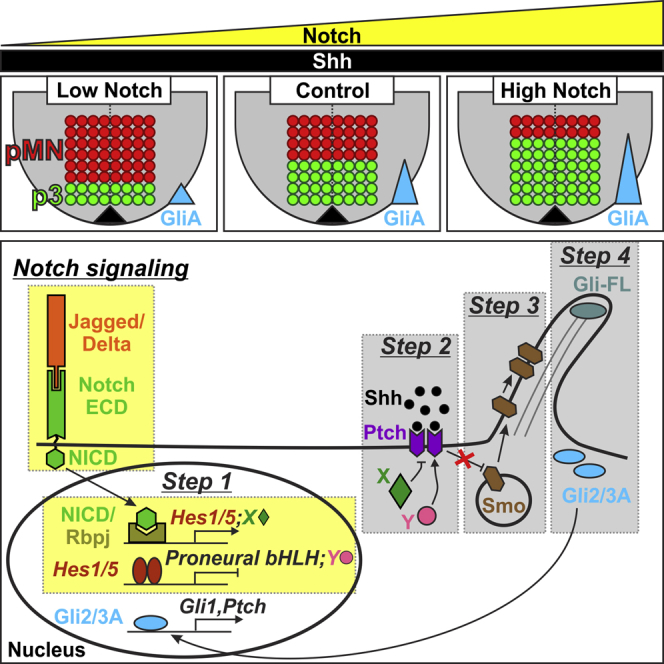

## Teaser

Cell fate assignment in the ventral spinal cord depends on the ability of neural progenitors to interpret the morphogen Shh. Kong and Yang et al. show that Notch signaling tunes neural progenitor responses to Shh by regulating trafficking of the Shh receptor Patched1 and downstream effector Smoothened to primary cilia.

## Introduction

Neuronal and glial diversity in the CNS emerges in large part through the concomitant and combinatorial actions of morphogen signals such as Sonic hedgehog (Shh), Bone Morphogenetic Proteins (BMPs), Wnts, and retinoids that organize neural progenitor cells (NPCs) into discrete domains along the dorsoventral and rostrocaudal axes (Briscoe and Novitch, 2008; Le Dréau and Martí, 2013; Butler and Bronner, 2015). Each of these domains is defined by its expression of unique combinations of transcription factors and ability to generate specific classes of neurons and glia (Briscoe and Novitch, 2008; Rowitch and Kriegstein, 2010; Le Dréau and Martí, 2013; Butler and Bronner, 2015). The prevailing model for morphogen signaling posits that differential cellular responses arise due to the signal concentrations that cells encounter (Rogers and Schier, 2011), yet the duration of exposure to a fixed amount of signal can also elicit graded domain responses and influence fate decisions (Kutejova et al., 2009). These results suggest that an important aspect of morphogen interpretation is the ability of cells to maintain their responsiveness to these cues as development proceeds. However, the mechanisms that permit this competence over time are not well understood.

One of the best studied examples of morphogen signaling is the patterning response of NPCs in the ventral spinal cord to Shh. Shh acts on NPCs in a dose-dependent manner, binding to its primary receptors Patched1 and 2 (Ptch1/2) to initiate a cascade of intracellular signaling events centered on the translocation of the G-protein-coupled receptor Smoothened (Smo) to primary cilia (Eggenschwiler and Anderson, 2007; Dessaud et al., 2008; Ribes and Briscoe, 2009). The presence of Smo in cilia modulates the proteolysis and activity of the Gli family of Zn-finger transcription factors, which in turn regulate the expression of many NPC fate determinants that subdivide the ventral spinal cord into three distinct ventral NPC domains: p3, pMN, and p2 (Briscoe and Novitch, 2008; Dessaud et al., 2008; Ribes and Briscoe, 2009). These domains are distinguished by their shared expression of the transcription factor Nkx6.1 and differential expression of Nkx2.2, Olig2, and Irx3, respectively (Mizuguchi et al., 2001; Novitch et al., 2001; Briscoe and Novitch, 2008; Dessaud et al., 2008). The pMN gives rise to motor neurons (MNs), while the p3 and p2 domains produce distinct classes of spinal interneurons that modulate MN activities. Later in development, Olig2^+^ NPCs form a domain of oligodendrocyte precursors (pOLs) that disperse and migrate throughout the spinal cord before differentiating into myelinating oligodendrocytes (Rowitch and Kriegstein, 2010). The p3 and p2 domains similarly transform into astroglial progenitor groups (pVA3 and pVA2), producing astrocytes that colonize distinct regions of the ventral spinal cord (Muroyama et al., 2005; Hochstim et al., 2008).

While these fates can be specified through the administration of different concentrations of Shh ligand in vitro (Dessaud et al., 2008; Ribes and Briscoe, 2009), NPCs also acquire their ventral identities through time-dependent mechanisms. NPCs treated with moderate doses of Shh initially express the pMN determinant Olig2; however, if Shh/Gli signaling is sustained, they subsequently express Nkx2.2 and adopt the more ventral p3 fate (Dessaud et al., 2007, 2010; Balaskas et al., 2012). Recent studies in the zebrafish spinal cord have further demonstrated that progenitor maintenance mediated by the Notch signaling pathway plays an important role enabling later born Shh-induced cell types to emerge (Huang et al., 2012). Together, these findings indicate that cells must remain in an undifferentiated state to properly interpret the Shh morphogen gradient, but do not resolve the mechanism by which the maintenance of NPC characteristics influences Shh responsiveness and whether retaining cells in a progenitor state influences spatial patterning.

The Notch signaling pathway serves as a major regulator of NPC maintenance and both neuronal and glial development (Gaiano and Fishell, 2002; Pierfelice et al., 2011). Notch receptors are broadly expressed by NPCs and are activated by the Delta-like and Jagged families of transmembrane ligands presented by neighboring cells (Kageyama et al., 2009; Pierfelice et al., 2011). Activated Notch receptors are cleaved by the Presenilin γ-secretase complex, liberating Notch intracellular domain (NICD) fragments. NICD subsequently forms transcriptional activating complexes with the DNA binding protein Rbpj and members of the mastermind-like (MAML) coactivator family (Kageyama et al., 2009; Pierfelice et al., 2011). Rbpj-NICD-MAML complexes regulate a number of targets most notably Hes genes, bHLH transcription factors that repress proneural genes, inhibit neuronal differentiation, and promote NPC maintenance (Kageyama et al., 2007, 2009; Pierfelice et al., 2011). Through these actions, Notch signaling suppresses neuronal differentiation and endows cells with gliogenic potential. NICD misexpression can further promote specific glial cell fates, such as radial glia in the forebrain, Müller glia in the retina, and astrocytes in neural stem cell cultures (Furukawa et al., 2000; Gaiano et al., 2000; Scheer et al., 2001; Ge et al., 2002) while inhibiting oligodendrocyte differentiation (Wang et al., 1998). These data implicate a role for Notch in glial fate selection, although the mechanisms underlying these effects remain unclear.

Here, we test the contributions of Notch signaling on both the establishment of NPC identities and glial fate determination. We show that activation and inactivation of the Notch pathway modify the responses of NPCs to Shh, altering both their dorsoventral register and ability to generate distinct classes of neurons and glial cells. Notch activity strikingly acts at the most proximal steps in the Shh transduction pathway, affecting the trafficking of Smo and Ptch1 to primary cilia. Together, these findings reveal a role for Notch signaling shaping the interpretation of the Shh morphogen gradient and assignment of cell fates.

## Results

### Manipulation of Notch Signaling Alters the Dorsoventral Register of NPCs

**Figure 1:**
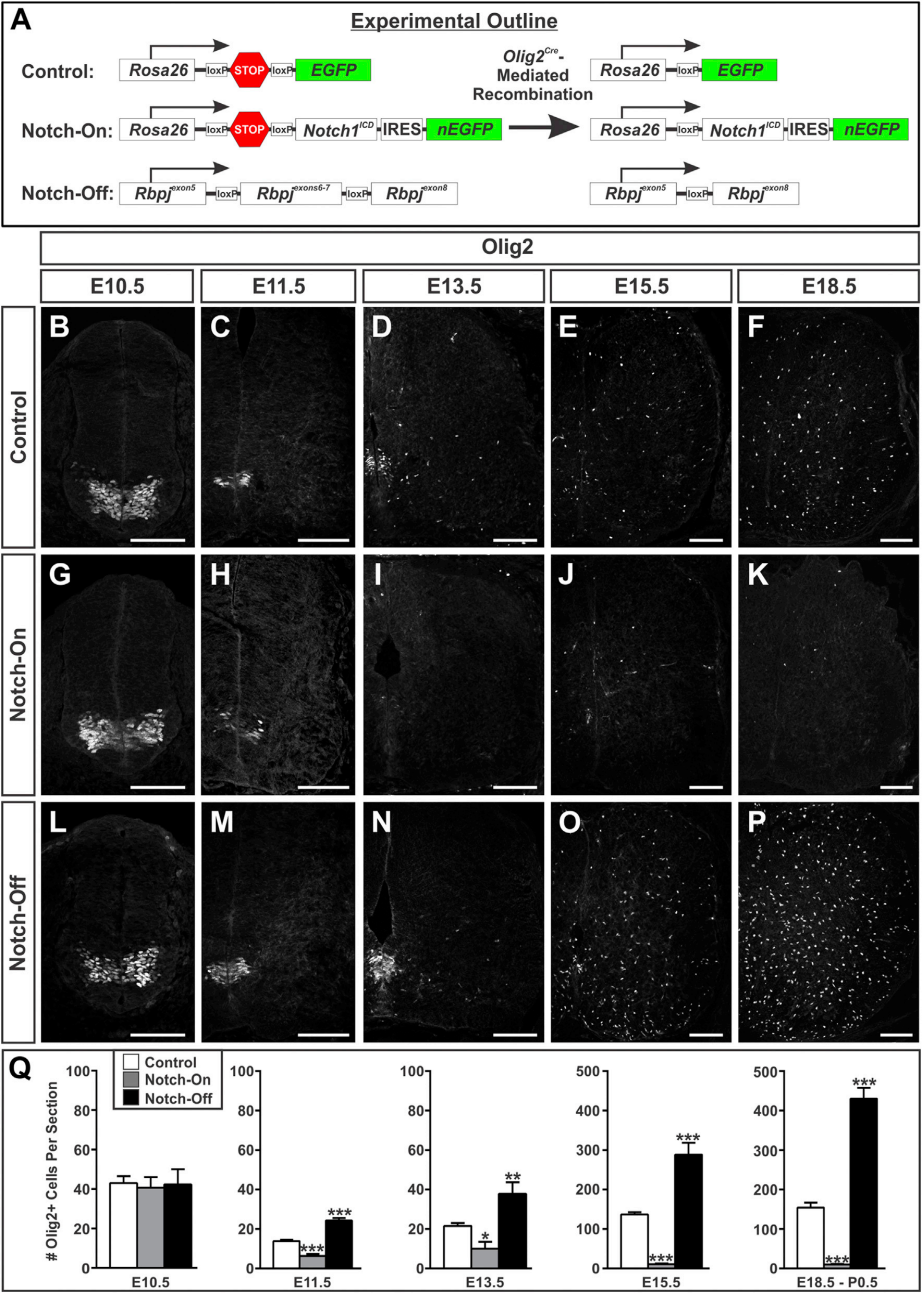
Manipulation of Notch Signaling Alters Olig2 Expression (A) Schematic of *Olig2*^*Cre*^-mediated manipulations used to activate or inactivate Notch signaling. Notch-On indicates NICD misexpression and Notch-Off indicates *Rbpj* deletion. Control conditions include crosses to mice carrying a *R26R*^*GFP*^ reporter. (B–F) At E10.5–E11.5, Olig2 is initially expressed by MN progenitors and later oligodendrocyte progenitors. (G–P) In Notch-On mice, Olig2^+^ cells decline from E11.5 onward. In Notch-Off mice, Olig2^+^ cells increase. Scale bars represent 100 μm. (Q) Quantification of Olig2^+^ cells per spinal cord half at the indicated time points. Plots show the mean ± SEM from multiple sections collected from 4–25 embryos from each group. ^∗^p < 0.05, ^∗∗^p < 0.01, ^∗∗∗^p < 0.001. See also Figures S1 and S2.

We first used *Olig2*^*Cre*^ mice (Dessaud et al., 2007) to selectively activate or inactive Notch signaling in the p3 and pMN domains between embryonic days (E) 9.5 and 10.5 (Figures S1A–S1W). This strategy was accomplished by crossing *Olig2*^*Cre*^ to mice harboring (1) a Cre-inducible *R26R*^*GFP*^ transgenic reporter (Mao et al., 2001) (control condition), (2) a *R26R*^*NICD-GFP*^ transgene and reporter (Murtaugh et al., 2003) (“Notch-On” condition), or (3) a Cre-inactivatable *Rbpj* allele (Han et al., 2002), along with the *R26R*^*GFP*^ transgenic reporter (“Notch-Off” condition) (Figures 1A). The impact of these Notch pathway manipulations was evident by E11.5, as Notch-On mice displayed elevated expression of the Notch effectors Hes1 and *Hes5,* which are normally very low in the pMN and reduced expression of proneural transcription factors, including Neurog2, Ascl1, and *Neurog3* (Figures S2A–S2N). Conversely, Notch-Off mice displayed reductions in Hes1 and *Hes5* expression and increased levels of Neurog2, Ascl1, and *Neurog3* (Figures S2O–S2U). While the ventral ventricular zone (VZ) narrowed in Notch-Off mice, a contiguous band of Sox2^+^ NPCs was maintained throughout development, and both the neuroepithelial architecture and apicobasal polarity of progenitors were preserved (Figures S2V–S2AI). This phenotype contrasts with mutations in other members of the Notch pathway such as *Hes1* and *Hes5* whose combined loss disrupts the neuroepithelium (Hatakeyama et al., 2004). The persistence of NPCs and neuroepithelial organization in *Olig2*^*Cre*^;Notch-Off mutants may be explained by the lasting presence of Hes1 in ventral progenitors despite the loss of *Rbpj* (Figures S2Q and S2R), most likely due to Notch-independent activation of Hes1 by Shh, as has been described in other tissues (Ingram et al., 2008; Wall et al., 2009).

**Figure 2:**
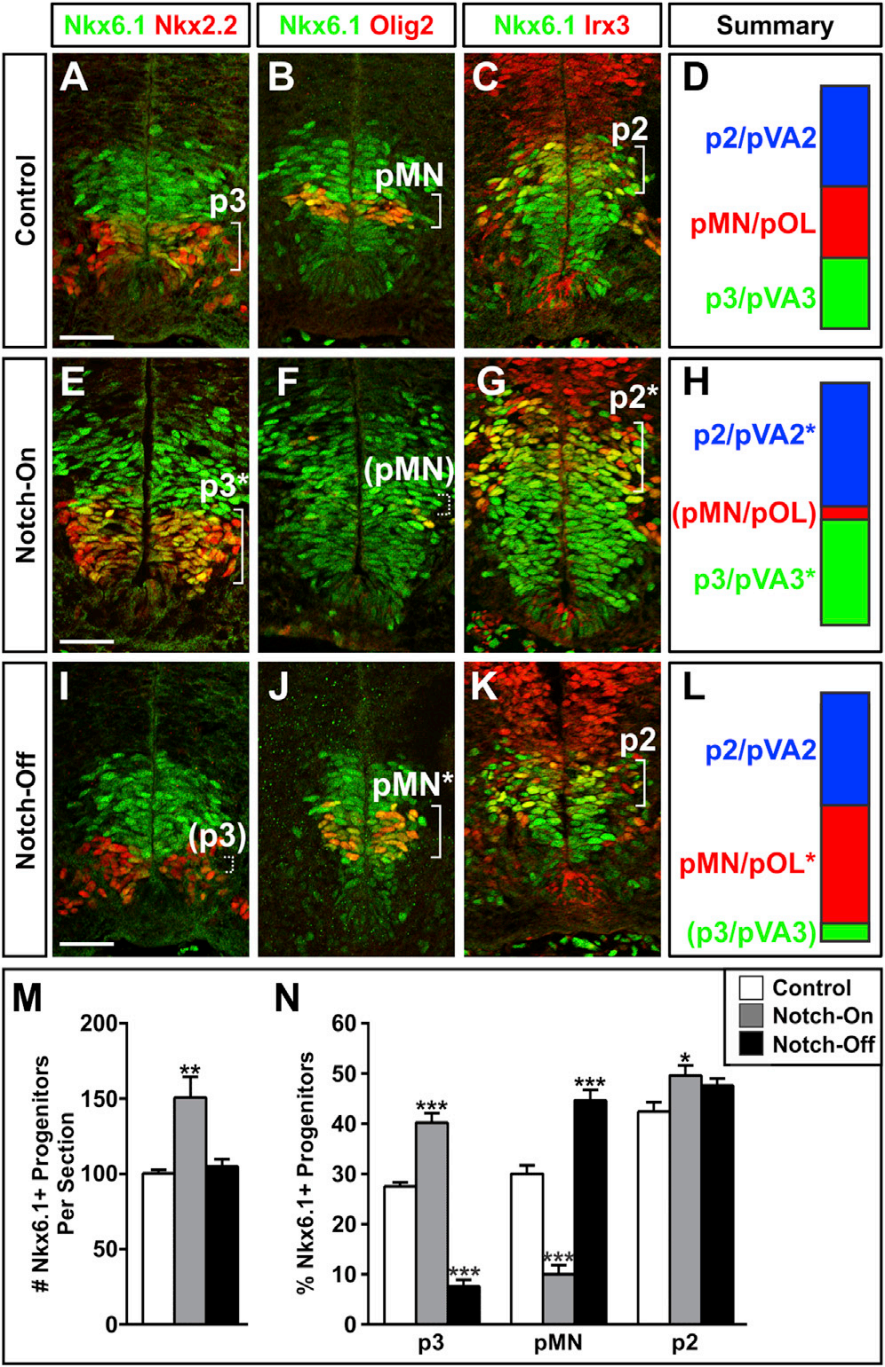
Changes in Notch Signaling Alter the Dorsoventral Identities of Ventral Spinal Cord Progenitors (A–D) In E11.5 control spinal cords, ventral progenitors are distinguishable by coexpression of Nkx6.1 and Nkx2.2 (p3), Nkx6.1 and Olig2 (pMN), and Nkx6.1 and Irx3 (p2). (E–H) More Nkx6.1^+^ progenitors are present in Notch-On mutants. Within this population, the percentage expressing Nkx2.2 increased while the percentage expressing Olig2 decreased. (I–L) Notch-Off mutants contain a reduced percentage of Nkx6.1^+^ progenitors expressing Nkx2.2 and reciprocal increase in Olig2. Scale bars represent 50 μm. (M and N) Quantification of the total number of Nkx6.1^+^ progenitors present and their subdivision into p3, pMN, and p2. Plots show the mean ± SEM from multiple sections collected from seven to nine embryos for each group. ^∗^p < 0.05, ^∗∗^p < 0.01, ^∗∗∗^p < 0.001. See also Figures S2 and S4.

We next examined the impact of these Notch manipulations on dorsoventral patterning. Remarkably, activating Notch signaling led to a notable reduction in Olig2^+^ pMN cells by ∼E11.5 and a nearly complete loss of Olig2^+^ NPCs throughout the rest of embryonic development (Figures 1B–1K and 1Q). Notch-Off mice exhibited the reciprocal phenotype, with an ∼1.5 to ∼2.5-fold increase in the number of Olig2^+^ progenitors from E11.5 to postnatal day (P) 0.5 (Figures 1L–1Q). While Olig2^+^ cells were reduced in Notch-On mice, the overall number of ventral NPCs expressing Nkx6.1 increased by ∼50% (Figure 2M). The loss of Olig2 from Nkx6.1^+^ NPCs coincided with the increased expression of the p3 determinant Nkx2.2 (Figures 2A–2H and 2N). Given that Nkx2.2 can repress Olig2 (Mizuguchi et al., 2001; Novitch et al., 2001; Sun et al., 2003), the loss of pMN cells in Notch-On mice is likely due to their transformation toward the more ventral p3 fate. This conclusion was supported by the reduced percentage of Nkx6.1^+^ progenitors expressing Nkx2.2 and corresponding increase in Olig2^+^ cells seen in Notch-Off spinal cords (Figures 2I–2L and 2N). Collectively, these data demonstrate that Notch signaling plays a critical role enhancing the ventral character of NPCs and influencing their partitioning between pMN and p3 identities.

### Notch-Mediated Changes in Ventral NPCs Alter Neuronal and Glial Fates

We next used *R26R*^*GFP*^ lineage tracing to assess the fate of the Notch-manipulated cells. Consistent with the loss of Olig2, Notch-On spinal cords exhibited an ∼35% reduction in MN formation (Figures S3A–S3F and S3J–S3L). Most of this deficit resulted from the selective loss of Foxp1^+^ lateral motor column (LMC) MNs at limb levels and preganglionic column (PGC) MNs at thoracic levels, with little change to Foxp1^−^ medial and hypaxial motor column (MMC and HMC) MNs (Figure S3K) (Rousso et al., 2008). LMC and PGC MNs are among the last MN subtypes to be formed (Tsuchida et al., 1994), suggesting that Notch activity must be silenced for the generation of these later-born cell types. Nevertheless, Notch-Off spinal cords did not exhibit any obvious defects in either MN formation or segregation into different columnar subgroups (Figures S3G–S3L).

**Figure 3:**
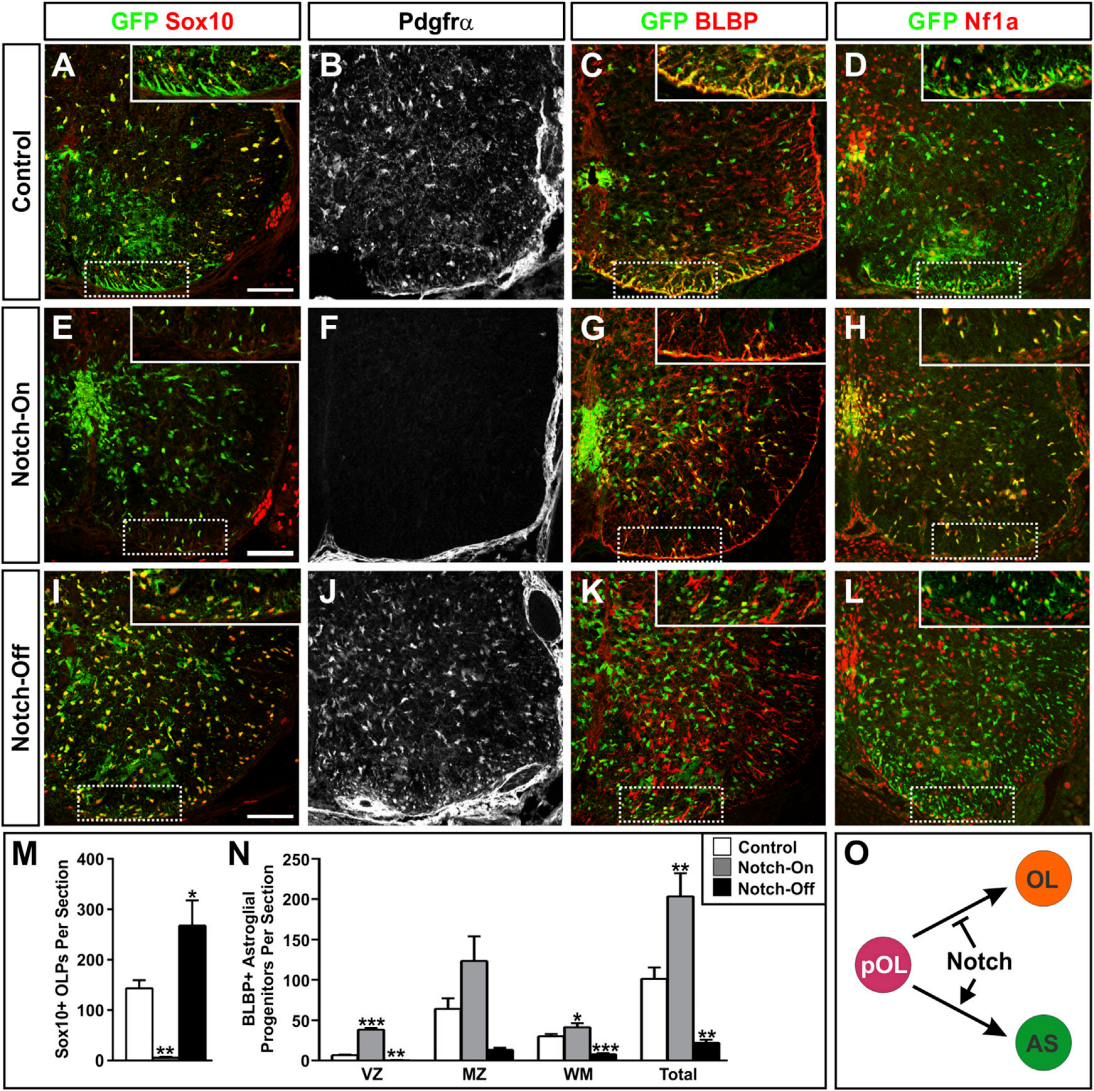
Manipulation of Notch Signaling Alters Glial Fates (A–D) In E18.5 control spinal cords, *Olig2*^*Cre*^;*R26R*^*GFP*^-labeled descendants include Sox10^+^/PDGFRα^+^ oligodendrocyte precursors (OLPs), BLBP^+^/NF1a^+^ pVA3 astrocyte progenitors. (E–H) Notch activation suppresses OLP formation and expands pVA3 progenitors. (I–L) Notch inactivation expands OLP production at the expense of pVA3 progenitors. Scale bars represent 100 μm. (M and N) Quantification of total OLP (GFP+/Sox10^+^) and pVA3 astrocyte progenitors (GFP+/BLBP^+^) per spinal cord half. pVA3 counts are divided based on localization within the VZ, marginal zone (MZ), or white matter (WM). Plots show the mean ± SEM from multiple sections collected from three to seven embryos for each group. ^∗^p < 0.05, ^∗∗^p < 0.01, ^∗∗∗^p < 0.001. (O) Summary of the role of Notch signaling in directing glial fate choices. See also Figures S3 and S4.

*Olig2*^*Cre*^-mediated Notch manipulations produced more striking changes in glial fate selection. In E18.5 control embryos, *Olig2*^*Cre*^ derivatives include both Sox10^+^ Pdgfrα^+^ oligodendrocyte progenitors scattered throughout the spinal cord and BLBP^+^ Nf1A^+^ Nkx6.1^+^ Fgfr3^+^ Slit1^+^ VA3 astrocyte precursors and differentiated astrocytes located in the ventral-most white matter (Figures 3 and S3M–S3U) (Hochstim et al., 2008). Notch-On spinal cords exhibited a nearly complete loss of pOLs and corresponding increase in VA3-like astrocyte precursors (Figures 3A–3H, 3M–3O, and S3M–S3R) (Hochstim et al., 2008). Conversely, Notch-Off spinal cords produced more pOLs and fewer astrocyte precursors and differentiated VA3 astrocytes (Figures 3I–3O and S3S–S3U). Together, these data show that early changes in NPC fates following Notch pathway manipulation lead to corresponding alterations in neuronal and, more strikingly, glial identities.

### Notch Signaling Is Only Able to Shift NPC Identities within the Ventral Spinal Cord

Previous studies observed that glial fates could be altered by deleting *Rbpj* function from all spinal NPCs (Taylor et al., 2007), raising the question of whether our results stemmed from direct effects of Notch activity on glial fate selection or were a secondary consequence of altered dorsoventral patterning. To distinguish between these possibilities, we examined the consequences of manipulating Notch activity in the p0 domain of the intermediate spinal cord using a *Dbx1*^*Cre*^ driver (Bielle et al., 2005; Dessaud et al., 2010). *Dbx1*^*Cre*^-mediated Notch activation expanded the numbers of Dbx1^+^ and Dbx2^+^ progenitors (Figures S4A–S4D and S4G), while Notch inactivation disrupted neuroepithelial organization and depleted these cells (Figures S4E–S4S). Despite these effects, we observed no changes in the dorsoventral register of NPCs or shifts in glial identities as seen with *Olig2*^*Cre*^-based manipulations (Figures S4T–S4AI). Thus, while manipulation of the Notch pathway can change the balance between NPC maintenance and differentiation within the intermediate spinal cord, it appears insufficient to evoke changes in dorsoventral patterning and associated shifts in neuronal and glial fates.

### Notch Signaling Alters Ventral Progenitor Identities by Modulating Responses to Shh

**Figure 4:**
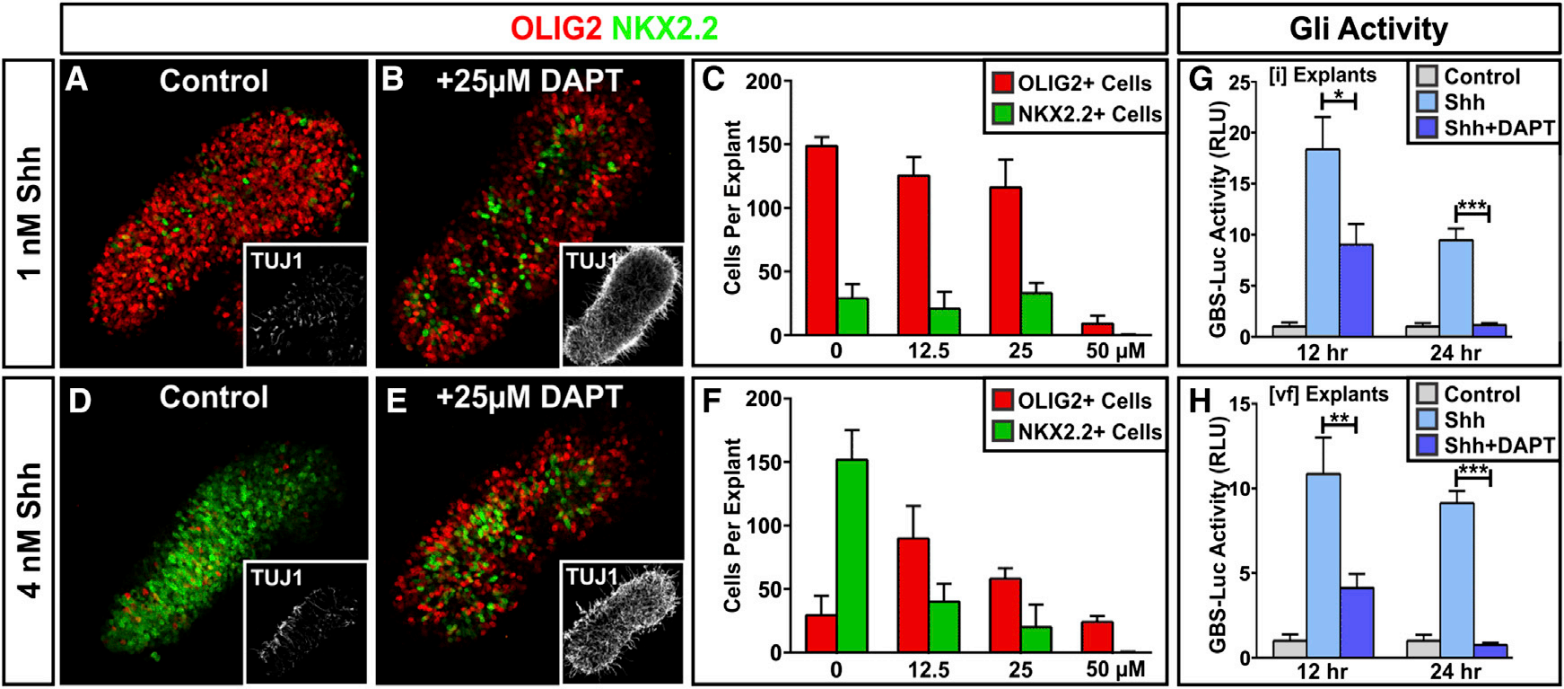
Inhibition of Notch Signaling Reduces Gli Activity and Assignment of the p3 Fate (A, B, D, and E) Representative images of HH stage 10 chick intermediate neural plate [i] explants cultured for 24 hr in 1- or 4-nM Shh ± 25-μM DAPT. Explants were stained with Nkx2.2 and Olig2 antibodies to identify p3 and pMN cells. Insets show DAPT addition increases Tuj1^+^ neurons, as expected for a Notch inhibitor. (C and F) Quantification of p3 and pMN cells present in [i] explants cultured in either 1- or 4-nM Shh and varying amounts of DAPT (0–50 μM). n ≥ 5 explants per condition and plots display cells/explant ± SEM. (G) Gli activity measurements of [i] explants isolated from chick embryos electroporated with a GBS-Luciferase reporter construct and cultured with or without 4-nM Shh ± 25-μM DAPT. n ≥ 5 explants per condition were collected; plots display relative GBS-luciferase activity (relative light units) ± SEM. (H) Gli activity measurements in [vf] explants isolated from embryos electroporated with the GBS-luciferase reporter and cultured in the presence or absence of 25-μM DAPT. n ≥ 5 explants per condition; relative GBS-luciferase activity ± SEM. ^∗^p < 0.05, ^∗∗^p < 0.01, ^∗∗∗^p < 0.001.

The selective effects of Notch activity on cell fate assignment in the ventral versus intermediate spinal cord suggests that Notch modulates the responsiveness of NPCs to Shh ligand produced at the ventral midline. To test this possibility, we used a chick intermediate [i] neural plate explant system to examine the fates of NPCs exposed to moderate (1 nM) or high (4 nM) amounts of Shh and varying amounts of the γ-secretase inhibitor DAPT (N-[N-(3,5-Difluorophenacetyl)-L-alanyl]-S-phenylglycine t-butyl ester) to reduce Notch receptor cleavage and downstream signaling (Dovey et al., 2001; Geling et al., 2002; Dessaud et al., 2007). High amounts of Shh produced numerous Nkx2.2^+^ p3 cells and a small number of Olig2^+^ pMN cells (Figure 4D), as previously described (Dessaud et al., 2007). However, when Notch activity was reduced using DAPT, the number of Nkx2.2^+^ progenitors was reduced while Olig2^+^ cells increased (Figures 4E and 4F), recapitulating the phenotype seen in Notch-Off mice (Figures 2I, 2J, and 2N). Interestingly, the effects of DAPT up to 25 μM appeared selective, as it blunted the Nkx2.2-inducing activity of high doses of Shh but did not block the Olig2-inducing activity of lower doses of Shh (Figures 4A–4C). These results suggest Notch is required for NPCs to experience high but not low levels of Shh signaling.

To verify that these NPC identity shifts were due to effects of Notch on Shh pathway activity, [i] explants were isolated from chick embryos electroporated with a Gli binding site-luciferase (GBS-luciferase) reporter to measure Gli function after Shh administration (Stamataki et al., 2005; Dessaud et al., 2007). DAPT addition led to a >50% decrease in GBS-luciferase activity over that seen with Shh alone (Figure 4G). Similar results were obtained with measurement of GBS-luciferase activity in ventral neural plate plus floor plate [vf] explants, in which Gli activity is driven by the endogenous Shh produced by floor plate cells (Figure 4H). Collectively, these data demonstrate that Notch signaling is required for NPCs to attain the highest levels of Gli activity and assume the ventral-most fates.

### Notch Signaling Facilitates the Accumulation of Smo within Primary Cilia

**Figure 5:**
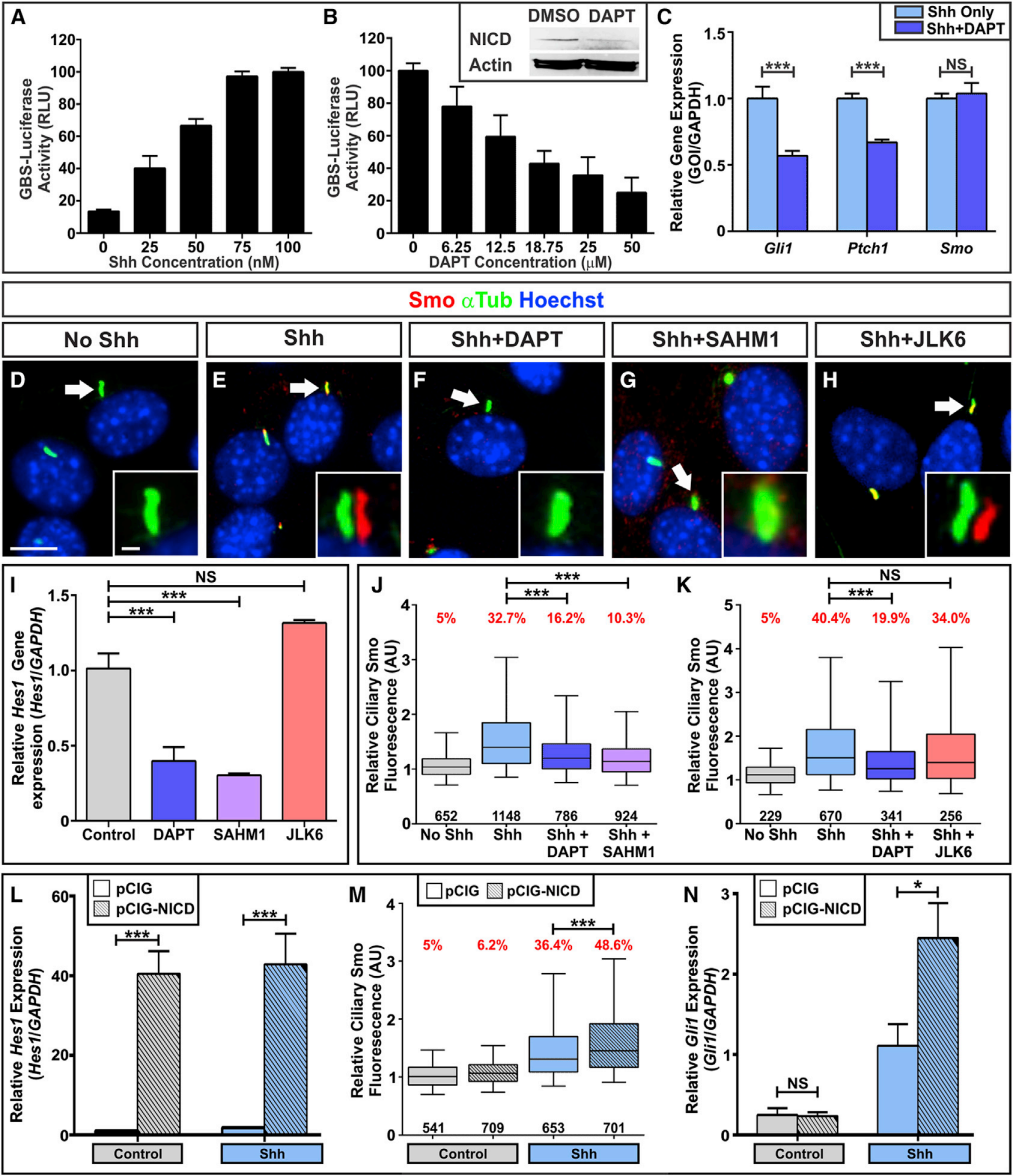
Notch Signaling Regulates the Ciliary Location of Smo and Shh Pathway Activity in Fibroblasts (A and B) GBS-luciferase reporter activity in NIH 3T3 Shh-LIGHT2 cells cultured in either Shh (0–100 nM) or a range of DAPT (0–50 μM) in the presence of a single concentration of Shh (50 nM). Points represent mean GBS-luciferase activity (relative light units) ± SEM from four to six independent samples. Inset shows immunoblotting for cleaved NICD and actin. (C) qPCR analysis of *Gli1*, *Ptch1*, and *Smo* expression in NIH 3T3 cells cultured in Shh (50 nM) ± DAPT (18.75 μM). Plot shows mean *Gapdh*-normalized gene expression levels ± SEM from six samples. Not significant (NS), p > 0.05, ^∗∗∗^p < 0.001. (D–H) Changes in the localization of Smo to primary cilia of NIH 3T3 cells treated with Shh and Notch inhibitors (DAPT, 18.75 μM and SAHM1, 20 μM) or a γ-secretase inhibitor that spares Notch function (JLK6, 20 μM). Cells were immunostained for αTubulin (αTub) (green), Smo (red), and Hoechst (blue, nuclei). Arrows denote cilia in the insets where Smo and αTub channels are offset to show colocalization. Low- and high-magnification scale bars represent 10 and 1 μm. (I) qPCR analysis of *Hes1* in NIH 3T3 cells exposed to DAPT (18.75 μM), SAHM1 (20 μM), or JLK6 (20 μM). Plots show mean *Gapdh*-normalized mRNA expression levels relative to unstimulated controls ± SEM from three to five samples. ^∗^p < 0.05, ^∗∗^p < 0.01, ^∗∗∗^p < 0.001. (J and K) Box and whisker plots of Smo fluorescence in the cilia of NIH 3T3 cells treated as indicated. The number of cilia analyzed in each group is indicated in black. The percentage of cilia with Smo is indicated in red. NS, p > 0.05, ^∗∗∗^p < 0.001. (L and N) qPCR analysis of *Hes1* and *Gli1* in NIH 3T3 cells transiently transfected with pCIG or pCIG-NICD vectors and then cultured in the presence or absence of Shh (50 nM). Plots show mean *Gapdh*-normalized expression levels relative to pCIG controls ± SEM from five to six samples for each condition. (M) Box and whisker plots of the ciliary Smo fluorescence in transfected cells. NS, p > 0.05, ^∗^p < 0.05, ^∗∗∗^p < 0.001. See also Figures S5 and S6.

We next sought to determine a mechanism that could explain the modulatory effects of Notch signaling on Shh responsiveness. Given that the requirement of Notch for Shh responses appears to be conserved in NPCs across species, we tested whether it was also conserved across cell types. NIH 3T3 mouse fibroblasts are a cell line shown to be Notch responsive (Small et al., 2003) and in which the cellular and molecular details of Shh signaling are well established (Taipale et al., 2000; Rohatgi et al., 2007; Tukachinsky et al., 2010). We first validated the system by exposing Shh-Light2 cells, a NIH 3T3 derivative stably transfected with a GBS-luciferase reporter, to increasing concentrations of Shh and observed dose-dependent increases in luciferase activity (Figure 5A). The addition of DAPT to these cultures strikingly reduced Shh-induced GBS-Luciferase activity (Figure 5B), recapitulating the effects seen with neural plate explants (Figures 4D–4H). Quantitative PCR (qPCR) analysis showed that DAPT similarly impacted endogenous Shh response genes such as *Gli1* and *Ptch1* (Figure 5C).

We then used the NIH 3T3 fibroblast system to pinpoint where Notch activity acts in the Shh transduction cascade. One of the first steps is the translocation of Smo to primary cilia, which initiates the conversion of Gli proteins into transcriptional activators (Corbit et al., 2005; Rohatgi et al., 2007). DAPT dramatically reduced Shh-induced Smo accumulation within primary cilia, acting in a dose-dependent manner (Figures 5D–5F, 5I–5K, and S5A). This change occurred without any obvious impact on *Smo* mRNA, alterations in cell polarity, or presence of primary cilia, although DAPT addition alone reduced average cilia length by 12.6% ± 1.3%, p < 0.001 (Figures 5C and S5B–S5I). To confirm that reductions in ciliary Smo were due to changes in Notch pathway activity, we repeated these experiments using two additional small molecule inhibitors: SAHM1, a peptide that prevents assembly of the NICD-Rbpj-MAML1 transcriptional activator complex (Moellering et al., 2009), and JLK6 (7-Amino-4-chloro-3-methoxyisocoumarin, also referred to as γ-secretase inhibitor XI), a molecule that blocks activation of some γ-secretase targets such as beta-amyloid precursor proteins while sparing others, including the Notch receptors (Petit et al., 2001). Verifying these activities, we found that both DAPT and SAHM1 reduced *Hes1* gene expression in NIH 3T3 cells by ∼65%–75%, whereas JLK6 had no discernible effect (Figure 5I). Importantly, SAHM1 reduced Shh-induced ciliary accumulation of Smo in a manner similar to DAPT (Figures 5G and 5J). JLK6 in contrast had no effect on Smo localization (Figures 5H and 5K).

We further tested whether the impact of Notch activity on Shh-induced Smo localization was limited to NIH 3T3 cells or more broadly applicable to other cell types including human NPCs, primary mouse embryonic fibroblasts (MEFs), and C2C12 mouse myoblasts. In all cases, DAPT reduced Shh-induced Smo accumulation within primary cilia (Figures S6A–S6M), suggesting that the cross-talk between the Notch and Shh pathways is conserved across germ layers and species.

Since Notch inhibition reduced both the presence of Smo within primary cilia and Shh pathway activity, we tested whether the converse was also true. NIH 3T3 cells were transiently transfected with a vector expressing *NICD* and an IRES-*nEGFP* reporter cassette to activate Notch signaling, and both Smo localization and the expression of Shh-target genes evaluated. *NICD*-transfected cells exhibited an ∼40-fold increase in *Hes1* expression irrespective of Shh stimulation (Figure 5L). Primary cilia were also slightly longer (17.5% ± 3.9%, p < 0.001) in *NICD*-transfected cells compared with *nEGFP*-only transfection controls, consistent with the reduced cilia lengths seen with DAPT addition. Upon Shh treatment, *NICD*-transfected cells exhibited an increase in the level of Smo within primary cilia and ∼2- to 3-fold higher levels of *Gli1* expression (Figures 5M and 5N). These effects were only seen after the addition of Shh. Together, these results illustrate that Notch activity is not only required for Shh responsiveness, but can also potentiate its signaling function.

Given that *Hes1* was notably changed in all of our Notch manipulations, we tested whether direct elevation of *Hes1* could similarly increase cellular responses to Shh ligand. Interestingly, *Hes1* misexpression was sufficient to increase Shh-evoked activation of *Gli1* ∼1.8-fold (Figures S5J–S5K). Collectively, these results suggest that the potentiating effects of Notch on Shh signaling result from activation of Hes genes and likely other downstream effectors.

**Figure 6:**
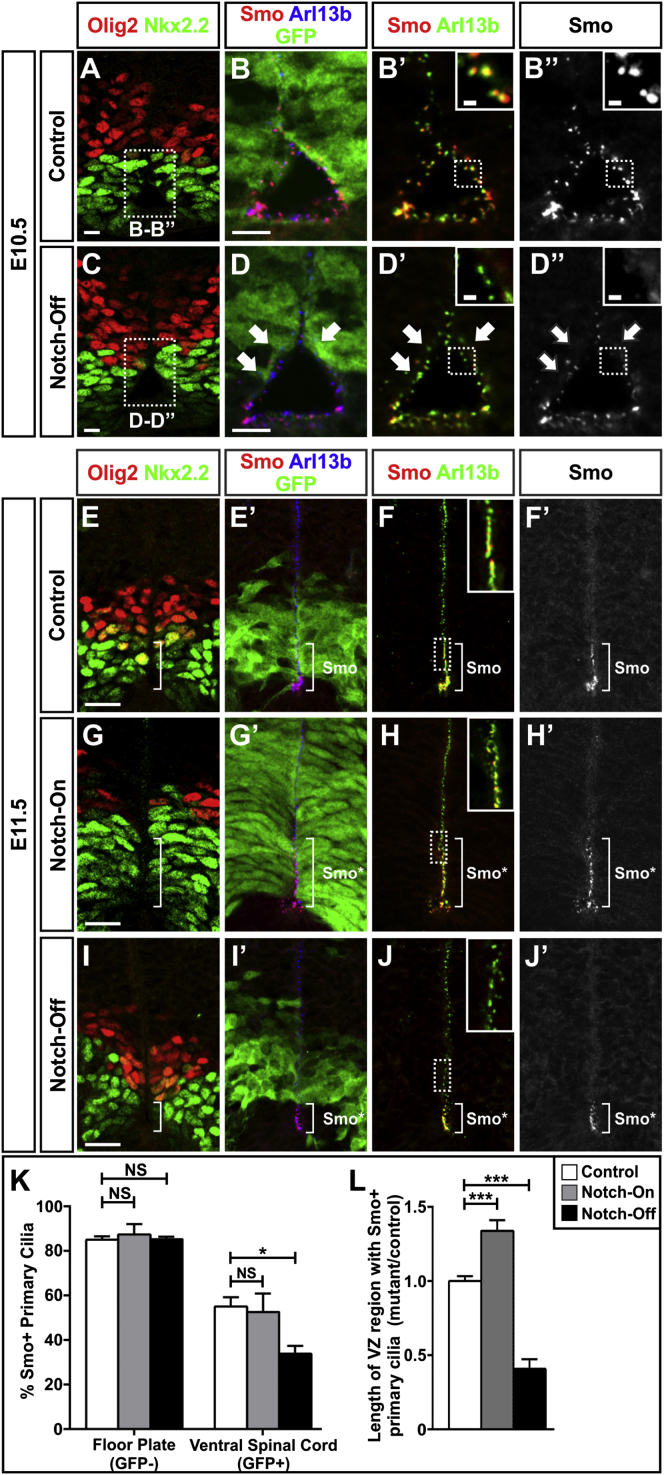
Notch Signaling Influences the Ciliary Accumulation of Smo in Ventral Spinal Cord NPCs (A–D′′) Analysis of Smo^+^ primary cilia present on ventral progenitors in E10.5 embryos. Arrows in (D) indicate regions of Cre recombination. In Notch-Off embryos, Smo is absent from cilia in the recombined regions. Low (A and C) and high (B and D) magnification scale bars represent 10 and 2 μm. (E–J′) Analysis of primary cilia in E11.5 embryos. Brackets illustrate the dorsoventral extent of Smo^+^ primary cilia, a region where Nkx2.2^+^ p3 cells are present. Scale bars represent 20 μm. (K) Quantification of Smo^+^ primary cilia at E10.5 counted from the GFP- floor plate and GFP^+^ ventral progenitors. Plots show the mean percentage of Smo^+^ primary cilia ± SEM from multiple sections collected from three to four embryos from each group. NS, p > 0.05 and ^∗^p < 0.05. (L) Quantification of the dorsoventral limits of Smo^+^ primary cilia at E11.5. Plots show mean lengths of the VZ lined with Smo^+^ cilia ± SEM. All measured lengths were normalized to littermate controls. Analysis was conducted on multiple sections collected from three to nine embryos from each experimental group. ^∗∗∗^p < 0.001. See also Figure S7.

Given the ability of Notch signaling to promote localization of Smo to cilia in cultured cells, we examined whether this effect could also be seen in the developing spinal cord. In E10.5 control embryos, high amounts of Smo were present in the cilia of both floor plate and Nkx2.2^+^ p3 cells and lower levels present in Olig2^+^ pMN cells (Figures 6A–6B′′). In Notch-Off spinal cords, most *Olig2*^*Cre*^-derived NPCs exhibited lower levels of ciliary Smo, and this change preceded shifts in Olig2 and Nkx2.2 expression (Figures 6C–6D′′ and 6K). By E11.5, the extent of Smo localization within cilia along the dorsoventral axis of Notch-Off mutants was reduced by ∼60% compared with littermate controls (Figures 6E–6F′ and 6I–6L). Notch-On mutants by contrast showed a dorsal expansion in the extent of Smo localization within primary cilia (Figures 6G–6H′ and 6L).

Changes in the ciliary accumulation of Smo following Notch manipulations could stem from either direct effects of Notch on Smo trafficking or indirect effects related to Notch having altered NPC identities. To distinguish between these possibilities, we examined Smo staining in the spinal cords of *Nkx2.2*, *Olig2*, and *Pax6* mutant mice, where dorsoventral patterning is known to be severely disrupted (Dessaud et al., 2008). Remarkably, the dorsal limits of ciliary Smo in all mutants were similar to control littermates, despite clear changes in NPC fates (Figures S7A–S7R). In *Nkx2.2* mutants, this alteration permitted the unusual presence of Olig2 in cells exhibiting high amounts of Smo in their cilia (Figures S7J and S7N), a phenotype that was never seen in control embryos or those in which Notch activity had been manipulated (Figures 6E–6L). Collectively, these data show that Notch activity influences Smo accumulation within primary cilia in multiple cell types in vitro and spinal cord NPCs in vivo and acts upstream of the transcription factor network controlling dorsoventral fates.

### Notch Activity Sets the Levels of Ptch1 Present in Primary Cilia, Thereby Gating Smo Entry

**Figure 7:**
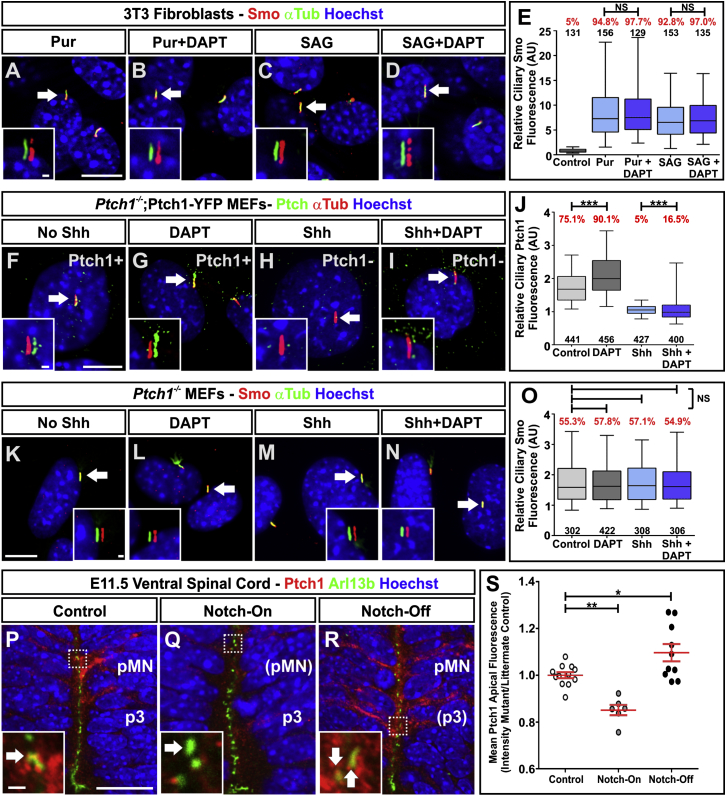
Notch Signaling Regulates Ptch1 Presence in and around Primary Cilia (A–D) Analysis of Smo enrichment in primary cilia of NIH 3T3 cells treated with Pur (5 μM) or SAG (1 μM) ± DAPT (18.75 μM). Arrows denote cilia shown in the insets, in which Smo and αTub are offset to show colocalization. Low- and high-magnification scale bars represent 10 and 1 μm. (E) Box and whisker plots of Smo fluorescence in the cilia of NIH 3T3 cells treated with Pur or SAG ± DAPT. The black numbers indicate the number of cilia analyzed. The red numbers indicate the percentage of cilia with Smo. NS, p > 0.05. (F–I) Ciliary enrichment of Ptch1 in *Ptch1*^*−/−*^*;Ptch1-YFP* MEFs after exposure to DAPT (18.75 μM) with or without Shh (50 nM). Low- and high-magnification scale bars represent 10 and 1 μm. (J) Box and whisker plots of Ptch1 fluorescence in the cilia of *Ptch1*^*−/−*^*;Ptch1-YFP* MEFs. ^∗∗∗^p < 0.001. (K–N) Analysis of Smo localization in *Ptch1*^*−/−*^ MEFs treated with or without Shh (50 nM) ± DAPT (18.75 μM). Arrows denote cilia shown in the insets, in which Smo and αTub channels are offset to show colocalization. Scale bars represent 10 and 1 μm (insets). (O) Box and whisker plots of Smo fluorescence in the cilia of *Ptch1*^*−/−*^ MEFs treated with or without Shh (50 nM) ± DAPT (18.75 μM). NS, p > 0.05. (P–R) Apical Ptch1 staining in the ventral spinal cord of E11.5 embryos. The pMN and p3 labels were determined by serial section staining for Olig2 and Nkx2.2 (not shown). Insets show Ptch1 presence in Arl13b-stained primary cilia. Scale bars represent 20 and 1 μm (insets). (S) Scatterplot of the mean intensity of apical Ptch1 staining in a 250 μm^2^ area ± SEM. Each point represents the mean intensity from multiple sections collected from a single embryo. Each group is comprised of data from 6–12 embryos. The intensity of Ptch1 was normalized to littermate controls. ^∗^p < 0.05, ^∗∗^p < 0.01. See also Figure S8.

We next considered the mechanism by which Notch might impact Smo localization. Our observations that Notch activation only promoted the accumulation of Smo within cilia following Shh addition suggested that it most likely acts upstream of Smo in the Shh transduction cascade. Consistent with this model, we found that DAPT was unable to block Smo accumulation when cells were treated with either Purmorphamine (Pur) or Smoothened Agonist (SAG), small molecules that directly stimulate Smo activity in a Shh ligand-independent manner (Chen et al., 2002; Sinha and Chen, 2006) (Figures 7A–7E). We thus focused our attention on the actions of Notch on the Shh receptor Ptch1. In the absence of ligand, Ptch1 localizes around the base and within primary cilia, where it inhibits Smo entry and Gli activation (Rohatgi et al., 2007). Shh binding to Ptch1 promotes its exit from primary cilia and concomitant Smo accumulation (Rohatgi et al., 2007). Since endogenous Ptch1 protein was difficult to detect in NIH 3T3 cells by antibody staining, we utilized Ptch1-YFP MEFs generated by infection of *Ptch1*^*LacZ/LacZ*^ mutant cells with a retrovirus expressing a Ptch1-YFP fusion protein (Rohatgi et al., 2007). In the absence of Shh, ∼75% of primary cilia contained Ptch1 (Figures 7F and 7J). When DAPT was added for 12 hr, the number of Ptch1^+^ primary cilia increased to ∼90% (Figures 7G and 7J). DAPT was also able to impede the clearance of Ptch1 from primary cilia upon Shh stimulation (Figure 7H–7J). Remarkably, the effects of DAPT on Ptch1 localization occurred without any change in either *Ptch1* mRNA or protein levels in both Ptch1-YFP MEFs and NIH 3T3 cells (Figures S8G–S8I).

These results prompted us to examine whether the effects of DAPT on Smo trafficking to primary cilia occur immediately after its addition or rather require more time to enable Ptch1 to increase and thereby block Smo entry. Smo normally accumulates in primary cilia within 4 hr of Shh addition (Rohatgi et al., 2007) (Figures S8A and S8B). When Shh and DAPT were coadministered, there was no decrease in Smo presence within primary cilia at either the 4- or 6-hr time points; rather, Smo reduction only became evident after ∼12 hr (Figures S8A and S8B). In contrast, when cells were pretreated with DAPT for 8 hr and then exposed to Shh plus DAPT for an additional 4 hr, significant reductions in Smo ciliary accumulation were observed (Figures S8C and S8D). These data indicate that the suppressive actions of DAPT on Smo localization follow the time course of Ptch1 accumulation within primary cilia. We further found that the actions of DAPT required new transcription, as changes in Smo localization were partially blocked by coadministration of DAPT and the RNA polymerase inhibitor α-amanitin (Figures S8E and S8F). These results suggest that Notch modulates Ptch1 and Smo levels in and around primary cilia through a transcriptional mechanism.

To test whether Ptch1 mediates the inhibitory effects of DAPT on Smo, we measured the impact of DAPT addition to *Ptch1*^*LacZ/LacZ*^ mutant MEFs. Whereas DAPT potently inhibited Smo accumulation in the cilia of Shh-treated control MEFs, it was unable to do so in *Ptch1* null cells (Figures 7K–7O and S8J). Collectively, these data show that Notch signaling influences Smo accumulation by regulating the ciliary presence of Ptch1.

Finally, we tested whether altered localization or abundance of Ptch1 protein was observed after manipulations of the Notch pathway in the ventral spinal cord. In Notch-On mutants, Ptch1 protein staining in and around the primary cilia was notably reduced, fitting with the observed increase in Smo presence (Figures 6E–6H′, 7P, 7Q, and S7). In contrast, Notch-Off mutants showed elevated Ptch1 at the apical membrane and cilia in accordance with the reductions in Smo staining (Figures 6I–6J′, 7R, and 7S). Together, these in vitro and in vivo experiments demonstrate that Notch signaling plays an integral role modulating Ptch1 localization to gate Smo entry into primary cilia. Through these actions, Notch can regulate the downstream activation of the Shh transduction pathway and assignment of NPC fates.

## Discussion

**Figure 8:**
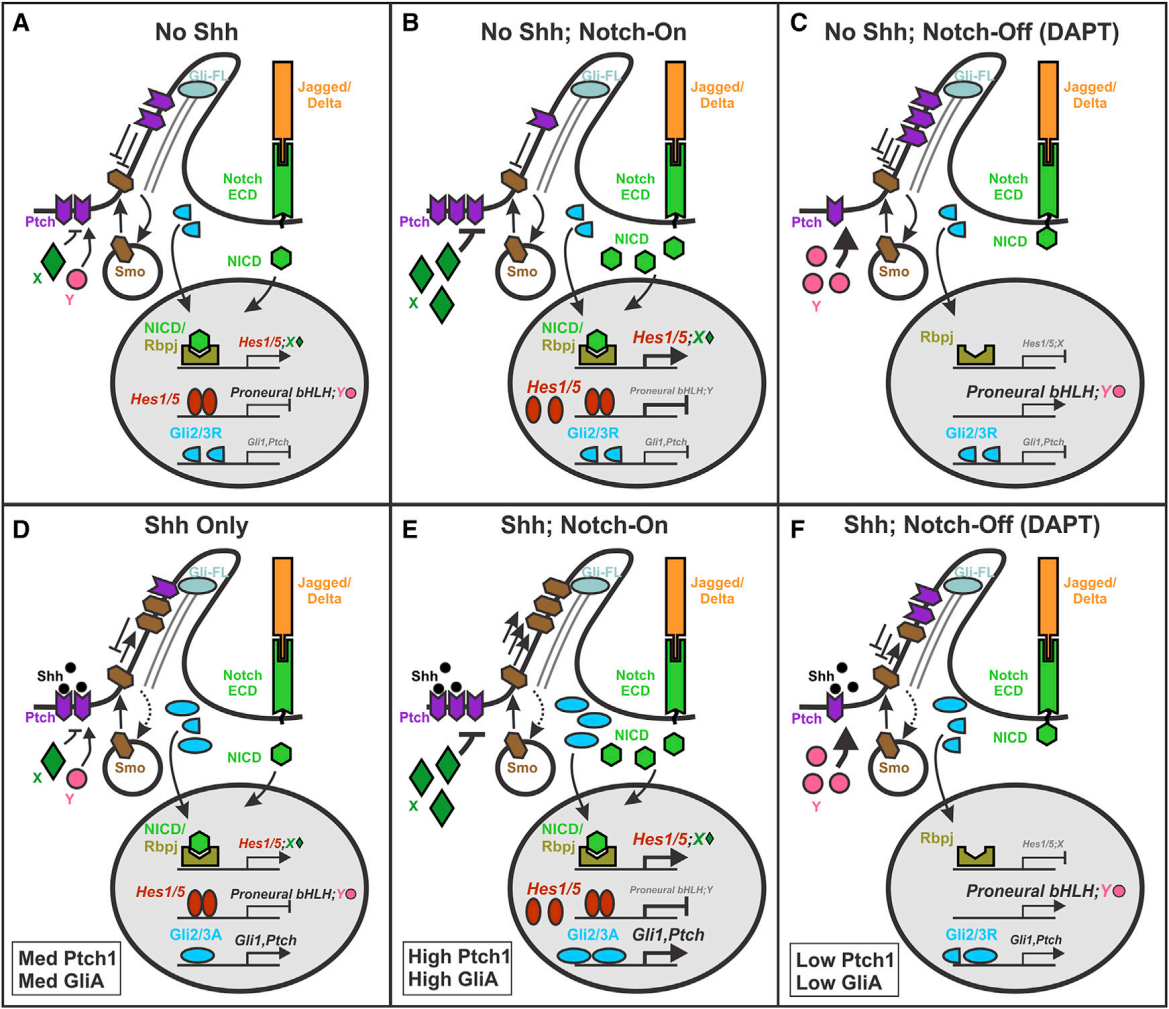
Models for Interactions between Notch and Shh Signaling Models depicting how Notch signaling modulates cellular responses to Shh by regulating the movement of Ptch1 to primary cilia. (A and D) In the absence of Shh, Ptch1 is present within and adjacent to primary cilia. Upon addition, Shh ligand binds to Ptch1, permitting Smo entry into the cilia where it stimulates Gli transcriptional activities. Direct downstream effectors of Notch signaling that promote Ptch1 clearance from primary cilia (X) and indirect effectors suppressed by Hes genes (Y) that increase Ptch1 ciliary accumulation are depicted. (B and E) Notch activation via the ectopic expression of NICD reduces Ptch1 presence within primary cilia facilitating Smo entry and activation of Gli proteins. (C and F) Notch inhibition, via the addition of DAPT or removal of Rbpj, elevates the presence of Ptch1 within primary cilia. Smo entry is impeded and Gli activities correspondingly reduced.

It is well established that the dorsoventral identity of NPCs in the spinal cord and other regions of the CNS is influenced by the concentration of Shh ligand they are exposed to (Fuccillo et al., 2006; Dessaud et al., 2008; Ribes and Briscoe, 2009). However, Shh concentration is only part of the means though which graded signaling responses are achieved. Other important factors include (1) the duration of time over which cells are exposed to Shh, (2) the ability of cells to modulate their responsiveness to Shh through changes in the expression and/or subcellular distribution of key signal transduction components such as Ptch1 and Smo, (3) changes in the expression of proteins that modulate Shh-Ptch1 interactions or modify Shh itself, and (4) cross-regulatory interactions between Shh-regulated transcription factors that assign specific cell fates (Dessaud et al., 2008; Ribes and Briscoe, 2009; Briscoe and Thérond, 2013). Our studies show that Notch signaling plays a crucial role in these first two processes, serving to sustain NPCs in an undifferentiated, Shh-responsive state while also influencing the ciliary trafficking of Ptch1 and Smo and the downstream activation of Gli transcription factors (Figure 8). Together, these data provide important insights into the mechanisms through which NPCs interpret the Shh gradient and reveal a potentially general mechanism by which the Notch and Shh signaling pathways collaborate to direct cell fate decisions.

### Notch-Mediated Changes in Shh Transduction Influence the Selection of NPC Fates

Our data show that manipulating the Notch pathway modulates the dorsoventral register of NPCs, with Notch activation and inactivation respectively increasing or decreasing the formation of the ventral-most cell types reflected by alterations in Nkx2.2 and Olig2 expression and shifts in specific classes of neurons and glia. Importantly, multiple lines of evidence indicate that these changes are due to the ability of Notch to modulate how NPCs interpret the endogenous Shh signaling gradient rather than more direct effects on cell fate determination. First, all changes in NPC fates occurred within the context of Nkx6.1^+^ progenitors, which reflect the limit of endogenous Shh signaling in the spinal cord (Briscoe et al., 2000). Second, Notch manipulation in the intermediate spinal cord impacted NPC maintenance without any change in dorsoventral patterning or shift in glial cell types. Third, in fibroblasts, Notch activation and inactivation were unable to modulate Smo trafficking to primary cilia or Gli transcriptional activity without the coadministration of Shh ligand. Collectively, these data indicate that Notch plays a supporting role in tissue patterning by tuning the response of cells to Shh present in the developing embryo or culture media.

It has long been appreciated that the influences of Shh on neural fate selection are generally restricted to dividing cells (Ericson et al., 1996). Recent studies have provided molecular explanations for this relationship showing that most Shh/Gli-regulated genes are coregulated by SoxB1 transcription factors such as Sox2 that are broadly expressed by NPCs (Oosterveen et al., 2012; Peterson et al., 2012; Oosterveen et al., 2013). Some of the positive effects of Notch on Shh signaling could thus be accounted for by its ability to elevate SoxB1 levels as it maintains NPCs in an undifferentiated state. However, our data indicate that Notch can also act at a more proximal level, regulating the ciliary localization of at least two key components of the Shh transduction pathway, Ptch1 and Smo. Ptch1 appears to be the most directly impacted by Notch, as the addition of DAPT alone to fibroblasts promotes Ptch1 accumulation within primary cilia (Figures 7F, 7G, and 7J), and Ptch1 is known to block Smo entry and downstream signaling events (Rohatgi et al., 2007). Moreover, DAPT was unable to reduce Smo accumulation within cilia in the absence of Ptch1 or in the presence of Pur and SAG, small molecules that bypass Ptch1 function (Figures 7A–7E and 7K–7O). These observations in fibroblasts also hold true for spinal cord NPCs, as *Rbpj* deletion increased Ptch1 protein in and around primary cilia, whereas NICD misexpression reduced it. These changes correspondingly impacted Smo presence in cilia and, ultimately, the expression of specific NPC fate determinants (Figures 7P–7S).

### Notch as a Modulator of Ciliary Trafficking

How might Notch signaling alter Ptch1 and Smo trafficking? In epidermal cells, Notch receptors and processing enzymes are located in and adjacent to primary cilia, and ciliary transport is required for Notch pathway activity (Ezratty et al., 2011). Based on this proximity, Notch signaling components could conceivably impact the interactions of ciliary transport proteins with Shh signaling components. However, our results point to Notch acting through a transcriptional mechanism. First, changes in NPC fates and Gli transcriptional activity were seen with either removal of Rbpj function or increased expression of NICD, components whose main sites of action are known to be in the nucleus. Second, the Shh-potentiating activities seen with NICD misexpression were recapitulated by the forced expression of Hes1, one of the best known downstream transcriptional effectors of the Notch pathway. Third, the effects of DAPT administration on Ptch1 and Smo trafficking were not immediate, but rather required at least 12 hr of exposure—more than sufficient time for a transcriptionally mediated response. Finally, DAPT effects on Smo trafficking were blocked by the addition of the transcriptional inhibitor α-amanitin. Together, these results lead us to propose that Notch and Hes genes modulate Shh signaling by regulating the expression of genes whose products impact the trafficking of Ptch1, Smo, and potentially other Shh signaling components to primary cilia, designated as “X” for direct Notch effectors and “Y” for Hes-suppressed effectors (Figure 8).

While a great deal is known about the transcriptional control of *Ptch1* in response to Shh pathway activation, relatively little is known about the regulation of Ptch1 protein trafficking. Some insights into this process have been recently made by observations that Ptch1 exit from primary cilia requires the function of the intraflagellar transport (IFT) protein Ift25 (Keady et al., 2012), and endocytic turnover mediated by the ubiquitin E3 ligases Smurf1 and Smurf2 (Yue et al., 2014). Loss of these components results in Ptch1 accumulation within primary cilia and reduced cellular responses to Shh (Keady et al., 2012; Yue et al., 2014), reminiscent of the effects seen with the loss of Notch signaling. However, none of these genes were changed by our Notch manipulations (J.H.K. and B.G.N., unpublished data). A better understanding of the downstream targets of Notch and Hes1 should yield important new insights into how the localization and function of Ptch1 and other Shh signaling components may be controlled.

### Is a Role for Notch Gating Responses to Other Developmental Signals Dependent on Cilia?

The primary cilium is a nonmotile organelle that is present on almost all vertebrate cells (Pazour and Witman, 2003). Although primary cilia were first observed over a century ago (Zimmermann, 1898), their function as an antenna-like organelle that allows cells to detect extracellular environmental stimuli and modulate an appropriate intracellular response has only recently been realized. In addition to Shh signaling, primary cilia are thought to be essential for Hippo, mTor, Notch, Pdgfrα, and Wnt signaling (Schneider et al., 2005; Boehlke et al., 2010; Ezratty et al., 2011; Habbig et al., 2011; Lancaster et al., 2011). The importance of primary cilia is perhaps best illustrated through ciliopathies, a group of genetic disorders that are due to defects in the generation or function of cilia, which collectively affect nearly every major organ in the human body (Novarino et al., 2011). As no protein synthesis occurs within the cilium, the formation of the cilium and the accumulation of signaling pathway components within the cilium are entirely dependent on the IFT system to shuttle proteins to their proper areas (Pedersen and Rosenbaum, 2008).

While our study focused on the impact of Notch on Shh signaling by altering the localization of Ptch1 and Smo, the mechanisms used to achieve this result are likely to have a broader impact on other signaling pathways that depend upon the IFT system. Consistent with this hypothesis, we have carried out a series of preliminary expression profiling experiments in NIH 3T3 cells, which indicate that DAPT addition reduces the expression of several proteins known to be associated with primary cilia (Ishikawa et al., 2012), including components of the Pdgfrα and Wnt signaling pathways, and various extracellular matrix proteins (J.H.K. and B.G.N., unpublished data). In this regard, the mechanism through which Notch gates the responsiveness of cells to Shh might signify a more general role for Notch modulating ciliary transport that could impact multiple signaling pathways involved in both development and disease.

## Experimental Procedures

### Animal Preparation and Tissue Analysis

*Olig2*^*Cre*^ and *Dbx1*^*Cre*^ mice were generated as previously described (Bielle et al., 2005; Dessaud et al., 2007). Cre mice were crossed with *R26R*^*GFP*^ transgenic reporter mice (B6;129-*Gt(ROSA)26Sor*^*tm2Sho*^*/*J; Jackson Labs Stock #004077) (Mao et al., 2001); *R26R*^*NICD-nGFP*^ transgenic floxed mice (*Gt(ROSA)26Sor*^*tm1(Notch1)Dam*^*/*J; Jackson Labs Stock #008159) (Murtaugh et al., 2003), or *Rbpj*^*CKO*^ mice (Han et al., 2002). *Olig2*^*−/−*^, *Nkx2.2*^*−/−*^, and *Pax6*^*Sey/Sey*^ mutant mice were generated as previously described (Novitch et al., 2001; Rousso et al., 2012). All mice were maintained and tissue collected in accordance with guidelines set forth by the UCLA Institutional Animal Care and Use Committee. Chick neural plate explants were generated as previously described (Dessaud et al., 2007). All spinal cord tissues were fixed, cryoprotected, sectioned, and processed for immunohistochemistry or in situ hybridization as previously described (Novitch et al., 2001; Gaber et al., 2013). Antibodies and probes used are listed in the Supplemental Experimental Procedures.

### Cell Culture and Primary Cilia Analysis

NIH 3T3 fibroblasts (CRL-1658) and C2C12 myoblasts (CRL-1772) were purchased from ATCC. Shh-LIGHT2 cells were used as previously described (Taipale et al., 2000). *Ptch1*^*−/−*^ and *Ptch1*^*−/−*^;Ptch1-YFP MEFs were generated as previously described (Rohatgi et al., 2007, 2009). Primitive human neuroepithelial progenitors were generated from embryonic stem cells as previously described (Hu et al., 2009). For cilia analysis in fibroblasts, cells were plated onto glass coverslips, grown to 80%–100% confluency in DMEM containing 10% bovine calf serum (BCS) and then changed to low serum media (0.5% BCS) at the beginning of experiments. Cells were fixed in 4% paraformaldehyde, incubated with indicated primary and secondary antibodies, and mounted in Prolong Gold (Invitrogen). See also Supplemental Experimental Procedures.

### Statistical Analyses

Unless otherwise stated, cell counts, luciferase assays, and qPCR analyses are presented as mean values ± SEM. For Figures 1Q, 2M, 2N, 3M, 3N, 5I, 6K, 6L, 7S, S3J, S3K, S4G, S4AF–S4AI, S7Q, S7R, and S8I, experimental conditions were compared with the control, and an ANOVA with a Dunnett’s post hoc test was performed. For the data shown in Figures 4G, 4H, 5C, 5L, 5N, S5D, S5I–S5K, and S8H–S8J, unpaired, two-tailed t test were performed. All ciliary Smo fluorescence data sets did not pass the Shapiro-Wilk normality test. Thus, for all ciliary Smo analyses between two groups (Figures 5M, 7E, 7J, S8B, S8D, and S8F) two-tailed nonparametric Mann-Whitney tests were performed. For analyses between three or more groups (Figures 5J, 5K, 7O, S5A, S6D, S6H, and S6L), nonparametric Kruskal-Wallis tests were used along with Dunnett’s post hoc tests. All statistical analyses were calculated using Graphpad Prism 6 software. Significance was assumed when p < 0.05.

## Author Contributions

J.H.K., L.Y., E.D., K.C., and D.M.M. performed the experiments. R.R. contributed vital reagents and insights. J.H.K., L.Y., J.B., and B.G.N. designed the experiments and wrote the paper.

## Supplemental Information

Document S1. Supplemental Experimental Procedures and Figures S1–S8

Document S2. Article plus Supplemental Information
