# Supplementary material for: Notch Activity Modulates the Responsiveness of Neural Progenitors to Sonic Hedgehog Signaling
Source: Dev Cell. 2015 May 26;33(4):373–87. doi: 10.1016/j.devcel.2015.03.005 (PMC4449290; doi:10.1016/j.devcel.2015.03.005)
Supplement: Document S1. Supplemental Experimental Procedures and Figures S1–S8 [file mmc1.pdf]

Developmental Cell
Supplemental Information

Notch Activity Modulates the Responsiveness of Neural Progenitors to Sonic Hedgehog Signaling

Jennifer H. Kong, Linlin Yang, Eric Dessaud, Katherine Chuang, Destaye M. Moore, Rajat Rohatgi, James Briscoe, and Bennett G. Novitch

Figure S1. Temporal and spatial documentation of *Olig2^{Cre}*-mediated recombination (related to Figure 1).

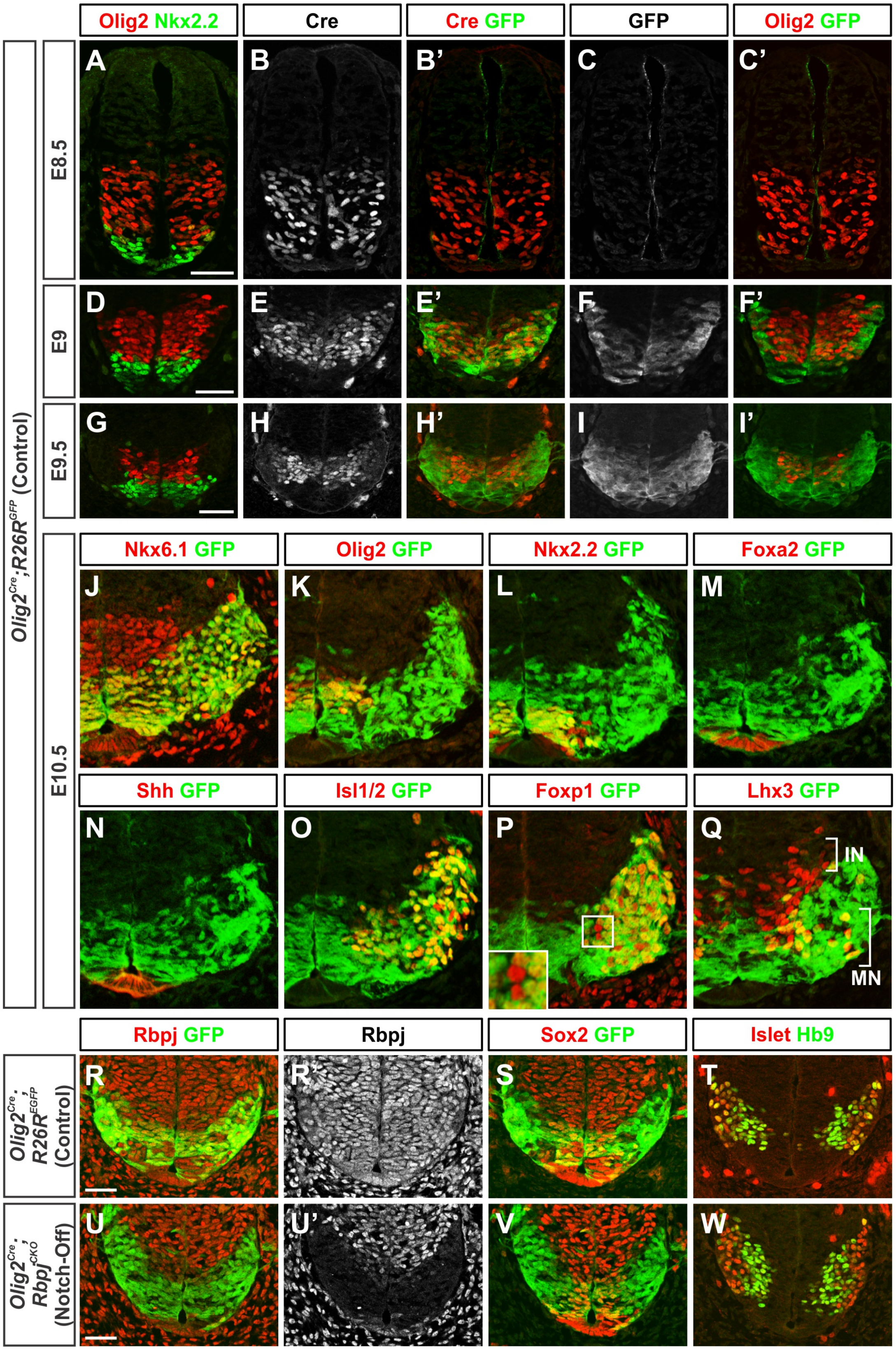

(A-I) Serial transverse spinal cord sections from E8.5-E9.5 Control (*Olig2^{Cre}; R26R^{GFP}*) embryos. Tissue was immunostained with Nkx2.2, Olig2, Cre, and GFP antibodies to assess where and when recombination occurred. Scale bars = 50 µm.
(J-Q) Analysis of E10.5 *Olig2^{Cre}; R26R^{GFP}* Control embryos shows that Cre recombination takes place in both the pMN (Nkx6.1^{+}/Olig2^{+}) and p3 (Nkx6.1^{+}/Nxk2.2^{+}) domains, but not in the floor plate (FoxA2^{+} or Shh^{+}). At this time point, Cre recombination had occurred in nearly all MNs (Isl1/2^{+}), including both LMC (FoxP1^{+}) and MMC (Lhx3^{+}) subgroups. Inset in (P) reveals a small population of non-recombined MNs. Note that Lhx3 is also prominently expressed by newborn V2 interneurons that form above the motor columns.
(R-W) Notch-Off embryos display an absence of Rbpj protein from pMN, p3 progenitors, and differentiated (Islet^{+}/Hb9^{+}) MNs starting around E9.5. Note that Sox2 is maintained in pMN/p3 cells despite the loss of Rbpj. Scale bars = 50 µm.

Figure S2. Notch signaling is activated in Notch-On embryos and reduced in Notch-Off embryos without any major disruptions to the neuroepithelial organization (related to Figures 1 and 2).

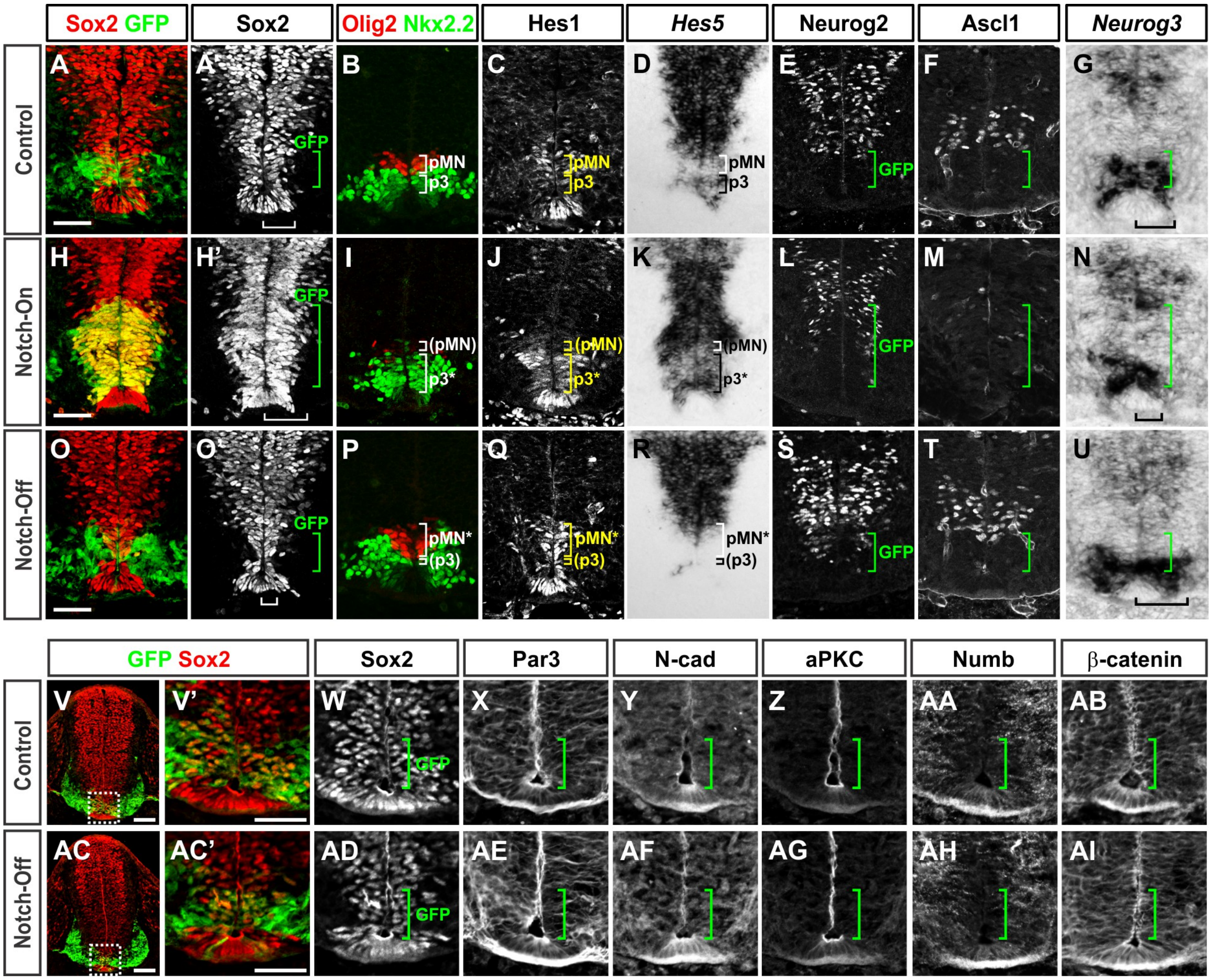

(A-U) Analysis of Notch signaling activity in Control (A-G), Notch-On (H-N), and Notch-Off (O-U) E11.5 embryos. (A, H, O) Green brackets demarcate the dorsoventral extent of the GFP^+ region of recombination and white brackets indicate the thickness of the $Sox2^+$ ventricular zone (VZ) within this same region. As predicted, NICD misexpression (Notch-On) expands the thickness of the $Sox2^+$ VZ, increases expression of canonical downstream Notch effectors (Hes1 and *Hes5*), and reduces expression of proneural transcription factors (Neurog2, Ascl1, and *Neurog3*). Conversely, Rbpj deletion (Notch-Off) has the opposite effect. Note, however, that Sox2 and Hes1 expression are maintained in the ventral spinal cord despite the removal of Rbpj (panels O and Q). Scale bars = 50 μm.

(V-AI) The neuroepithelial architecture and apicobasal polarity of neural progenitors is preserved in the ventral spinal cord of Notch-Off (AC-AI) E11.5 embryos. Within the region of recombination (marked by the green brackets), the lasting integrity of the ventricular zone is apparent through the maintenance of $Sox2^+$ progenitors and the continued presence of various cell polarity components (Par3, aPKC, and Numb) and cell adhesion molecules (N-cadherin and β-catenin). Scale bars = 100 μm.

Figure S3. The effect of Notch signaling manipulations on MN and glial cell fates (related to Figure 3).

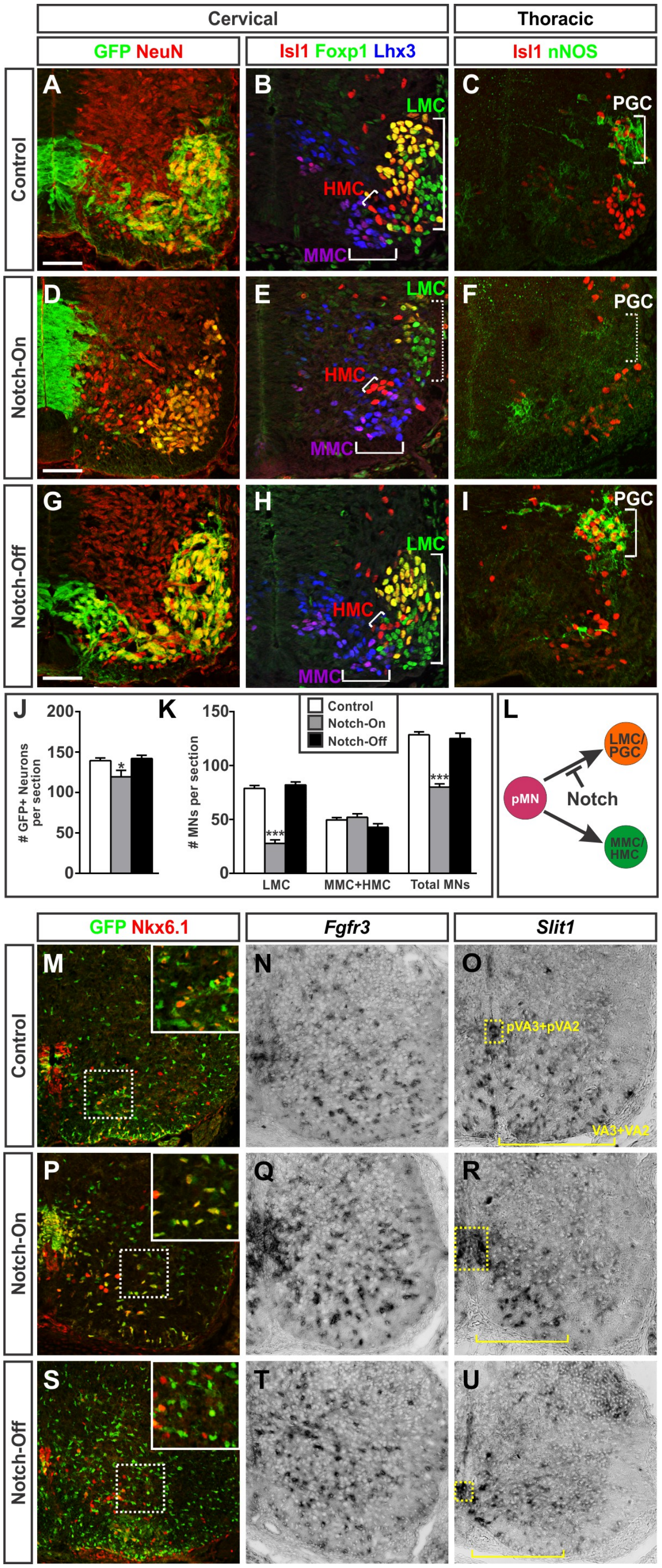

(A-I) In E11.5 embryos, *Olig2*^{Cre}-derived neurons (GFP$^+$/NeuN$^+$) include LMC (FoxP1$^+$), HMC (Isl1$^+$/FoxP1$^-$/Lhx3$^-$), MMC (Isl1$^+$/FoxP1$^-$/Lhx3$^+$), and PGC (Isl1$^+$/nNOS$^+$) MNs. Scale bars = 50 µm.
(J) Quantification of the total number of recombined neurons (GFP$^+$/NeuN$^+$) per spinal cord half. Plots show the mean ± SEM from multiple sections collected from 11-13 embryos from each experimental condition. NS, $p > 0.05$ and *$p < 0.05$.
(K) Quantification of the total number of MNs (Isl1$^+$ and/or Hb9$^+$), LMC MNs (FoxP1$^+$), and MMC plus HMC MNs (Foxp1$^-$ Isl1$^+$ Lhx3$^+$ and Foxp1$^-$ Isl1$^+$ Lhx3$^-$) per spinal cord half. In this analysis, all MNs were counted regardless of their GFP expression. Plots show the mean ± SEM from multiple sections collected from 8-30 embryos from each experimental condition. NS, $p > 0.05$ and ***$p < 0.001$.
(L) Summary of the role of Notch signaling in directing MN cell fates.
(M-U) Analysis of the impact of Notch manipulations on glial cell fates at E18.5.
(M-O) In Control embryos, *Olig2*^{Cre}-derivatives marked by GFP expression include pVA3 astrocyte progenitors and differentiated VA3 astrocytes that express Nkx6.1 (Hochstim et al, 2008). Serial section analysis reveals the presence of *Fgfr3*, which is expressed by multiple groups of astrocyte progenitors, and *Slit1*, which is selectively expressed by VA3 progenitors and differentiated astrocytes (Hochstim et al, 2008).
(P-U) Notch activation increases while Notch inactivation decreases the production of cells expressing the pVA3 and VA3 markers Nkx6.1, *Fgfr3*, and *Slit1*.

Figure S4. Manipulation of Notch signaling alters neuronal differentiation and progenitor maintenance in the intermediate spinal cord without overt changes in dorsoventral patterning or glial identities (related to Figures 2 and 3)

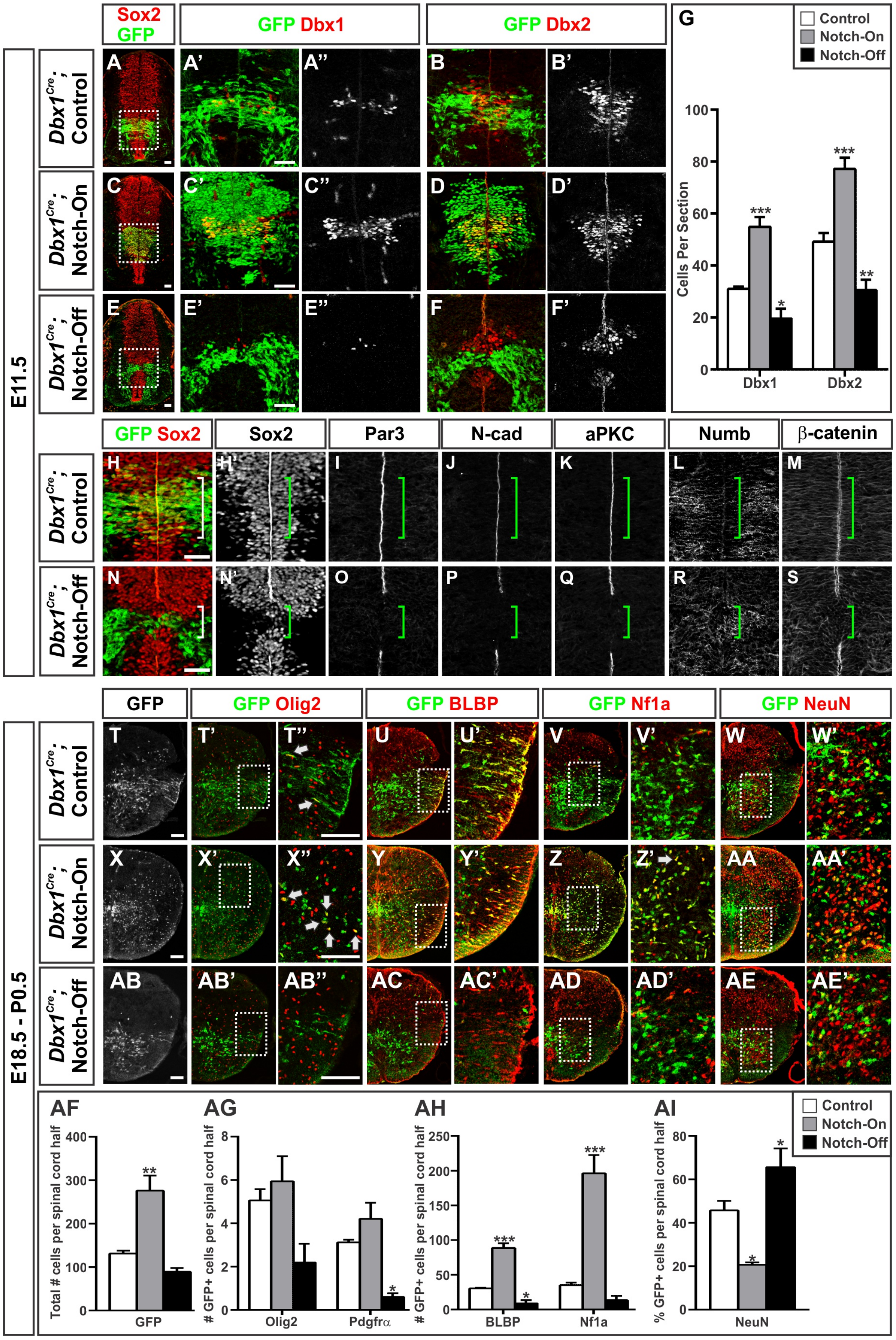

(A-F) In E11.5 control embryos, *Dbx1*^{Cre}-mediated recombination occurs within p0 (Dbx1^{+} Dbx2^{+}) and p1 (Dbx1^{-} Dbx2^{+}) progenitors. Notch activation increases while Notch inactivation decreases the formation of these cells. Scale bars = 50 μm.
(G) Quantification of the total number of Dbx1^{+} and Dbx2^{+} progenitors present in E11.5 Control, Notch-On, and Notch-Off embryos. For this analysis, progenitors were counted regardless of their GFP expression. Plots represent mean cell counts ± SEM from multiple sections collected from 6-8 embryos from each transgenic line. $^{*}p < 0.05$, $^{**}p < 0.01$, $^{***}p < 0.001$.
(H-S) The neuroepithelial architecture and apicobasal polarity of neural progenitors is greatly disrupted in the intermediate spinal cord of E11.5 *Dbx1*^{Cre}; Notch-Off embryos (N-S). Within the region of recombination, this disruption can be observed through the complete loss of Sox2^{+} progenitors, cell polarity components (Par3 and aPKC), and cell-to-cell adhesion molecules (N-cadherin and β-catenin). Green brackets demarcate the region of recombination. Scale bars = 100 μm.
(T-AE) In E18.5 Control embryos and P0.5 neonates, GFP^{+} Dbx1^{Cre}-derivatives include astrocyte precursors and astrocytes (BLBP^{+}/Nf1a^{+}), differentiated neurons (NeuN^{+}), but only a small number of oligodendrocyte precursors (OLPs) (Olig2^{+} or Pdgfrα^{+}). Notch activation increases the formation of astrocyte progenitors and astrocytes while at the same time reducing neurogenesis. Notch inactivation has the opposite effect. In contrast to *Olig2*$^{Cre-}$mediated manipulations, *Dbx1*^{Cre}-mediated Notch activation does not show any suppressive effect on oligodendrocyte formation. Likewise, *Dbx1*^{Cre}-mediated Notch inactivation does not lead to the formation of ectopic oligodendrocyte progenitors. Scale bars = 100 μm.
(AF-AI) Quantification of the total number of GFP^{+} cells per spinal cord half, GFP^{+} OLPs (GFP^{+}/Olig2^{+} and GFP^{+}/Pdgfrα^{+}), astrocyte precursors and astrocytes (GFP^{+}/BLBP^{+} and GFP^{+}/Nf1a^{+}), and neurons (GFP^{+}/NeuN^{+}). Plots display the mean ± SEM from multiple sections collected from 4-5 embryos for each experimental condition. $^{*}p < 0.05$, $^{**}p < 0.01$, $^{***}p < 0.001$.

Figure S5. Inhibition of Notch signaling reduces Smo trafficking to the primary cilia of NIH-3T3 fibroblasts with no disruption to apicobasal polarity (related to Figure 5).

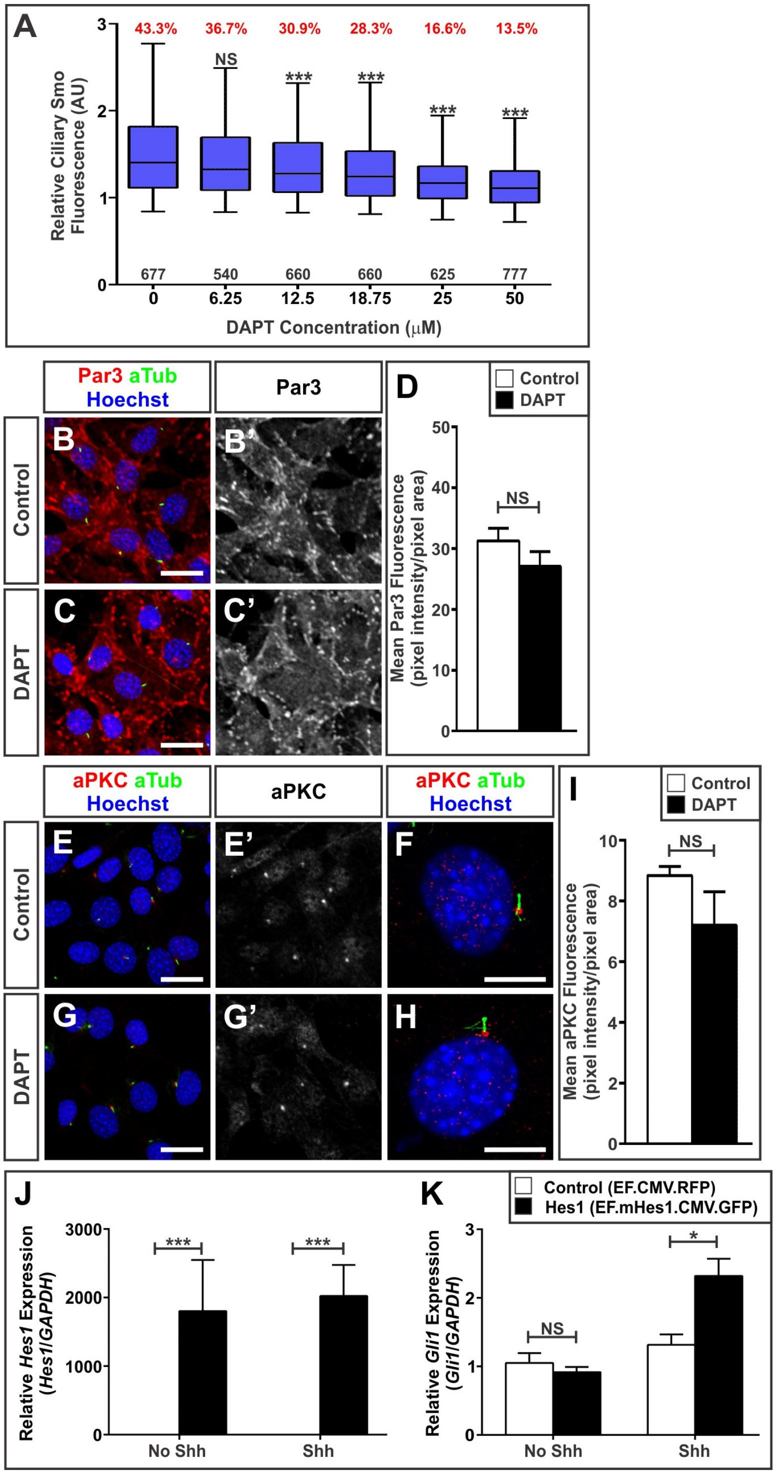

(A) Box and whisker plots of Smo fluorescence in the cilia of NIH-3T3 cells cultured in the presence of 50 nM Shh and various doses of DAPT (0-50 μM). The box extends from the 25th to 75th percentile, the line through the box represents the median, and the whiskers encompass

the 5th to 95th percentile. The number of cilia analyzed is indicated in black below the box plots and the percentage of Smo^{+} cilia indicated in red above the box plots. DAPT reduces Smo presence within the primary cilia in a dose-dependent manner. For statistical analyses, all DAPT doses were compared to the Control (DMSO) group. NS, $p > 0.05$ and *** $p < 0.001$.
(B-C) Par3 immunofluorescence analysis of Control (DMSO) and DAPT (18.75 μM) treated NIH-3T3 cells. Scale bars = 20 μm.
(D) Quantification of the mean intensity ± SEM of Par3 fluorescence in a 320 μm x 320 μm area.
(E-H) aPKC immunofluorescence analysis of Control (DMSO) and DAPT (18.75 μM) treated NIH-3T3 cells. Low mag scale bars = 20 μm. High mag scale bars = 10 μm.
(I) Quantification of the mean intensity ± SEM of aPKC fluorescence in a 320 μm x 320 μm area.
(J-K) qPCR analysis of *Hes1* and *Gli1* mRNA in NIH-3T3 cells transiently transfected with either EF.CMV.RFP or EF.mHes1.CMV.GFP expression vectors and then cultured in the presence or absence of Shh for 12 hr. Plots represent mean *Gapdh*-normalized expression levels relative to EF.CMV.RFP controls ± SEM from 4-6 samples for each condition.

Figure S6. Inhibition of Notch signaling reduces Smo trafficking to primary cilia in a range of cell types (related to Figure 5).

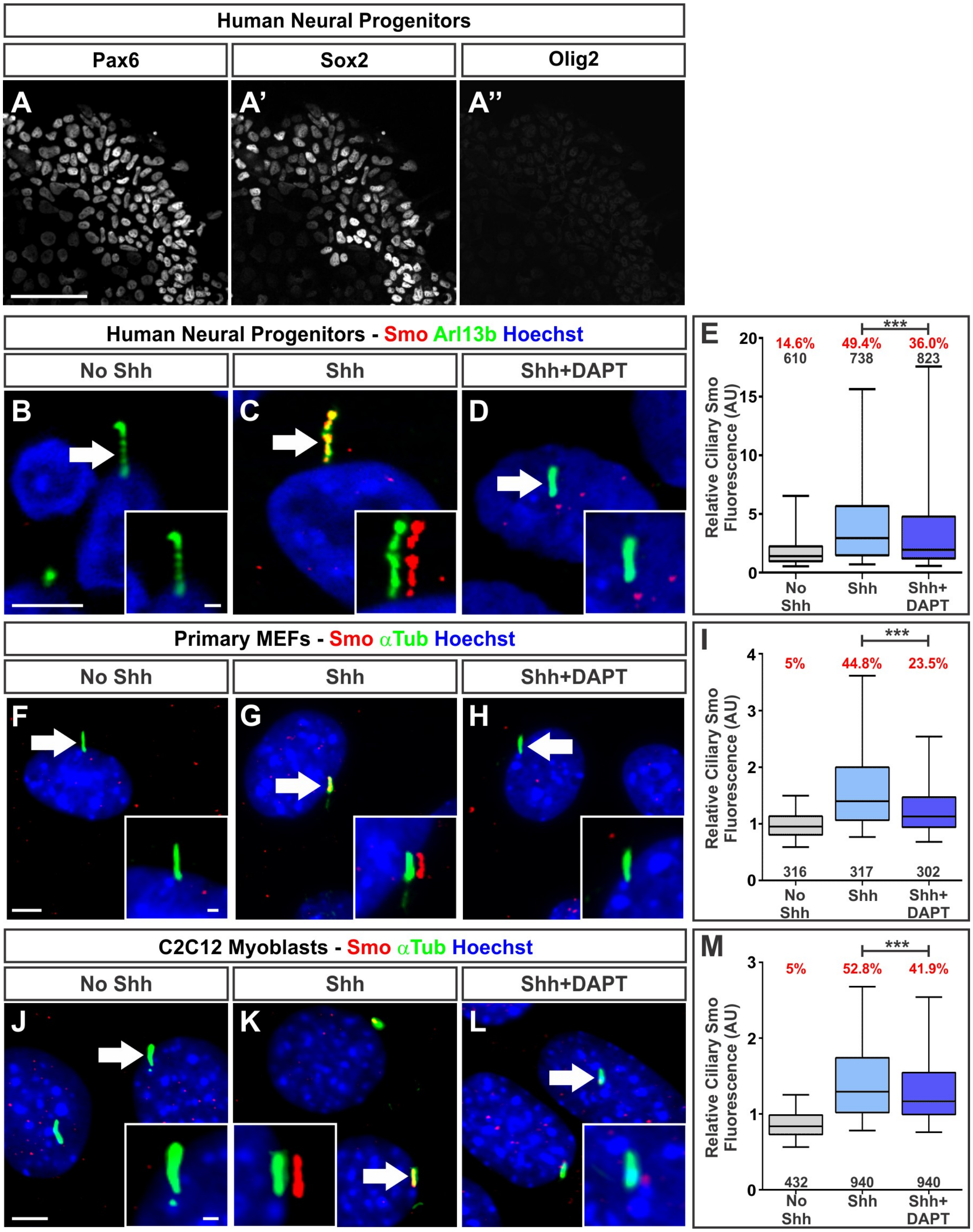

(A-A'') Immunofluorescence analysis of day 11 human spinal neural progenitors generated from the directed differentiation of H9 human embryonic stem cells. The majority of these cells express canonical neural progenitor markers such as SOX2 and PAX6, but not OLIG2 prior to the addition of Shh. Scale bars = 100 μm.

(B-M) Human neural progenitors (B-D), primary mouse embryonic fibroblasts (MEFs) (F-H), and mouse C2C12 skeletal myoblasts (J-L) exposed to 50 nM Shh with or without DAPT for 12 hr. Plated cells were immunostained for Smo (red), Arl13b or αTubulin (green, primary cilia), and Hoechst (blue). Arrows denote cilia shown in the insets, in which Smo and Arl13b/αTubulin channels are offset to better show colocalization. Low mag scale bars = 5 μm. High mag scale bars in insets = 1 μm.
(E, I, M) Box and whisker plots of Smo fluorescence in the cilia of various cell types. The box represents the 25th to 75th percentile, the line through the box represents the median, and the whiskers encompass the 5th to 95th percentile. The number of cilia analyzed is indicated in black and the percentage of Smo^{+} cilia indicated in red. ***$p < 0.001$.

Figure S7. Manipulating neural progenitor identities in a manner independent of Notch signaling does not alter Smo accumulation within primary cilia (related to Figure 6).

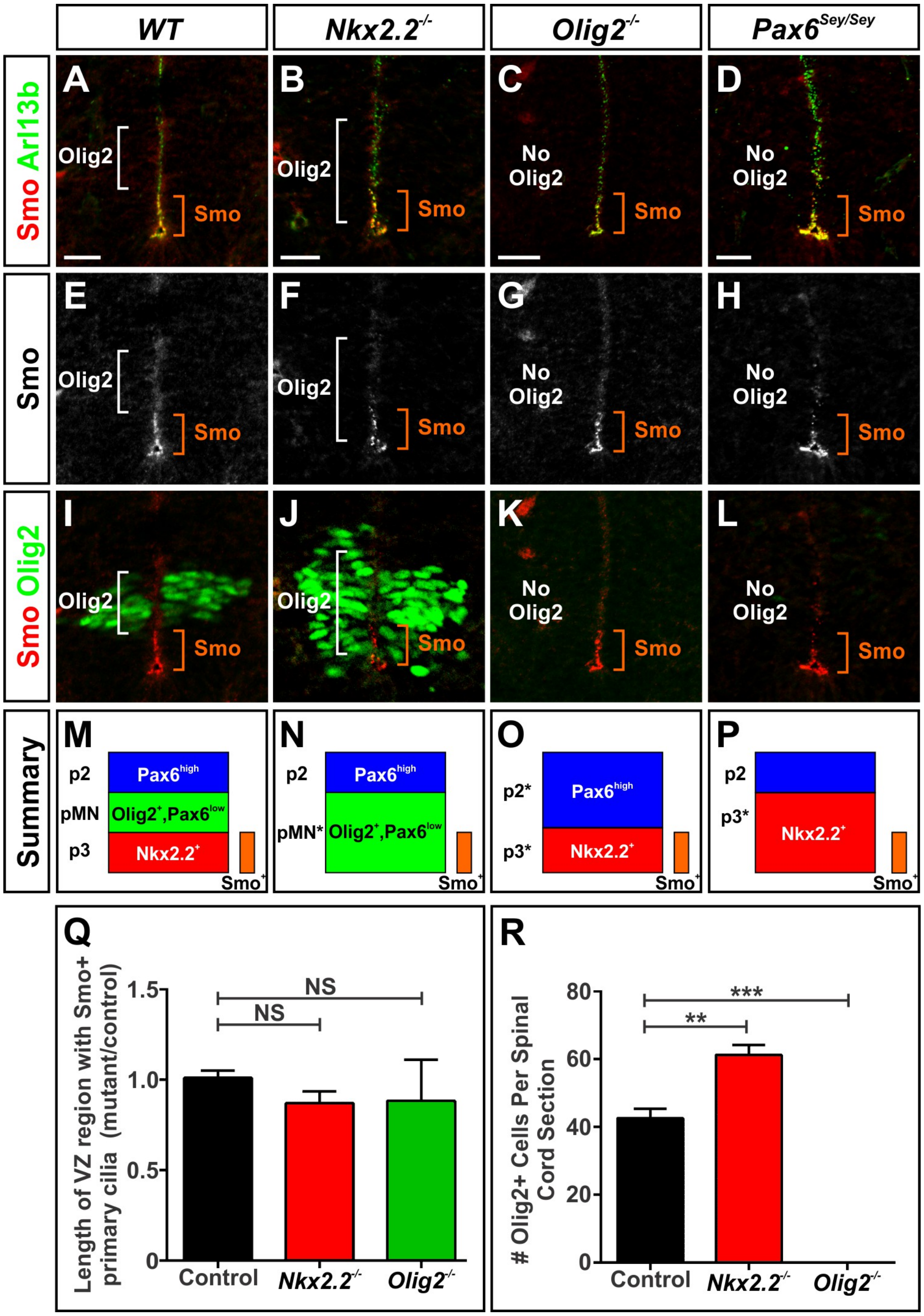

(A-L) Analysis of Smo^{+} primary cilia present on ventral spinal cord progenitors in E11.5 *WT* (A, E, I), *Nkx2.2*$^{-/-}$ (B, F, J), *Olig2*$^{-/-}$ (C, G, K), and *Pax6*$^{Sey/Sey}$ (D, H, L) mutants. Tissues were stained with antibodies against Smo, Arl13b, and Olig2. White brackets denote the limits of the $Olig2^{+}$/pMN progenitor domain and the orange brackets illustrate the dorsoventral extent of progenitor cells with Smo^{+} primary cilia. All scale bars = 20 µm.
(M-P) Summary of the mutant spinal cord data.

(Q) Quantification of the dorsoventral limits of Smo^+ primary cilia in *WT*, *Nkx2.2*$^{-/-}$, and *Olig2*$^{-/-}$ mutants. Plots represent the mean length ± SEM of Smo^+ cilia normalized against littermate controls. For this analysis, multiple sections were imaged from at least 3 embryos from each experimental group. NS, $p > 0.05$.

(R) Quantification of the number of $Olig2^+$ cells per spinal cord. Plots represent the mean ± SEM from multiple sections collected from 3-4 embryos from each group. $^{**}p < 0.01$ and $^{***}p < 0.001$.

Figure S8. DAPT Inhibition of Smo translocation to the primary cilia requires at least 12 hr exposure and is dependent on Ptch1 function (related to Figure 7).

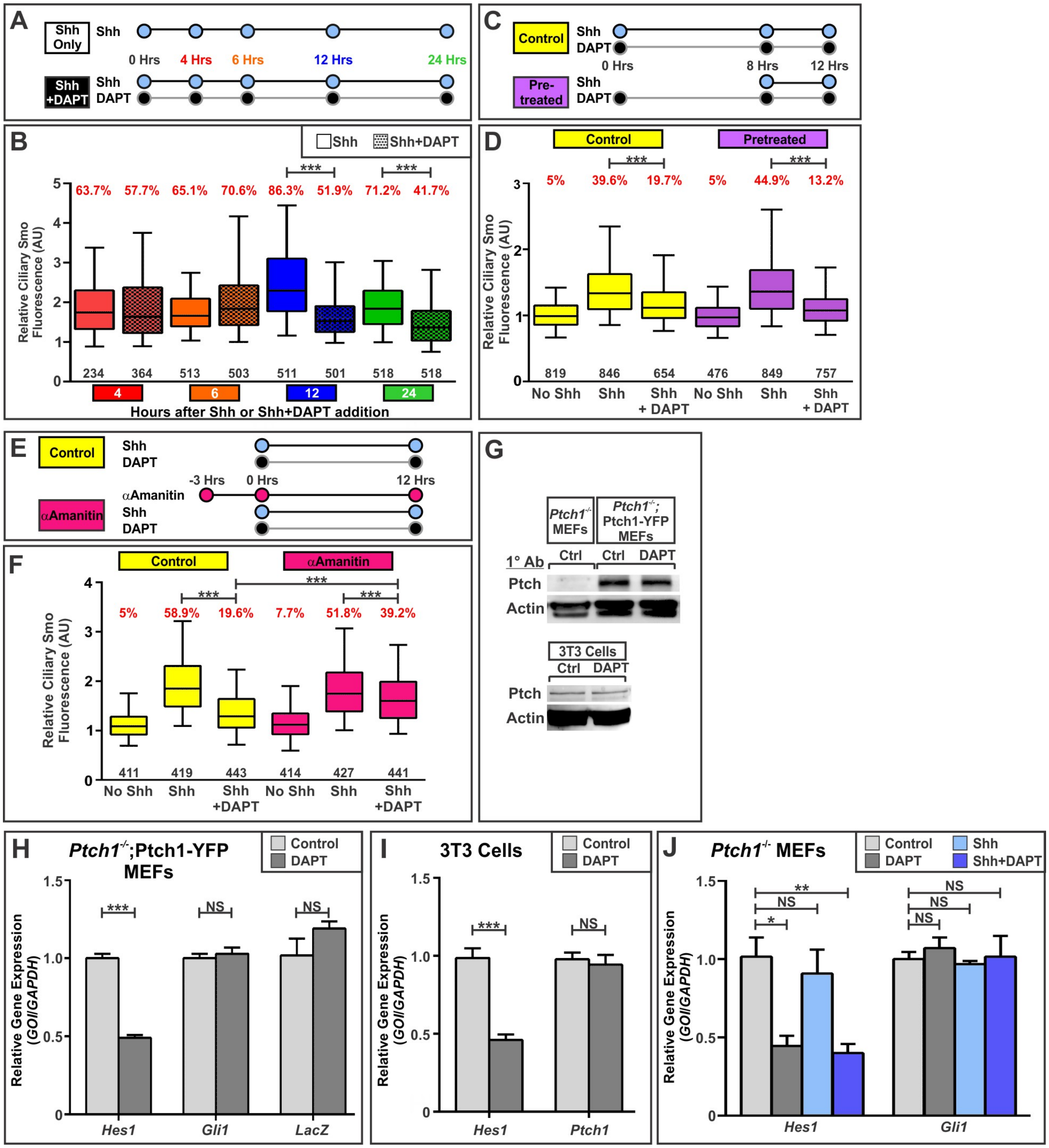

(A) Outline of the time course experiments in which NIH-3T3 cells were exposed to 50 nM Shh alone or in the presence of 18.75 µM DAPT for 4, 6, 12, and 24 hr.

(B) Box and whisker plots of Smo fluorescence in the cilia of cells treated with Shh ± DAPT for 4, 6, 12, and 24 hr. The box represents the 25th to 75th percentile, the line through the box represents the median, and the whiskers encompass the 5th to 95th percentile. The number of cilia analyzed is indicated in black below the box plots, and percentage of Smo^{+} cilia indicated in

red above the box plots. Reductions in Smo^+ primary cilia were first observed at 12 hr. ***p < 0.001.

(C) Outline of the pretreatment experiment, in which NIH-3T3 cells were exposed to either Shh+DAPT for 12 hr (Control) or exposed to DAPT alone for 8 hr and then Shh+DAPT for an additional 4 hr (Pretreatment).

(D) Box and whisker plots of ciliary Smo fluorescence under Control and DAPT Pretreated conditions. ***p < 0.001.

(E) Outline of the α-amanitin experiment. NIH-3T3 cells were exposed to the transcriptional inhibitor α-amanitin 3 hr prior to the addition of Shh with or without DAPT.

(F) Box and whisker plots of ciliary Smo fluorescence in the presence or absence of α-amanitin. ***p < 0.001.

(G) Immunoblotting for Ptch1 and Actin in cell lysates of *Ptch1*$^{-/-}$ MEFs, *Ptch1*$^{-/-}$*; Ptch1-YFP* MEFs, and NIH-3T3 cells treated with either DMSO (Control) or DAPT for 24 hr.

(H) qPCR analysis of *Ptch1*$^{-/-}$*;Ptch1-YFP* MEFs treated with either DMSO (Control) or DAPT for 12 hr. Plot shows mean *Gapdh*-normalized gene expression levels relative to the Control group ± SEM from 4 samples. NS, p > 0.05, ***p < 0.001, unpaired t-test.

(I) qPCR analysis of NIH-3T3 cells treated with DMSO (Control) or DAPT for 24 hr. Plot represents mean *Gapdh*-normalized gene expression levels relative to the control group ± SEM from 3 samples. NS, p > 0.05, *p < 0.05, **p < 0.01, ***p < 0.001.

(J) qPCR analysis of *Ptch1*$^{-/-}$ MEFs treated with DMSO (Control), DAPT, Shh, or Shh+DAPT for 24 hr. Plot represents mean *Gapdh*-normalized gene expression levels relative to the Control group ± SEM from 3 samples. NS, p > 0.05, *p < 0.05, **p < 0.01, ***p < 0.001.

SUPPLEMENTAL EXPERIMENTAL PROCEDURES

Antibodies and in situ hybridization probes

Primary antibodies used for immunohistochemistry were as follows: mouse anti-Arl13b (NeuroMab 75-287), 1:100; mouse anti-Ascl1 (Lo et al., 1991), 1:200; rabbit anti-BLBP (Chemicon/Millipore AB9558), 1:2,000; rabbit anti-β-Catenin (Sigma C2206), 1:1,000; guinea pig anti-Chx10 (Vsx2) (Thaler et al., 1999), 1:5,000; mouse anti-Cre (Covance MMS-106P), 1:2,000; rabbit anti-Dbx1 (Vue et al., 2007), 1:1,000; rabbit anti-Dbx2 (Abcam ab25554), 1:4,000; mouse anti-Foxa2 (Developmental Studies Hybridoma Bank 4C7), 1:200; rabbit anti-Foxa2 (Weinstein et al., 1994), 1:4,000; guinea pig anti-Foxp1 (Rousso et al., 2008), 1:16,000; goat anti-Gata3 (Santa Cruz Biotechnology sc-1236), 1:200; chicken anti-GFP (Aves Labs GFP-1020), 1:1,000; rabbit anti-GFP (Invitrogen A6455), 1:4000; sheep anti-GFP (AbD Serotec 4745-1051), 1:800; rabbit anti-Hb9 (Mnx1) (Arber et al., 1999), 1:8000; rabbit anti-Hes1 (Ito et al., 2000), 1:2,000; rabbit anti-Irx3 (Novitch et al., 2003), 1:8,000; goat anti-Islet1 (R&D Systems AF1837), 1:8,000; rabbit anti-Islet1/2 (Tsuchida et al., 1994), 1:20,000; rabbit anti-Lhx3 (Ericson et al., 1997), 1:4,000; rat anti-N-cadherin (Developmental Studies Hybridoma Bank MNCD2), 1:50; rabbit anti-N-cadherin (Abcam ab12221), 1:1,000; mouse anti-NeuN (Millipore MAB377B), 1:2,000; goat anti-Neurog2 (Santa Cruz Biotechnology sc-19233), 1:1,000; rabbit anti-Nf1a (Kang et al., 2012), 1:3,000; mouse anti-Nkx2.2 (Developmental Studies Hybridoma Bank 74.545), 1:25; rabbit anti-Nkx2.2 (Briscoe et al., 1999), 1:10,000; guinea pig anti-Nkx6.1 (Briscoe et al., 2000), 1:4,000; mouse anti-Nkx6.1 (Developmental Studies Hybridoma Bank F55A10), 1:25; rabbit anti-nNos (Immunostar 24287), 1:10,000; rabbit anti-Cleaved-Notch1 (Cell Signaling Technology 2421), 1:500; rabbit anti-Numb (Abcam ab14140), 1:4,000; rabbit anti- Olig2 (chick) (Novitch et al., 2001), 1:8,000; guinea pig anti-Olig2 (mouse) (Novitch et al., 2003), 1:20,000; rabbit anti-Olig2 (mouse) (Millipore AB9610), 1:5,000; rabbit anti-Par3 (Millipore 07-330), 1:100; mouse anti-Pax6 (Developmental Studies Hybridoma Bank), 1:100; rabbit anti-Pax6, (Covance), 1:4,000; rat anti-Pdgfrα (eBiosciences 14-1401), 1:1,000; rabbit anti-aPKC (PKCζ), (Santa Cruz Biotechnology SC-216), 1:1600; rabbit anti-Ptch1 (Rohatgi et al., 2007), 1:500; rat anti-Rbpj (Active Motif 61506), 1:100; mouse anti-Shh (Developmental Studies Hybridoma Bank 5E1), 1:100; rabbit anti-Smo (Abcam ab38686), 1:3,000; rabbit anti-Smo (Rohatgi et al., 2007), 1:500; goat anti-Sox2 (Santa Cruz Biotechnology sc-17320), 1:2,000; goat anti-Sox10 (R&D Systems AF2864), 1:300; mouse anti-αTubulin (Sigma T6793), 1:1,000; rabbit anti-βIII-Tubulin (TUJ1) (Covance MRB-435P), 1:5,000. Alexa^{488}-, FITC-, Cy3-, Cy5-, and Dylight^{649}-conjugated secondary antibodies were obtained from Jackson Immunoresearch Laboratories, Inc. (West Grove, PA). RNA probes were generated by in vitro transcription of PCR products amplified from mouse spinal cord cDNA. Primers against the following genes were designed using the program Primer3 (http://bioinfo.ut.ee/primer3/). *Hes5*, forward 5'-GGATGAGCTCGTTCCTCTGG-3' and reverse 5'-GAGATTAACCCTCACTAAAGGGAGCAGGCTGAGTGCTTTCCTA-3' and *Neurog3*, forward 5'-AGGCTTCTCATCGGTACCCT-3' and reverse 5'-GAGATTAACCCTCACTAAAGGGCATAGGCTAGGGCTTTCCGG-3'. The underlined text denotes a T3 polymerase binding site incorporated into the reverse primer. Probes for *Fgfr3* (Gaber et al., 2013), *Slit1* (Hochstim et al., 2008), *Gli1* and *Ptch1* (Ribes et al., 2010) were used as previously described. All probes were generated using a Digoxigenin (DIG) RNA Labeling Kit (Roche) and visualized using a combination of Anti-DIG-alkaline phosphatase (AP) Fab fragments (Roche) and NBT/BCIP (Roche).

Chick neural explant culture

Neural tissue was isolated from chick embryos (HH stage 10) and cultured as previously described (Yamada et al., 1993; Dessaud et al., 2007). Two types of explants were prepared: intermediate spinal cord explants (i) and ventral neural plate plus floor plate explants (vf). Shh

was generated as previously described (Ericson et al., 1996) and the concentration of each new batch determined by comparison with previous batches. NKX2.2 and OLIG2 quantifications consist of data collected from 7-10 explants per condition. Plotted data represents the mean ± SEM.

Quantitative PCR

qPCR was carried out as previously described (Rousso et al., 2012). Briefly, total RNA was extracted using the RNeasy Mini Kit (Qiagen). For each sample, ~500-1000 ng of total RNA was used to synthesize cDNA by reverse transcription using the SuperScript III First-Strand Synthesis System (Invitrogen). In each qPCR reaction, cDNA was combined with LightCycler 480 SYBR Green I Master Mix (Roche) and the following exon-spanning primer pairs: mouse *Gli1*, forward 5'-CACCGTGGGAGTAAACAGGCCTTCC-3' and reverse 5'-CCAGAGCGTTACACACCTGCCCTTC-3' (Regl et al., 2002); mouse *Gli1*, forward 5'-ATCTCTCTTTCCTCCTCCTCC-3' and reverse 5'-CGAGGCTGGCATCAGAA-3' (Lelievre et al., 2008); mouse *Patched1*, forward 5'-CTGCCTGTCCTCTTATCCTTC-3' and reverse 5'-AGACCCATTGTTCGTGTGAC-3'; mouse *Sox2*, forward 5'-CACATGGCCCAGCACTAC-3' and reverse 5'-CCCTCCCAATTCCCTTGTATC-3'; mouse *Gapdh*, forward 5′–GGCCTTCCGTGTTCCTAC–3′ and reverse 5′–TGTCATCATACTTGGCAGGTT–3' (Lelievre et al., 2008); mouse *Hes1*, forward 5'-GGCGAAGGGCAAGAATAAATG-3' and reverse 5'-GTGCTTCACAGTCATTTCCAG-3'. The 18 to 20-mer primers were either chosen from papers as cited or designed using the IDT Primer Quest Program (http://www.idtdna.com/primerquest/Home/Index). All primer pairs were experimentally validated using E10.5 whole spinal cord cDNA. In this validation process each pair was shown to have amplification efficiency between 1.8-2.2 and possess a single gene-specific product using melting curve analysis. All samples were run using a Roche LightCycler 480 real-time PCR system in duplicates or triplicates, and relative mRNA expression levels determined by normalizing the crossing points of each gene of interest to *Gapdh*. Unless otherwise indicated relative gene expression profiles were plotted by comparison to the average value of control samples, set to 1.0.

Cell Culture

Reagents: Shh (Akron Biotech) was reconstituted in 0.1% BSA, DAPT and Purmorphamine (Calbiochem) in DMSO, and SAG (Calbiochem) in water. To account for these different vehicles, all control samples were treated with equivalent volumes of DMSO and BSA to experimental samples. The concentrations of Shh, Pur, SAG, and DAPT used were empirically determined by exposing fibroblast cells to a range of doses and assessing Shh and Notch signaling activity using qPCR and luciferase assays. Across batches, Shh (50 nM), Pur (5 µM), and SAG (1 µM) consistently activated Shh signaling. However, there were some potency differences between batches of DAPT (5-25 µM). To account for these differences, we optimized the concentration of each lot of DAPT using qPCR measurement of *Hes1* mRNA to readout Notch signaling activity. In each experiment, the amount of DAPT used was found to reduce *Hes1* expression by at least 50%.

NIH-3T3 cells and primary MEFs: NIH-3T3 cells were cultured in high glucose (4.5 g/L glucose) Dulbecco's modified Eagle's medium (DMEM) with 110 mg/L sodium pyruvate (Gibco), 10% bovine calf serum (BCS, Gibco), 1x glutamax (Gibco), 1% Penicillin:Streptomycin (Gibco), and 0.2% Primocin (InvivoGen). Shh-LIGHT2 cells were cultured in high glucose (4.5 g/L glucose) DMEM with 110 mg/L sodium pyruvate (Gibco), 10% BCS (Gibco), 0.15 mg/ml zeocin (Invitrogen), and 0.4 mg/ml geneticin (Gibco), as previously described (Taipale et al., 2000). Wild-type, *Ptch1*$^{-/-}$*, and Ptch1*$^{-/-}$;Ptch1-YFP mouse embryo fibroblasts were cultured as previously described (Rohatgi et al., 2007). For Shh induction experiments, cells were plated onto 8 mm coverslips in 24-well plates (40,000 cells/well) in regular (10% BCS) growth media.

Upon reaching 80-100% confluency, cells were moved into low serum media (0.5% BCS) supplemented with one or more of the following: Shh ligand (50 nM, Akron Biotech), Purmorphamine (5 µM, Calbiochem), SAG (1 µM, Calbiochem), DAPT (18.75 µM, Calbiochem), SAHM (20 µM, Calbiochem), and JLK6 (20 µM, Calbiochem).
C2C12 myoblasts: C2C12 cells were plated directly onto 8 mm coverslips in a 24-well plate (40,000 cells/well) in DMEM with L-glutamine (Gibco) supplemented with 10% FBS. Upon reaching 80-100% confluency, the cells were switched to a low serum media (0.5% FBS) and treated with Shh ± DAPT for 12 hr.
Human neural progenitors: Primitive human neuroepithelial progenitors were generated from embryonic stem cells as previously described (Hu and Zhang, 2009). After 10 days of culture in vitro, neural rosettes were manually picked and plated onto poly-ornithine/laminin coated coverslips in DMEM/F12 (Hyclone), 1x N2 (Life Technologies), 0.1mM NEAA (Life Technologies), 1 mg/mL heparin (Sigma), and 10% FBS (Hyclone), and allowed to attach for 24 hr. The following day, FBS was removed and the neural rosettes were exposed to Shh ± DAPT for 12 hr.

Transient transfection of NIH-3T3 cells
NIH-3T3 cells were transiently transfected with either pCIG, pCIG-NICD, EF.CMV.RFP (Addgene plasmid # 17619), or EF.mHes1.CMV.GFP (Addgene plasmid # 17622) constructs (Megason and McMahon, 2002; Yu et al., 2003; Yu et al., 2006) using the transfection reagent FuGENE6 (Roche) at a DNA:FuGENE6 ratio of 1:3.

Luciferase assays
In chick explants: Gli activity was measured in chick neural explants as previously described (Dessaud et al., 2007). Briefly, chick embryos (HH stage 10) were coelectroporated with a Gli-binding site firefly luciferase reporter and a cytomegalovirus promoter::renilla luciferase plasmid (Promega) to normalize for transfection efficiency, returned to the incubator for 2 hr, and then collected for explant culture. Upon isolation, neural explants were exposed to Shh ± DAPT for 12 or 24 hr. Tissue was then homogenized on ice in Passive Lysis Buffer and processed using a Dual-Luciferase Reporter Assay System (Promega). Each data point represents the mean ± SEM from 7-10 explants.
In cells: All luciferase assays were conducted using Shh-LIGHT2 cells, NIH-3T3 cells that have been stably cotransfected with Gli-binding site::firefly luciferase and herpes simplex virus thymidine kinase (HSV TK) promoter::renilla luciferase plasmids (Taipale et al., 2000). Shh-LIGHT2 cells were cultured in 96-well plates (30,000 cells/well) in regular serum media (10% BCS). Upon reaching 80-100% confluency, the cells were moved into a low serum media (0.5% BCS) containing Shh ligand (0-50 nM, Akron Biotech) ± DAPT (0-50 µM, Calbiochem). After 24 hr, cells were rinsed in PBS and then lysed in Passive Lysis Buffer (100 µl/well, Promega Dual-Luciferase Reporter Assay System). Luciferase activities were measured in a Tecan M1000 microplate reader equipped with an automatic injector. For each sample, Gli-dependent firefly luciferase activity was normalized to HSV TK::renilla luciferase activity and the resulting ratio reported in relative luciferase units (RLU). All luciferase conditions were run as duplicate or triplicate samples. Each data point represents the mean ± SEM.

Tissue image analysis
Fluorescence and DIC images were collected using either a Zeiss LSM5 Exciter or LSM780 confocal imaging system or a Zeiss AxioImager M2 fluorescence microscope equipped with Apotome attachment and motorized stage. Images were collected and processed using LSM Exciter, Zeiss AxioVision, Zeiss Efficient Navigation (ZEN), and Adobe Photoshop software. Fluorescence intensity quantifications and cell number counts were performed using the NIH

developed image processing program ImageJ with an Image-based Tool for Counting Nuclei (ITCN) plugin. Composite images were assembled using CorelDRAW X7 software.

Analysis of Smo and Ptch1 presence in primary cilia

Fixed cells were imaged on an inverted Zeiss LSM 780 laser scanning confocal microscope or an upright Zeiss AxioImager M2 fluorescence microscope equipped with Apotome attachment and motorized stage. Images were taken with 20x, 40x oil, and 63x oil immersion objectives. For each experiment, coverslips from each condition were processed side by side to ensure that the cells were fixed and stained for the same duration of time. To ensure uniformity in imaging, the gain, offset, and laser power settings on the microscope were held constant for Smo and Ptch1. Quantification of relative ciliary Smo and Ptch1 fluorescence levels were performed as previously described (Mukhopadhyay et al., 2013; Nachtergaele et al., 2013), with minor modifications. Briefly, using the program ImageJ, an outline was first drawn around each cilium (labeled by α-acetylated tubulin or Arl13b staining), and the corresponding intensity of Smo or Ptch1 fluorescence within and adjacent to the cilium measured. From these measurements, a ratio of the intensity was then calculated for each cilium (ratio = intensity of fluorescence within the cilium ÷ fluorescence adjacent to the cilium). When this ratio (i.e. relative ciliary Smo or Ptch1 fluorescence) is close to 1, the intensity of Smo or Ptch1 fluorescence within the cilium is not above background levels. The relative fluorescence values within a primary cilium that visually appeared to be Smo or Ptch1 positive varied widely based on cell type. In NIH-3T3 cells, the relative Smo or Ptch1 fluorescence within a positive primary cilium was at least 1.3-1.5. In human neural progenitors, the relative Smo fluorescence within a positive primary cilium was at least 3. Smo and Ptch1 were measured in approximately 100-800 primary cilia per each experimental group. These 100-800 relative ciliary fluorescence values were then represented in a box and whisker graph. In each graph, the upper and lower limits of the box represent the 25th to 75th percentiles, the line in the center is the median, and the whiskers encompass the 5th to the 95th percentiles. In each graph, the black numbers present above or below the box plots are the number of primary cilia analyzed for each group and the red numbers are the percentage of Smo or Ptch1 positive cilia. Although all statistical evaluations were done using the relative ciliary fluorescence values, Smo or Ptch1 positive cilia percentages were included on each plot to distill the data into binary “positive” or “negative” cilia values. To determine if a primary cilium was Smo or Ptch1 positive, we established a “cut-off” ciliary fluorescence value unique to each experiment, as there was frequently variance in background staining values. We then grouped all cilia above the cut-off as positive and those below as negative. In most experiments, the cut-off was determined to be the value at which there were 5% Smo^+ or $Ptch1^+$ primary cilia in the negative control group (i.e. the group that possessed the fewest Smo or Ptch1-positive cilia). The negative control for Smo was the No Shh group and the negative control for Ptch1 was the Shh-only treated group. In some experiments, no true negative control group was present and in these cases the cut-off value was set to 1.5 for NIH-3T3 cells and 3.0 for human neural progenitors, based on the background staining values in surrounding regions away from the cilia.

Analysis of primary cilia lengths

Primary cilia of fixed cells were labeled using α-acetylated tubulin and Arl13b antibodies, and imaged on an inverted Zeiss LSM 780 laser scanning confocal microscope equipped with either a 63x or 100x oil immersion objective. To ensure the full length of each cilium was accurately measured, thin z-stacks were acquired and 3D surface reconstructions of the primary cilia generated using ZEN software (Carl Zeiss, Germany). All experimental cilia measurements were normalized against control cilia measurements. The values reported thus represent the mean changes in the percentage of cilia length ± SEM. For each condition, the lengths of over 100 cilia were individually analyzed. The reduction in primary cilia length observed with DAPT

addition and extension with NICD transfection was observed with both α-acetylated tubulin and Arl13b labeled cilia. Data was analyzed using an unpaired, two-tailed t-test. Significance was assumed when $p < 0.05$.

Immunoblotting

Cell cultures were scaled up to 100 mm plates and grown as described above. Cells were manually scraped, rinsed once in PBS, lysed in a modified RIPA buffer (1% NP40, 1% sodium deoxycholate, 0.3% SDS, 150 mM NaCl, 1 mM EDTA, 50 mM Tris pH6.8, 1 mM PMSF, 1x complete protease cocktail (Roche)) for 2 hr on ice with vortexing every 10 min, and clarified by centrifugation for 30 min at 14,000 RPM at 4ºC. Protein lysates were resolved on an 8-10% polyacrylamide gel and processed for immunoblotting with the following antibodies: Rabbit anti-Notch1 (Val1744, Cell Signaling Technologies), 1:1000; rabbit anti-Ptch1 (Rohatgi Lab), 1:1000; mouse anti-actin (Millipore), 1:1000, followed by HRP-conjugated anti-mouse and anti-rabbit secondary antibodies (Jackson Immunoresearch), 1:50,000. Membranes were processed using ECL 2 Western Blotting Substrates (Thermo Scientific) and bands detected using a Typhoon FLA 7000 imaging system (GE Healthcare Life Sciences).

SUPPLEMENTAL REFERENCES

Arber, S., Han, B., Mendelsohn, M., Smith, M., Jessell, T.M., and Sockanathan, S. (1999). Requirement for the homeobox gene Hb9 in the consolidation of motor neuron identity. Neuron *23*, 659-674.

Briscoe, J., Sussel, L., Serup, P., Hartigan-O'Connor, D., Jessell, T.M., Rubenstein, J.L., and Ericson, J. (1999). Homeobox gene Nkx2.2 and specification of neuronal identity by graded Sonic hedgehog signalling. Nature *398*, 622-627.

Ericson, J., Rashbass, P., Schedl, A., Brenner-Morton, S., Kawakami, A., van Heyningen, V., Jessell, T.M., and Briscoe, J. (1997). Pax6 controls progenitor cell identity and neuronal fate in response to graded Shh signaling. Cell *90*, 169-180.

Ito, T., Udaka, N., Yazawa, T., Okudela, K., Hayashi, H., Sudo, T., Guillemot, F., Kageyama, R., and Kitamura, H. (2000). Basic helix-loop-helix transcription factors regulate the neuroendocrine differentiation of fetal mouse pulmonary epithelium. Development *127*, 3913-3921.

Kang, P., Lee, H.K., Glasgow, S.M., Finley, M., Donti, T., Gaber, Z.B., Graham, B.H., Foster, A.E., Novitch, B.G., Gronostajski, R.M., *et al.* (2012). Sox9 and NFIA coordinate a transcriptional regulatory cascade during the initiation of gliogenesis. Neuron *74*, 79-94.

Lelievre, V., Seksenyan, A., Nobuta, H., Yong, W.H., Chhith, S., Niewiadomski, P., Cohen, J.R., Dong, H., Flores, A., Liau, L.M., *et al.* (2008). Disruption of the PACAP gene promotes medulloblastoma in ptc1 mutant mice. Dev Biol *313*, 359-370.

Lo, L.C., Johnson, J.E., Wuenschell, C.W., Saito, T., and Anderson, D.J. (1991). Mammalian achaete-scute homolog 1 is transiently expressed by spatially restricted subsets of early neuroepithelial and neural crest cells. Genes Dev *5*, 1524-1537.

Megason, S.G., and McMahon, A.P. (2002). A mitogen gradient of dorsal midline Wnts organizes growth in the CNS. Development *129*, 2087-2098.

Mukhopadhyay, S., Wen, X., Ratti, N., Loktev, A., Rangell, L., Scales, S.J., and Jackson, P.K. (2013). The ciliary G-protein-coupled receptor Gpr161 negatively regulates the Sonic hedgehog pathway via cAMP signaling. Cell *152*, 210-223.

Nachtergaele, S., Whalen, D.M., Mydock, L.K., Zhao, Z., Malinauskas, T., Krishnan, K., Ingham, P.W., Covey, D.F., Siebold, C., and Rohatgi, R. (2013). Structure and function of the Smoothened extracellular domain in vertebrate Hedgehog signaling. Elife *2*, e01340.

Novitch, B.G., Wichterle, H., Jessell, T.M., and Sockanathan, S. (2003). A requirement for retinoic acid-mediated transcriptional activation in ventral neural patterning and motor neuron specification. Neuron *40*, 81-95.

Regl, G., Neill, G.W., Eichberger, T., Kasper, M., Ikram, M.S., Koller, J., Hintner, H., Quinn, A.G., Frischauf, A.M., and Aberger, F. (2002). Human GLI2 and GLI1 are part of a positive feedback mechanism in Basal Cell Carcinoma. Oncogene *21*, 5529-5539.

Ribes, V., Balaskas, N., Sasai, N., Cruz, C., Dessaud, E., Cayuso, J., Tozer, S., Yang, L.L., Novitch, B., Marti, E., *et al.* (2010). Distinct Sonic Hedgehog signaling dynamics specify floor plate and ventral neuronal progenitors in the vertebrate neural tube. Genes Dev *24*, 1186-1200.

Thaler, J., Harrison, K., Sharma, K., Lettieri, K., Kehrl, J., and Pfaff, S.L. (1999). Active suppression of interneuron programs within developing motor neurons revealed by analysis of homeodomain factor HB9. Neuron *23*, 675-687.

Vue, T.Y., Aaker, J., Taniguchi, A., Kazemzadeh, C., Skidmore, J.M., Martin, D.M., Martin, J.F., Treier, M., and Nakagawa, Y. (2007). Characterization of progenitor domains in the developing mouse thalamus. J Comp Neurol *505*, 73-91.

Weinstein, D.C., Ruiz i Altaba, A., Chen, W.S., Hoodless, P., Prezioso, V.R., Jessell, T.M., and Darnell, J.E., Jr. (1994). The winged-helix transcription factor HNF-3 beta is required for notochord development in the mouse embryo. Cell *78*, 575-588.

Yamada, T., Pfaff, S.L., Edlund, T., and Jessell, T.M. (1993). Control of cell pattern in the neural tube: motor neuron induction by diffusible factors from notochord and floor plate. Cell *73*, 673-686.

Yu, X., Alder, J.K., Chun, J.H., Friedman, A.D., Heimfeld, S., Cheng, L., and Civin, C.I. (2006). HES1 inhibits cycling of hematopoietic progenitor cells via DNA binding. Stem Cells *24*, 876-888.

Yu, X., Zhan, X., D'Costa, J., Tanavde, V.M., Ye, Z., Peng, T., Malehorn, M.T., Yang, X., Civin, C.I., and Cheng, L. (2003). Lentiviral vectors with two independent internal promoters transfer high-level expression of multiple transgenes to human hematopoietic stem-progenitor cells. Mol Ther *7*, 827-838.
