# Supplementary material for: Notch Activity Modulates the Responsiveness of Neural Progenitors to Sonic Hedgehog Signaling
Source: Dev Cell. 2015 May 26;33(4):373–87. doi: 10.1016/j.devcel.2015.03.005 (PMC4449290; doi:10.1016/j.devcel.2015.03.005)
Supplement: Document S2. Article plus Supplemental Information [file mmc2.pdf]

Developmental Cell

Article

Notch Activity Modulates the Responsiveness of Neural Progenitors to Sonic Hedgehog Signaling

Graphical Abstract

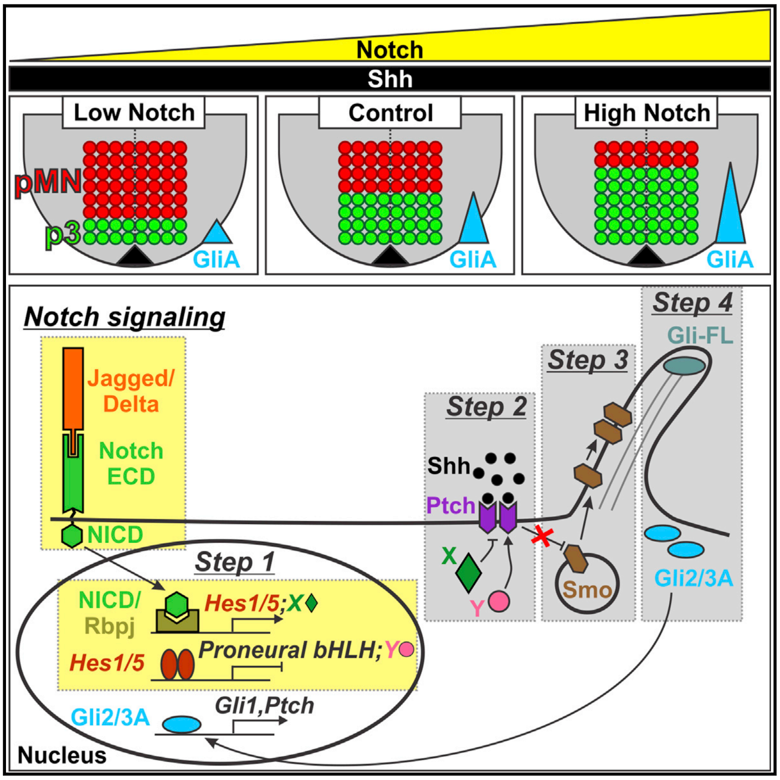

Highlights

- Changes in Notch signaling alter the dorsoventral identity of neural progenitors
- Activation and inactivation of Notch signaling alter cellular responses to Shh
- Notch activity is required for efficient trafficking of Smo to primary cilia
- Notch activity regulates the subcellular distribution of the Shh receptor Ptch1

Authors

Jennifer H. Kong, Linlin Yang, ...,
James Briscoe, Bennett G. Novitch

Correspondence

bnovitch@ucla.edu

In Brief

Cell fate assignment in the ventral spinal cord depends on the ability of neural progenitors to interpret the morphogen Shh. Kong and Yang et al. show that Notch signaling tunes neural progenitor responses to Shh by regulating trafficking of the Shh receptor Patched1 and downstream effector Smoothened to primary cilia.

Kong et al., 2015, Developmental Cell *33*, 373–387
May 26, 2015 ©2015 The Authors
http://dx.doi.org/10.1016/j.devcel.2015.03.005

Developmental Cell
Article

Notch Activity Modulates the Responsiveness of Neural Progenitors to Sonic Hedgehog Signaling

Jennifer H. Kong,[1,2,6] Linlin Yang,[1,2,6] Eric Dessaud,[3] Katherine Chuang,[1,2] Destaye M. Moore,[1,2] Rajat Rohatgi,[4,5] James Briscoe,[3] and Bennett G. Novitch[1,2,*]
[1]Department of Neurobiology
[2]Eli and Edythe Broad Center of Regenerative Medicine and Stem Cell Research
David Geffen School of Medicine at UCLA, Los Angeles, CA 90095, USA
[3]The Francis Crick Institute, Mill Hill Laboratory, London NW7 1AA, UK
[4]Department of Medicine
[5]Department of Biochemistry
Stanford University School of Medicine, Stanford, CA 94305, USA
[6]Co-first author
*Correspondence: bnovitch@ucla.edu
http://dx.doi.org/10.1016/j.devcel.2015.03.005
This is an open access article under the CC BY license (http://creativecommons.org/licenses/by/4.0/).

SUMMARY

Throughout the developing nervous system, neural stem and progenitor cells give rise to diverse classes of neurons and glia in a spatially and temporally coordinated manner. In the ventral spinal cord, much of this diversity emerges through the morphogen actions of Sonic hedgehog (Shh). Interpretation of the Shh gradient depends on both the amount of ligand and duration of exposure, but the mechanisms permitting prolonged responses to Shh are not well understood. We demonstrate that Notch signaling plays an essential role in this process, enabling neural progenitors to attain sufficiently high levels of Shh pathway activity needed to direct the ventral-most cell fates. Notch activity regulates subcellular localization of the Shh receptor Patched1, gating the translocation of the key effector Smoothened to primary cilia and its downstream signaling activities. These data reveal an unexpected role for Notch shaping the interpretation of the Shh morphogen gradient and influencing cell fate determination.

INTRODUCTION

Neuronal and glial diversity in the CNS emerges in large part through the concomitant and combinatorial actions of morphogen signals such as Sonic hedgehog (Shh), Bone Morphogenetic Proteins (BMPs), Wnts, and retinoids that organize neural progenitor cells (NPCs) into discrete domains along the dorsoventral and rostrocaudal axes (Briscoe and Novitch, 2008; Le Dréau and Martí, 2013; Butler and Bronner, 2015). Each of these domains is defined by its expression of unique combinations of transcription factors and ability to generate specific classes of neurons and glia (Briscoe and Novitch, 2008; Rowitch and Kriegstein, 2010; Le Dréau and Martí, 2013; Butler and Bronner, 2015). The prevailing model for morphogen signaling posits that differential cellular responses arise due to the signal concentrations that cells encounter (Rogers and Schier, 2011), yet the duration of exposure to a fixed amount of signal can also elicit graded domain responses and influence fate decisions (Kutejova et al., 2009). These results suggest that an important aspect of morphogen interpretation is the ability of cells to maintain their responsiveness to these cues as development proceeds. However, the mechanisms that permit this competence over time are not well understood.

One of the best studied examples of morphogen signaling is the patterning response of NPCs in the ventral spinal cord to Shh. Shh acts on NPCs in a dose-dependent manner, binding to its primary receptors Patched1 and 2 (Ptch1/2) to initiate a cascade of intracellular signaling events centered on the translocation of the G-protein-coupled receptor Smoothened (Smo) to primary cilia (Eggenschwiler and Anderson, 2007; Dessaud et al., 2008; Ribes and Briscoe, 2009). The presence of Smo in cilia modulates the proteolysis and activity of the Gli family of Zn-finger transcription factors, which in turn regulate the expression of many NPC fate determinants that subdivide the ventral spinal cord into three distinct ventral NPC domains: p3, pMN, and p2 (Briscoe and Novitch, 2008; Dessaud et al., 2008; Ribes and Briscoe, 2009). These domains are distinguished by their shared expression of the transcription factor Nkx6.1 and differential expression of Nkx2.2, Olig2, and Irx3, respectively (Mizuguchi et al., 2001; Novitch et al., 2001; Briscoe and Novitch, 2008; Dessaud et al., 2008). The pMN gives rise to motor neurons (MNs), while the p3 and p2 domains produce distinct classes of spinal interneurons that modulate MN activities. Later in development, Olig2^{+} NPCs form a domain of oligodendrocyte precursors (pOLs) that disperse and migrate throughout the spinal cord before differentiating into myelinating oligodendrocytes (Rowitch and Kriegstein, 2010). The p3 and p2 domains similarly transform into astroglial progenitor groups (pVA3 and pVA2), producing astrocytes that colonize distinct regions of the ventral spinal cord (Muroyama et al., 2005; Hochstim et al., 2008).

While these fates can be specified through the administration of different concentrations of Shh ligand in vitro (Dessaud et al., 2008; Ribes and Briscoe, 2009), NPCs also acquire their ventral identities through time-dependent mechanisms. NPCs treated

CellPress

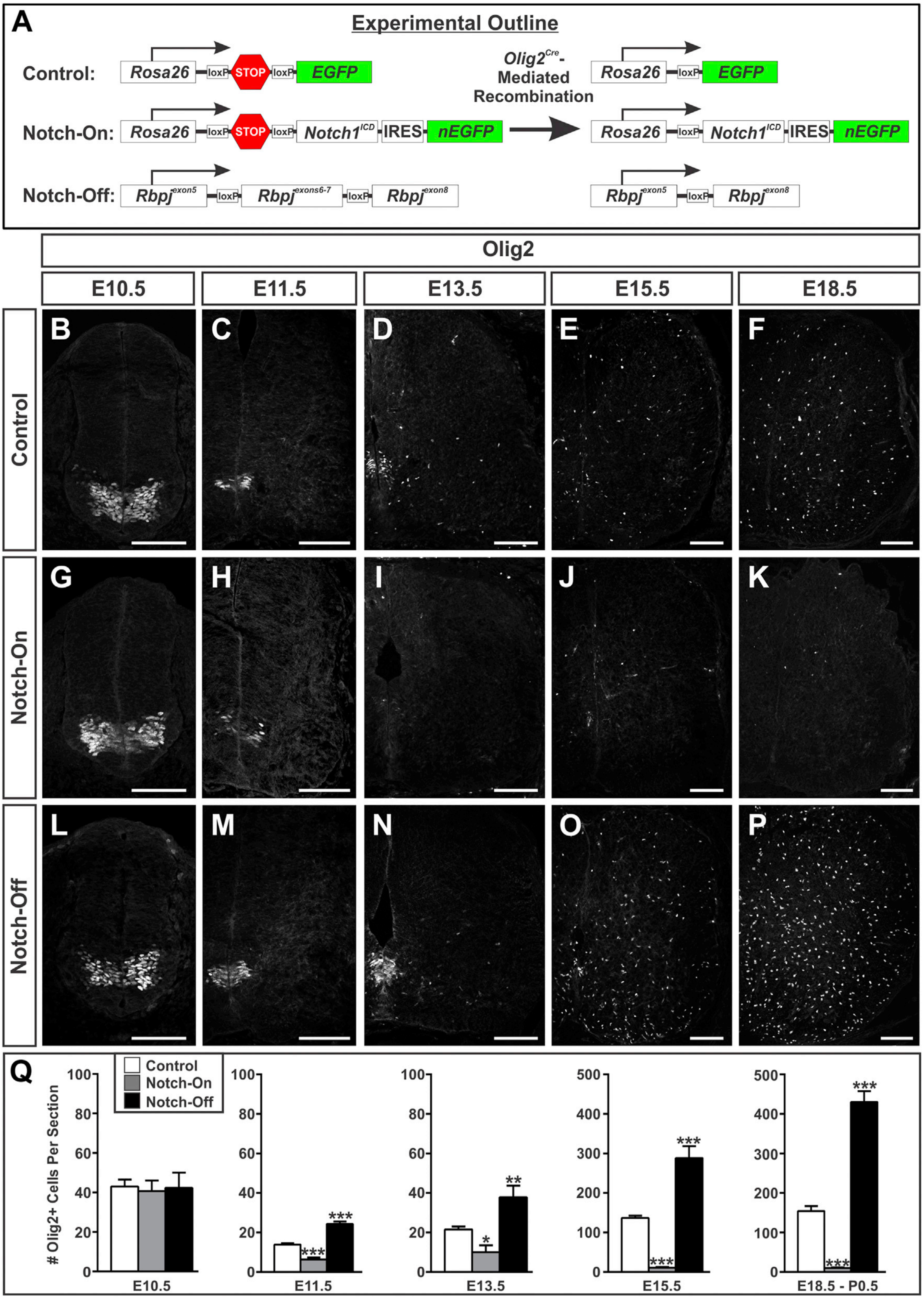

Figure 1. Manipulation of Notch Signaling Alters Olig2 Expression

(A) Schematic of *Olig2*^{Cre}-mediated manipulations used to activate or inactivate Notch signaling. Notch-On indicates NICD misexpression and Notch-Off indicates *Rbpj* deletion. Control conditions include crosses to mice carrying a *R26R*^{GFP} reporter.

(B–F) At E10.5–E11.5, Olig2 is initially expressed by MN progenitors and later oligodendrocyte progenitors.

(legend continued on next page)

with moderate doses of Shh initially express the pMN determinant Olig2; however, if Shh/Gli signaling is sustained, they subsequently express Nkx2.2 and adopt the more ventral p3 fate (Dessaud et al., 2007, 2010; Balaskas et al., 2012). Recent studies in the zebrafish spinal cord have further demonstrated that progenitor maintenance mediated by the Notch signaling pathway plays an important role enabling later born Shh-induced cell types to emerge (Huang et al., 2012). Together, these findings indicate that cells must remain in an undifferentiated state to properly interpret the Shh morphogen gradient, but do not resolve the mechanism by which the maintenance of NPC characteristics influences Shh responsiveness and whether retaining cells in a progenitor state influences spatial patterning.

The Notch signaling pathway serves as a major regulator of NPC maintenance and both neuronal and glial development (Gaiano and Fishell, 2002; Pierfelice et al., 2011). Notch receptors are broadly expressed by NPCs and are activated by the Delta-like and Jagged families of transmembrane ligands presented by neighboring cells (Kageyama et al., 2009; Pierfelice et al., 2011). Activated Notch receptors are cleaved by the Presenilin γ-secretase complex, liberating Notch intracellular domain (NICD) fragments. NICD subsequently forms transcriptional activating complexes with the DNA binding protein Rbpj and members of the mastermind-like (MAML) coactivator family (Kageyama et al., 2009; Pierfelice et al., 2011). Rbpj-NICD-MAML complexes regulate a number of targets most notably Hes genes, bHLH transcription factors that repress proneural genes, inhibit neuronal differentiation, and promote NPC maintenance (Kageyama et al., 2007, 2009; Pierfelice et al., 2011). Through these actions, Notch signaling suppresses neuronal differentiation and endows cells with gliogenic potential. NICD misexpression can further promote specific glial cell fates, such as radial glia in the forebrain, Müller glia in the retina, and astrocytes in neural stem cell cultures (Furukawa et al., 2000; Gaiano et al., 2000; Scheer et al., 2001; Ge et al., 2002) while inhibiting oligodendrocyte differentiation (Wang et al., 1998). These data implicate a role for Notch in glial fate selection, although the mechanisms underlying these effects remain unclear.

Here, we test the contributions of Notch signaling on both the establishment of NPC identities and glial fate determination. We show that activation and inactivation of the Notch pathway modify the responses of NPCs to Shh, altering both their dorsoventral register and ability to generate distinct classes of neurons and glial cells. Notch activity strikingly acts at the most proximal steps in the Shh transduction pathway, affecting the trafficking of Smo and Ptch1 to primary cilia. Together, these findings reveal a role for Notch signaling shaping the interpretation of the Shh morphogen gradient and assignment of cell fates.

RESULTS

Manipulation of Notch Signaling Alters the Dorsoventral Register of NPCs

We first used *Olig2^{Cre}* mice (Dessaud et al., 2007) to selectively activate or inactive Notch signaling in the p3 and pMN domains between embryonic days (E) 9.5 and 10.5 (Figures S1A–S1W). This strategy was accomplished by crossing *Olig2^{Cre}* to mice harboring (1) a Cre-inducible *R26R^{GFP}* transgenic reporter (Mao et al., 2001) (control condition), (2) a *R26R$^{NICD-GFP}$* transgene and reporter (Murtaugh et al., 2003) (“Notch-On” condition), or (3) a Cre-inactivatable *Rbpj* allele (Han et al., 2002), along with the *R26R^{GFP}* transgenic reporter (“Notch-Off” condition) (Figures 1A). The impact of these Notch pathway manipulations was evident by E11.5, as Notch-On mice displayed elevated expression of the Notch effectors Hes1 and *Hes5,* which are normally very low in the pMN and reduced expression of proneural transcription factors, including Neurog2, Ascl1, and *Neurog3* (Figures S2A–S2N). Conversely, Notch-Off mice displayed reductions in Hes1 and *Hes5* expression and increased levels of Neurog2, Ascl1, and *Neurog3* (Figures S2O–S2U). While the ventral ventricular zone (VZ) narrowed in Notch-Off mice, a contiguous band of Sox2$^+$ NPCs was maintained throughout development, and both the neuroepithelial architecture and apicobasal polarity of progenitors were preserved (Figures S2V–S2AI). This phenotype contrasts with mutations in other members of the Notch pathway such as *Hes1* and *Hes5* whose combined loss disrupts the neuroepithelium (Hatakeyama et al., 2004). The persistence of NPCs and neuroepithelial organization in *Olig2^{Cre}*;Notch-Off mutants may be explained by the lasting presence of Hes1 in ventral progenitors despite the loss of *Rbpj* (Figures S2Q and S2R), most likely due to Notch-independent activation of Hes1 by Shh, as has been described in other tissues (Ingram et al., 2008; Wall et al., 2009).

We next examined the impact of these Notch manipulations on dorsoventral patterning. Remarkably, activating Notch signaling led to a notable reduction in Olig2$^+$ pMN cells by ~E11.5 and a nearly complete loss of Olig2$^+$ NPCs throughout the rest of embryonic development (Figures 1B–1K and 1Q). Notch-Off mice exhibited the reciprocal phenotype, with an ~1.5 to ~2.5-fold increase in the number of Olig2$^+$ progenitors from E11.5 to postnatal day (P) 0.5 (Figures 1L–1Q). While Olig2$^+$ cells were reduced in Notch-On mice, the overall number of ventral NPCs expressing Nkx6.1 increased by ~50% (Figure 2M). The loss of Olig2 from Nkx6.1$^+$ NPCs coincided with the increased expression of the p3 determinant Nkx2.2 (Figures 2A–2H and 2N). Given that Nkx2.2 can repress Olig2 (Mizuguchi et al., 2001; Novitch et al., 2001; Sun et al., 2003), the loss of pMN cells in Notch-On mice is likely due to their transformation toward the more ventral p3 fate. This conclusion was supported by the reduced percentage of Nkx6.1$^+$ progenitors expressing Nkx2.2 and corresponding increase in Olig2$^+$ cells seen in Notch-Off spinal cords (Figures 2I–2L and 2N). Collectively, these data demonstrate that Notch signaling plays a critical role enhancing the ventral character of NPCs and influencing their partitioning between pMN and p3 identities.

Notch-Mediated Changes in Ventral NPCs Alter Neuronal and Glial Fates

We next used *R26R^{GFP}* lineage tracing to assess the fate of the Notch-manipulated cells. Consistent with the loss of Olig2,

(G–P) In Notch-On mice, Olig2$^+$ cells decline from E11.5 onward. In Notch-Off mice, Olig2$^+$ cells increase. Scale bars represent 100 μm.

(Q) Quantification of Olig2$^+$ cells per spinal cord half at the indicated time points. Plots show the mean ± SEM from multiple sections collected from 4–25 embryos from each group. *$p < 0.05$, **$p < 0.01$, ***$p < 0.001$.

See also Figures S1 and S2.

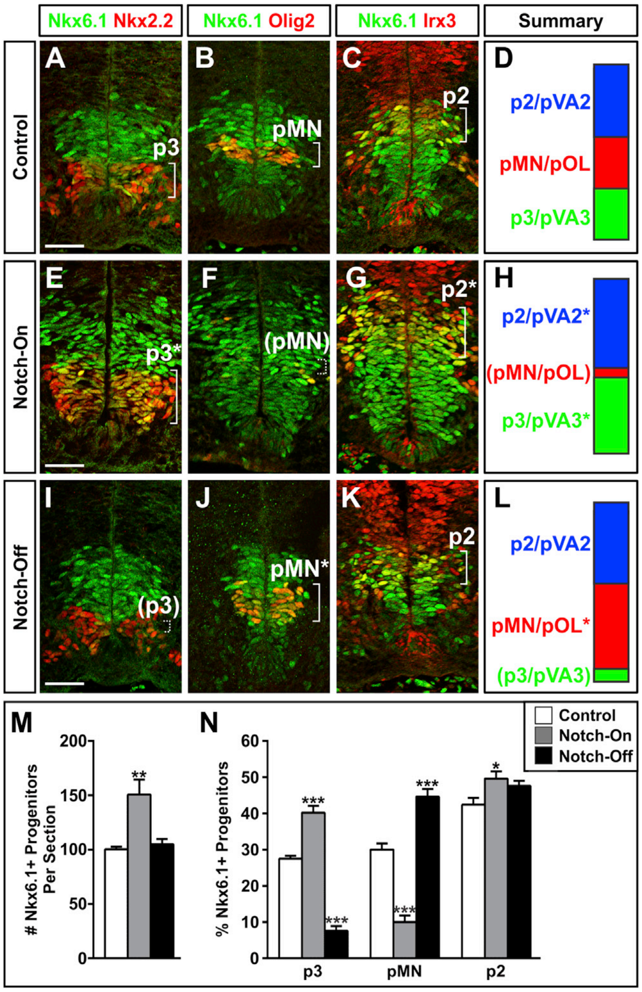

Figure 2. Changes in Notch Signaling Alter the Dorsoventral Identities of Ventral Spinal Cord Progenitors

(A–D) In E11.5 control spinal cords, ventral progenitors are distinguishable by coexpression of Nkx6.1 and Nkx2.2 (p3), Nkx6.1 and Olig2 (pMN), and Nkx6.1 and Irx3 (p2).

(E–H) More Nkx6.1^{+} progenitors are present in Notch-On mutants. Within this population, the percentage expressing Nkx2.2 increased while the percentage expressing Olig2 decreased.

(I–L) Notch-Off mutants contain a reduced percentage of Nkx6.1^{+} progenitors expressing Nkx2.2 and reciprocal increase in Olig2. Scale bars represent 50 μm.

(M and N) Quantification of the total number of Nkx6.1^{+} progenitors present and their subdivision into p3, pMN, and p2. Plots show the mean ± SEM from multiple sections collected from seven to nine embryos for each group. *$p < 0.05$, **$p < 0.01$, ***$p < 0.001$.

See also Figures S2 and S4.

Notch-On spinal cords exhibited an ~35% reduction in MN formation (Figures S3A–S3F and S3J–S3L). Most of this deficit resulted from the selective loss of Foxp1^{+} lateral motor column (LMC) MNs at limb levels and preganglionic column (PGC) MNs at thoracic levels, with little change to Foxp1^{-} medial and hypaxial motor column (MMC and HMC) MNs (Figure S3K) (Rousso et al., 2008). LMC and PGC MNs are among the last MN subtypes to be formed (Tsuchida et al., 1994), suggesting that Notch activity must be silenced for the generation of these later-born cell types. Nevertheless, Notch-Off spinal cords did not exhibit any obvious defects in either MN formation or segregation into different columnar subgroups (Figures S3G–S3L).

Olig2^{Cre}-mediated Notch manipulations produced more striking changes in glial fate selection. In E18.5 control embryos, *Olig2^{Cre}* derivatives include both Sox10^{+} Pdgfrα^{+} oligodendrocyte progenitors scattered throughout the spinal cord and BLBP^{+} Nf1A^{+} Nkx6.1^{+} Fgfr3^{+} Slit1^{+} VA3 astrocyte precursors and differentiated astrocytes located in the ventral-most white matter (Figures 3 and S3M–S3U) (Hochstim et al., 2008). Notch-On spinal cords exhibited a nearly complete loss of pOLs and corresponding increase in VA3-like astrocyte precursors (Figures 3A–3H, 3M–3O, and S3M–S3R) (Hochstim et al., 2008). Conversely, Notch-Off spinal cords produced more pOLs and fewer astrocyte precursors and differentiated VA3 astrocytes (Figures 3I–3O and S3S–S3U). Together, these data show that early changes in NPC fates following Notch pathway manipulation lead to corresponding alterations in neuronal and, more strikingly, glial identities.

Notch Signaling Is Only Able to Shift NPC Identities within the Ventral Spinal Cord

Previous studies observed that glial fates could be altered by deleting *Rbpj* function from all spinal NPCs (Taylor et al., 2007), raising the question of whether our results stemmed from direct effects of Notch activity on glial fate selection or were a secondary consequence of altered dorsoventral patterning. To distinguish between these possibilities, we examined the consequences of manipulating Notch activity in the p0 domain of the intermediate spinal cord using a *Dbx1^{Cre}* driver (Bielle et al., 2005; Dessaud et al., 2010). *Dbx1^{Cre}*-mediated Notch activation expanded the numbers of Dbx1^{+} and Dbx2^{+} progenitors (Figures S4A–S4D and S4G), while Notch inactivation disrupted neuroepithelial organization and depleted these cells (Figures S4E–S4S). Despite these effects, we observed no changes in the dorsoventral register of NPCs or shifts in glial identities as seen with *Olig2^{Cre}*-based manipulations (Figures S4T–S4AI). Thus, while manipulation of the Notch pathway can change the balance between NPC maintenance and differentiation within the intermediate spinal cord, it appears insufficient to evoke changes in dorsoventral patterning and associated shifts in neuronal and glial fates.

Notch Signaling Alters Ventral Progenitor Identities by Modulating Responses to Shh

The selective effects of Notch activity on cell fate assignment in the ventral versus intermediate spinal cord suggests that Notch modulates the responsiveness of NPCs to Shh ligand produced at the ventral midline. To test this possibility, we used a chick intermediate [i] neural plate explant system to examine the fates of NPCs exposed to moderate (1 nM) or high (4 nM) amounts of Shh and varying amounts of the γ-secretase inhibitor DAPT (N-[N-(3,5-Difluorophenacetyl)-L-alanyl]-S-phenylglycine t-butyl ester) to reduce Notch receptor cleavage and downstream signaling (Dovey et al., 2001; Geling et al., 2002; Dessaud et al., 2007). High amounts of Shh produced numerous Nkx2.2^{+} p3 cells and a small number of Olig2^{+} pMN cells (Figure 4D), as previously described (Dessaud et al., 2007). However, when Notch

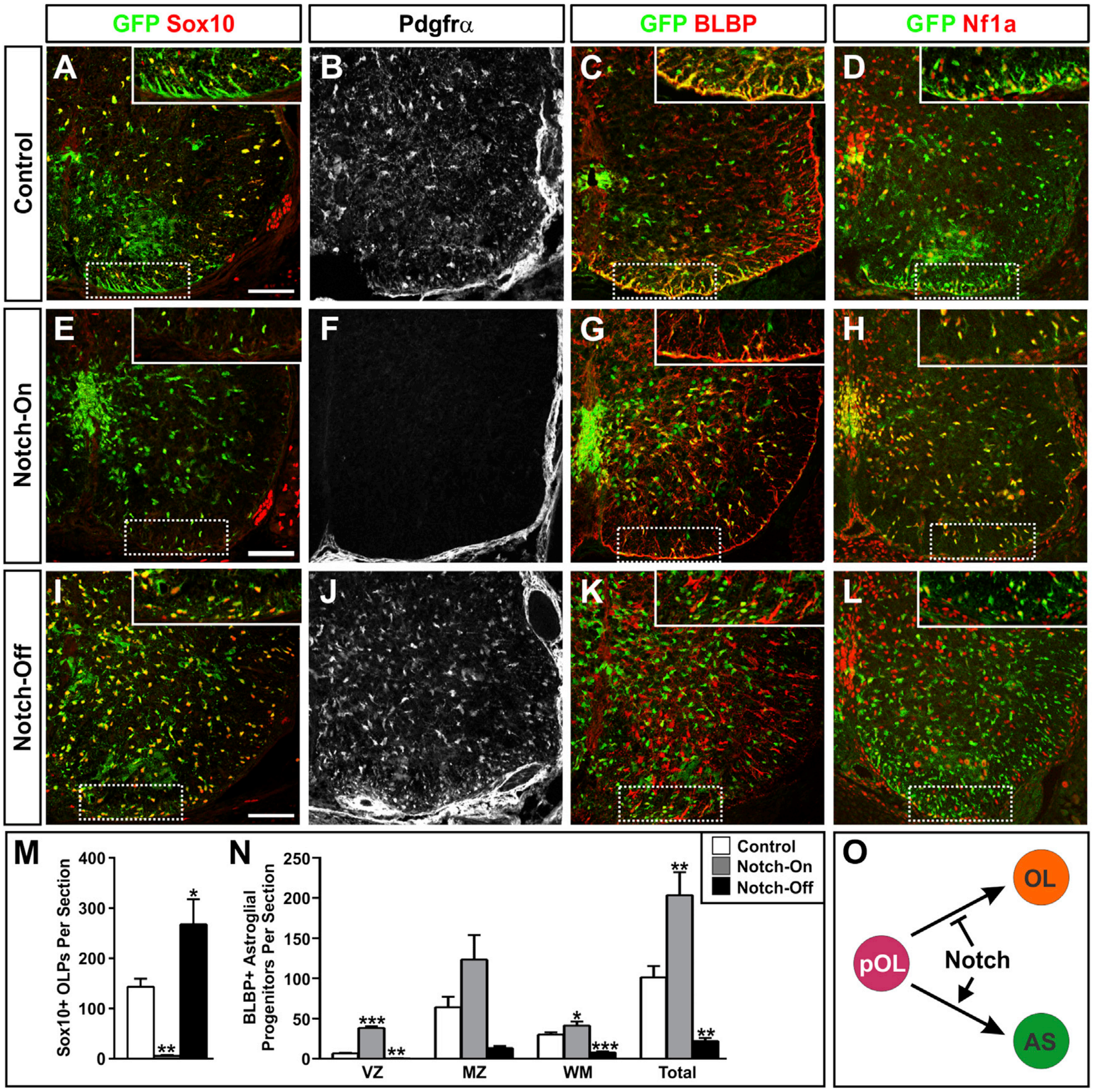

Figure 3. Manipulation of Notch Signaling Alters Glial Fates

(A–D) In E18.5 control spinal cords, *Olig2*^{Cre};*R26R*^{GFP}-labeled descendants include Sox10$^+$/PDGFRα$^+$ oligodendrocyte precursors (OLPs), BLBP$^+$/NF1a$^+$ pVA3 astrocyte progenitors.

(E–H) Notch activation suppresses OLP formation and expands pVA3 progenitors.

(I–L) Notch inactivation expands OLP production at the expense of pVA3 progenitors. Scale bars represent 100 μm.

(M and N) Quantification of total OLP (GFP+/Sox10$^+$) and pVA3 astrocyte progenitors (GFP+/BLBP$^+$) per spinal cord half. pVA3 counts are divided based on localization within the VZ, marginal zone (MZ), or white matter (WM). Plots show the mean ± SEM from multiple sections collected from three to seven embryos for each group. *$p < 0.05$, **$p < 0.01$, ***$p < 0.001$.

(O) Summary of the role of Notch signaling in directing glial fate choices.

See also Figures S3 and S4.

activity was reduced using DAPT, the number of Nkx2.2$^+$ progenitors was reduced while Olig2$^+$ cells increased (Figures 4E and 4F), recapitulating the phenotype seen in Notch-Off mice (Figures 2I, 2J, and 2N). Interestingly, the effects of DAPT up to 25 μM appeared selective, as it blunted the Nkx2.2-inducing activity of high doses of Shh but did not block the Olig2-inducing activity of lower doses of Shh (Figures 4A–4C). These results suggest Notch is required for NPCs to experience high but not low levels of Shh signaling.

To verify that these NPC identity shifts were due to effects of Notch on Shh pathway activity, [i] explants were isolated from chick embryos electroporated with a Gli binding site-luciferase (GBS-luciferase) reporter to measure Gli function after Shh administration (Stamataki et al., 2005; Dessaud et al., 2007). DAPT addition led to a >50% decrease in GBS-luciferase activity over that seen with Shh alone (Figure 4G). Similar results were obtained with measurement of GBS-luciferase activity in ventral neural plate plus floor plate [vf] explants, in which Gli activity is

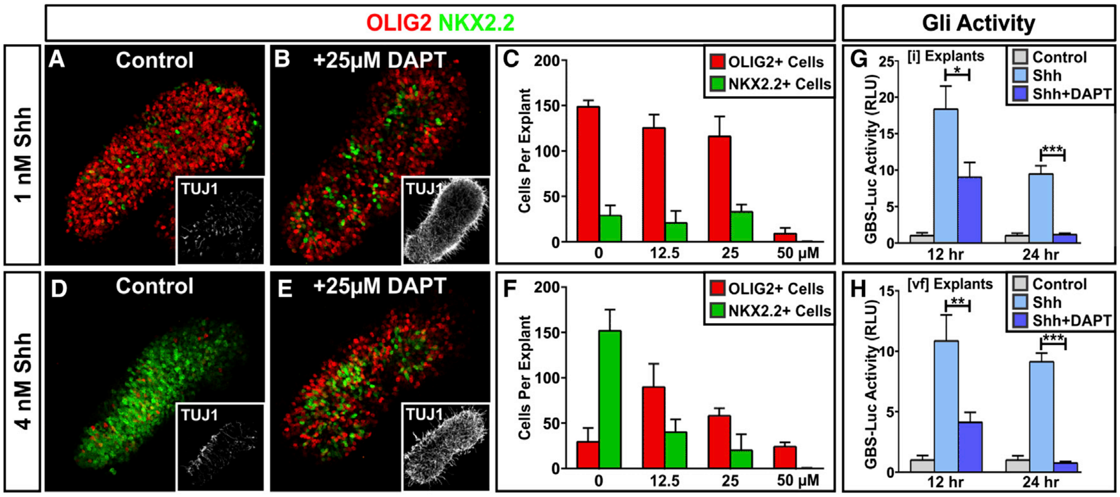

Figure 4. Inhibition of Notch Signaling Reduces Gli Activity and Assignment of the p3 Fate

(A, B, D, and E) Representative images of HH stage 10 chick intermediate neural plate [i] explants cultured for 24 hr in 1- or 4-nM Shh ± 25-μM DAPT. Explants were stained with Nkx2.2 and Olig2 antibodies to identify p3 and pMN cells. Insets show DAPT addition increases Tuj1^{+} neurons, as expected for a Notch inhibitor.

(C and F) Quantification of p3 and pMN cells present in [i] explants cultured in either 1- or 4-nM Shh and varying amounts of DAPT (0–50 μM). n ≥ 5 explants per condition and plots display cells/explant ± SEM.

(G) Gli activity measurements of [i] explants isolated from chick embryos electroporated with a GBS-Luciferase reporter construct and cultured with or without 4-nM Shh ± 25-μM DAPT. n ≥ 5 explants per condition were collected; plots display relative GBS-luciferase activity (relative light units) ± SEM.

(H) Gli activity measurements in [vf] explants isolated from embryos electroporated with the GBS-luciferase reporter and cultured in the presence or absence of 25-μM DAPT. n ≥ 5 explants per condition; relative GBS-luciferase activity ± SEM. *$p < 0.05$, **$p < 0.01$, ***$p < 0.001$.

driven by the endogenous Shh produced by floor plate cells (Figure 4H). Collectively, these data demonstrate that Notch signaling is required for NPCs to attain the highest levels of Gli activity and assume the ventral-most fates.

Notch Signaling Facilitates the Accumulation of Smo within Primary Cilia

We next sought to determine a mechanism that could explain the modulatory effects of Notch signaling on Shh responsiveness. Given that the requirement of Notch for Shh responses appears to be conserved in NPCs across species, we tested whether it was also conserved across cell types. NIH 3T3 mouse fibroblasts are a cell line shown to be Notch responsive (Small et al., 2003) and in which the cellular and molecular details of Shh signaling are well established (Taipale et al., 2000; Rohatgi et al., 2007; Tukachinsky et al., 2010). We first validated the system by exposing Shh-Light2 cells, a NIH 3T3 derivative stably transfected with a GBS-luciferase reporter, to increasing concentrations of Shh and observed dose-dependent increases in luciferase activity (Figure 5A). The addition of DAPT to these cultures strikingly reduced Shh-induced GBS-Luciferase activity (Figure 5B), recapitulating the effects seen with neural plate explants (Figures 4D–4H). Quantitative PCR (qPCR) analysis showed that DAPT similarly impacted endogenous Shh response genes such as *Gli1* and *Ptch1* (Figure 5C).

We then used the NIH 3T3 fibroblast system to pinpoint where Notch activity acts in the Shh transduction cascade. One of the first steps is the translocation of Smo to primary cilia, which initiates the conversion of Gli proteins into transcriptional activators (Corbit et al., 2005; Rohatgi et al., 2007). DAPT dramatically reduced Shh-induced Smo accumulation within primary cilia, acting in a dose-dependent manner (Figures 5D–5F, 5I–5K, and S5A). This change occurred without any obvious impact on *Smo* mRNA, alterations in cell polarity, or presence of primary cilia, although DAPT addition alone reduced average cilia length by 12.6% ± 1.3%, $p < 0.001$ (Figures 5C and S5B–S5I). To confirm that reductions in ciliary Smo were due to changes in Notch pathway activity, we repeated these experiments using two additional small molecule inhibitors: SAHM1, a peptide that prevents assembly of the NICD-Rbpj-MAML1 transcriptional activator complex (Moellering et al., 2009), and JLK6 (7-Amino-4-chloro-3-methoxyisocoumarin, also referred to as γ-secretase inhibitor XI), a molecule that blocks activation of some γ-secretase targets such as beta-amyloid precursor proteins while sparing others, including the Notch receptors (Petit et al., 2001). Verifying these activities, we found that both DAPT and SAHM1 reduced *Hes1* gene expression in NIH 3T3 cells by ~65%–75%, whereas JLK6 had no discernible effect (Figure 5I). Importantly, SAHM1 reduced Shh-induced ciliary accumulation of Smo in a manner similar to DAPT (Figures 5G and 5J). JLK6 in contrast had no effect on Smo localization (Figures 5H and 5K).

We further tested whether the impact of Notch activity on Shh-induced Smo localization was limited to NIH 3T3 cells or more broadly applicable to other cell types including human NPCs, primary mouse embryonic fibroblasts (MEFs), and C2C12 mouse myoblasts. In all cases, DAPT reduced Shh-induced Smo accumulation within primary cilia (Figures S6A–S6M), suggesting that the cross-talk between the Notch and Shh pathways is conserved across germ layers and species.

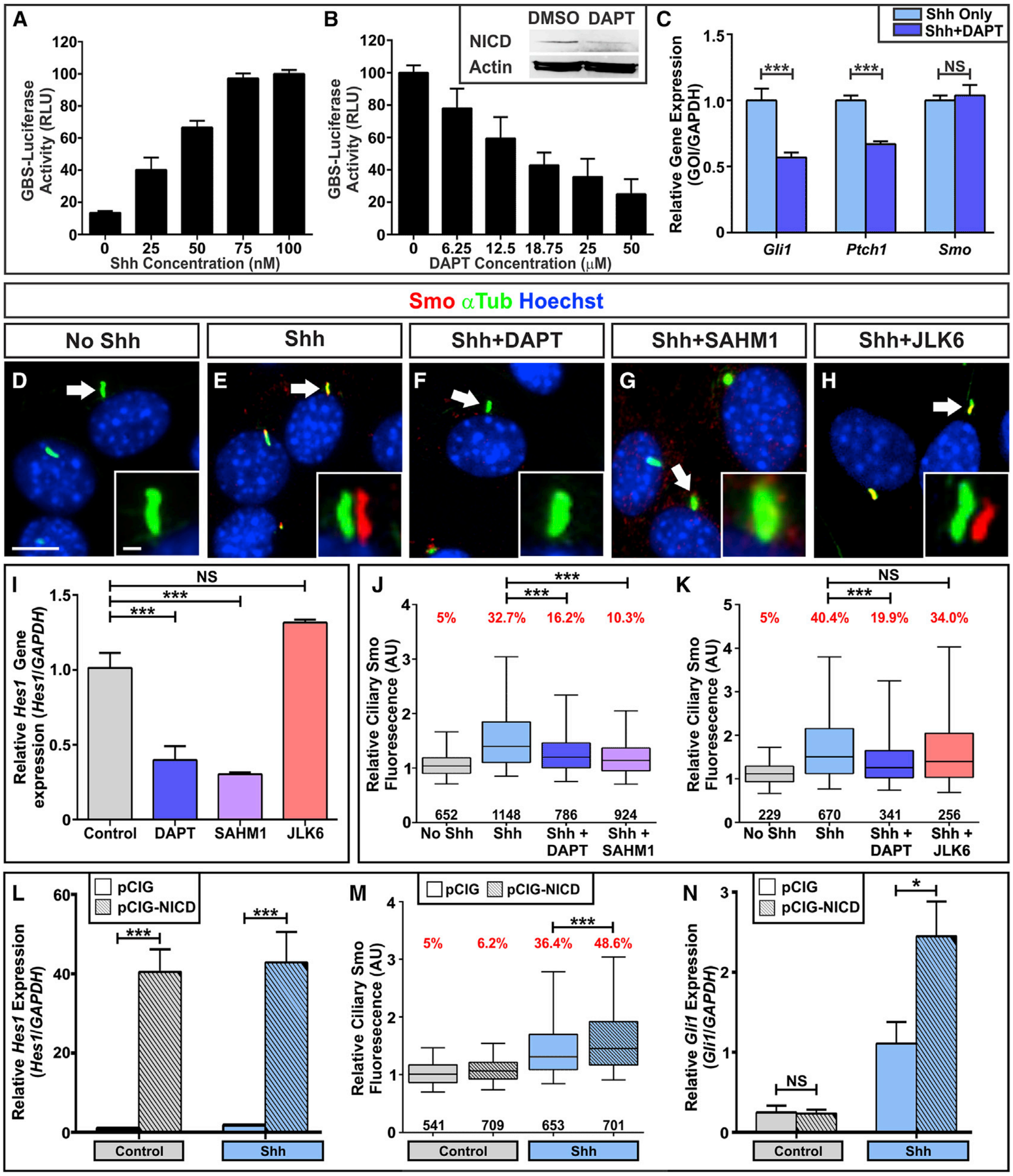

Figure 5. Notch Signaling Regulates the Ciliary Location of Smo and Shh Pathway Activity in Fibroblasts

(A and B) GBS-luciferase reporter activity in NIH 3T3 Shh-LIGHT2 cells cultured in either Shh (0–100 nM) or a range of DAPT (0–50 μM) in the presence of a single concentration of Shh (50 nM). Points represent mean GBS-luciferase activity (relative light units) ± SEM from four to six independent samples. Inset shows immunoblotting for cleaved NICD and actin.

(C) qPCR analysis of *Gli1*, *Ptch1*, and *Smo* expression in NIH 3T3 cells cultured in Shh (50 nM) ± DAPT (18.75 μM). Plot shows mean *Gapdh*-normalized gene expression levels ± SEM from six samples. Not significant (NS), $p > 0.05$, ***$p < 0.001$.

(D–H) Changes in the localization of Smo to primary cilia of NIH 3T3 cells treated with Shh and Notch inhibitors (DAPT, 18.75 μM and SAHM1, 20 μM) or a γ-secretase inhibitor that spares Notch function (JLK6, 20 μM). Cells were immunostained for αTubulin (αTub) (green), Smo (red), and Hoechst (blue, nuclei). Arrows denote cilia in the insets where Smo and αTub channels are offset to show colocalization. Low- and high-magnification scale bars represent 10 and 1 μm.

(I) qPCR analysis of *Hes1* in NIH 3T3 cells exposed to DAPT (18.75 μM), SAHM1 (20 μM), or JLK6 (20 μM). Plots show mean *Gapdh*-normalized mRNA expression levels relative to unstimulated controls ± SEM from three to five samples. *$p < 0.05$, **$p < 0.01$, ***$p < 0.001$.

(J and K) Box and whisker plots of Smo fluorescence in the cilia of NIH 3T3 cells treated as indicated. The number of cilia analyzed in each group is indicated in black. The percentage of cilia with Smo is indicated in red. NS, $p > 0.05$, ***$p < 0.001$.

(L and N) qPCR analysis of *Hes1* and *Gli1* in NIH 3T3 cells transiently transfected with pCIG or pCIG-NICD vectors and then cultured in the presence or absence of Shh (50 nM). Plots show mean *Gapdh*-normalized expression levels relative to pCIG controls ± SEM from five to six samples for each condition.

(M) Box and whisker plots of the ciliary Smo fluorescence in transfected cells. NS, $p > 0.05$, *$p < 0.05$, ***$p < 0.001$.

See also Figures S5 and S6.

Since Notch inhibition reduced both the presence of Smo within primary cilia and Shh pathway activity, we tested whether the converse was also true. NIH 3T3 cells were transiently transfected with a vector expressing *NICD* and an IRES-*nEGFP* reporter cassette to activate Notch signaling, and both Smo localization and the expression of Shh-target genes evaluated. *NICD*-transfected cells exhibited an ~40-fold increase in *Hes1* expression irrespective of Shh stimulation (Figure 5L). Primary cilia were also slightly longer (17.5% ± 3.9%, $p < 0.001$) in *NICD*-transfected cells compared with *nEGFP*-only transfection controls, consistent with the reduced cilia lengths seen with DAPT addition. Upon Shh treatment, *NICD*-transfected cells exhibited an increase in the level of Smo within primary cilia and ~2- to 3-fold higher levels of *Gli1* expression (Figures 5M and 5N). These effects were only seen after the addition of Shh. Together, these results illustrate that Notch activity is not only required for Shh responsiveness, but can also potentiate its signaling function.

Given that *Hes1* was notably changed in all of our Notch manipulations, we tested whether direct elevation of *Hes1* could similarly increase cellular responses to Shh ligand. Interestingly, *Hes1* misexpression was sufficient to increase Shh-evoked activation of *Gli1* ~1.8-fold (Figures S5J–S5K). Collectively, these results suggest that the potentiating effects of Notch on Shh signaling result from activation of Hes genes and likely other downstream effectors.

Given the ability of Notch signaling to promote localization of Smo to cilia in cultured cells, we examined whether this effect could also be seen in the developing spinal cord. In E10.5 control embryos, high amounts of Smo were present in the cilia of both floor plate and Nkx2.2$^+$ p3 cells and lower levels present in Olig2$^+$ pMN cells (Figures 6A–6B″). In Notch-Off spinal cords, most *Olig2^{Cre}*-derived NPCs exhibited lower levels of ciliary Smo, and this change preceded shifts in Olig2 and Nkx2.2 expression (Figures 6C–6D″ and 6K). By E11.5, the extent of Smo localization within cilia along the dorsoventral axis of Notch-Off mutants was reduced by ~60% compared with littermate controls (Figures 6E–6F′ and 6I–6L). Notch-On mutants by contrast showed a dorsal expansion in the extent of Smo localization within primary cilia (Figures 6G–6H′ and 6L).

Changes in the ciliary accumulation of Smo following Notch manipulations could stem from either direct effects of Notch on Smo trafficking or indirect effects related to Notch having altered NPC identities. To distinguish between these possibilities, we examined Smo staining in the spinal cords of *Nkx2.2*, *Olig2*, and *Pax6* mutant mice, where dorsoventral patterning is known to be severely disrupted (Dessaud et al., 2008). Remarkably, the dorsal limits of ciliary Smo in all mutants were similar to control littermates, despite clear changes in NPC fates (Figures S7A–S7R). In *Nkx2.2* mutants, this alteration permitted the

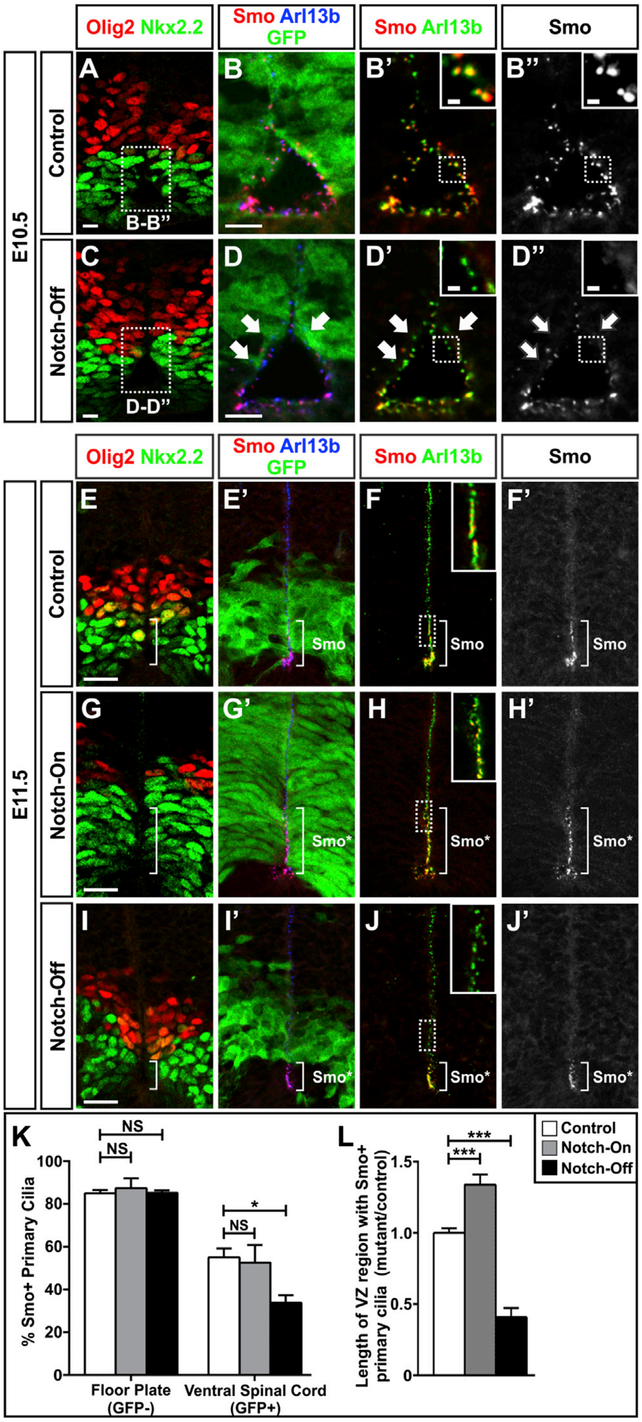

Figure 6. Notch Signaling Influences the Ciliary Accumulation of Smo in Ventral Spinal Cord NPCs

(A–D″) Analysis of Smo$^+$ primary cilia present on ventral progenitors in E10.5 embryos. Arrows in (D) indicate regions of Cre recombination. In Notch-Off embryos, Smo is absent from cilia in the recombined regions. Low (A and C) and high (B and D) magnification scale bars represent 10 and 2 μm.

(E–J′) Analysis of primary cilia in E11.5 embryos. Brackets illustrate the dorsoventral extent of Smo$^+$ primary cilia, a region where Nkx2.2$^+$ p3 cells are present. Scale bars represent 20 μm.

(K) Quantification of Smo$^+$ primary cilia at E10.5 counted from the GFP- floor plate and GFP$^+$ ventral progenitors. Plots show the mean percentage of Smo$^+$ primary cilia ± SEM from multiple sections collected from three to four embryos from each group. NS, $p > 0.05$ and *$p < 0.05$.

(L) Quantification of the dorsoventral limits of Smo$^+$ primary cilia at E11.5. Plots show mean lengths of the VZ lined with Smo$^+$ cilia ± SEM. All measured lengths were normalized to littermate controls. Analysis was conducted on multiple sections collected from three to nine embryos from each experimental group. ***$p < 0.001$.

See also Figure S7.

unusual presence of Olig2 in cells exhibiting high amounts of Smo in their cilia (Figures S7J and S7N), a phenotype that was never seen in control embryos or those in which Notch activity had been manipulated (Figures 6E–6L). Collectively, these data show that Notch activity influences Smo accumulation within primary cilia in multiple cell types in vitro and spinal cord NPCs in vivo and acts upstream of the transcription factor network controlling dorsoventral fates.

Notch Activity Sets the Levels of Ptch1 Present in Primary Cilia, Thereby Gating Smo Entry

We next considered the mechanism by which Notch might impact Smo localization. Our observations that Notch activation only promoted the accumulation of Smo within cilia following Shh addition suggested that it most likely acts upstream of Smo in the Shh transduction cascade. Consistent with this model, we found that DAPT was unable to block Smo accumulation when cells were treated with either Purmorphamine (Pur) or Smoothened Agonist (SAG), small molecules that directly stimulate Smo activity in a Shh ligand-independent manner (Chen et al., 2002; Sinha and Chen, 2006) (Figures 7A–7E). We thus focused our attention on the actions of Notch on the Shh receptor Ptch1. In the absence of ligand, Ptch1 localizes around the base and within primary cilia, where it inhibits Smo entry and Gli activation (Rohatgi et al., 2007). Shh binding to Ptch1 promotes its exit from primary cilia and concomitant Smo accumulation (Rohatgi et al., 2007). Since endogenous Ptch1 protein was difficult to detect in NIH 3T3 cells by antibody staining, we utilized Ptch1-YFP MEFs generated by infection of *Ptch1*$^{LacZ/LacZ}$ mutant cells with a retrovirus expressing a Ptch1-YFP fusion protein (Rohatgi et al., 2007). In the absence of Shh, ~75% of primary cilia contained Ptch1 (Figures 7F and 7J). When DAPT was added for 12 hr, the number of Ptch1^{+} primary cilia increased to ~90% (Figures 7G and 7J). DAPT was also able to impede the clearance of Ptch1 from primary cilia upon Shh stimulation (Figure 7H–7J). Remarkably, the effects of DAPT on Ptch1 localization occurred without any change in either *Ptch1* mRNA or protein levels in both Ptch1-YFP MEFs and NIH 3T3 cells (Figures S8G–S8I).

These results prompted us to examine whether the effects of DAPT on Smo trafficking to primary cilia occur immediately after its addition or rather require more time to enable Ptch1 to increase and thereby block Smo entry. Smo normally accumulates in primary cilia within 4 hr of Shh addition (Rohatgi et al., 2007) (Figures S8A and S8B). When Shh and DAPT were coadministered, there was no decrease in Smo presence within primary cilia at either the 4- or 6-hr time points; rather, Smo reduction only became evident after ~12 hr (Figures S8A and S8B). In contrast, when cells were pretreated with DAPT for 8 hr and then exposed to Shh plus DAPT for an additional 4 hr, significant reductions in Smo ciliary accumulation were observed (Figures S8C and S8D). These data indicate that the suppressive actions of DAPT on Smo localization follow the time course of Ptch1 accumulation within primary cilia. We further found that the actions of DAPT required new transcription, as changes in Smo localization were partially blocked by coadministration of DAPT and the RNA polymerase inhibitor α-amanitin (Figures S8E and S8F). These results suggest that Notch modulates Ptch1 and Smo levels in and around primary cilia through a transcriptional mechanism.

To test whether Ptch1 mediates the inhibitory effects of DAPT on Smo, we measured the impact of DAPT addition to *Ptch1*$^{LacZ/LacZ}$ mutant MEFs. Whereas DAPT potently inhibited Smo accumulation in the cilia of Shh-treated control MEFs, it was unable to do so in *Ptch1* null cells (Figures 7K–7O and S8J). Collectively, these data show that Notch signaling influences Smo accumulation by regulating the ciliary presence of Ptch1.

Finally, we tested whether altered localization or abundance of Ptch1 protein was observed after manipulations of the Notch pathway in the ventral spinal cord. In Notch-On mutants, Ptch1 protein staining in and around the primary cilia was notably reduced, fitting with the observed increase in Smo presence (Figures 6E–6H′, 7P, 7Q, and S7). In contrast, Notch-Off mutants showed elevated Ptch1 at the apical membrane and cilia in accordance with the reductions in Smo staining (Figures 6I–6J′, 7R, and 7S). Together, these in vitro and in vivo experiments demonstrate that Notch signaling plays an integral role modulating Ptch1 localization to gate Smo entry into primary cilia. Through these actions, Notch can regulate the downstream activation of the Shh transduction pathway and assignment of NPC fates.

DISCUSSION

It is well established that the dorsoventral identity of NPCs in the spinal cord and other regions of the CNS is influenced by the concentration of Shh ligand they are exposed to (Fuccillo et al., 2006; Dessaud et al., 2008; Ribes and Briscoe, 2009). However, Shh concentration is only part of the means though which graded signaling responses are achieved. Other important factors include (1) the duration of time over which cells are exposed to Shh, (2) the ability of cells to modulate their responsiveness to Shh through changes in the expression and/or subcellular distribution of key signal transduction components such as Ptch1 and Smo, (3) changes in the expression of proteins that modulate Shh-Ptch1 interactions or modify Shh itself, and (4) cross-regulatory interactions between Shh-regulated transcription factors that assign specific cell fates (Dessaud et al., 2008; Ribes and Briscoe, 2009; Briscoe and Thérond, 2013). Our studies show that Notch signaling plays a crucial role in these first two processes, serving to sustain NPCs in an undifferentiated, Shh-responsive state while also influencing the ciliary trafficking of Ptch1 and Smo and the downstream activation of Gli transcription factors (Figure 8). Together, these data provide important insights into the mechanisms through which NPCs interpret the Shh gradient and reveal a potentially general mechanism by which the Notch and Shh signaling pathways collaborate to direct cell fate decisions.

Notch-Mediated Changes in Shh Transduction Influence the Selection of NPC Fates

Our data show that manipulating the Notch pathway modulates the dorsoventral register of NPCs, with Notch activation and inactivation respectively increasing or decreasing the formation of the ventral-most cell types reflected by alterations in Nkx2.2 and

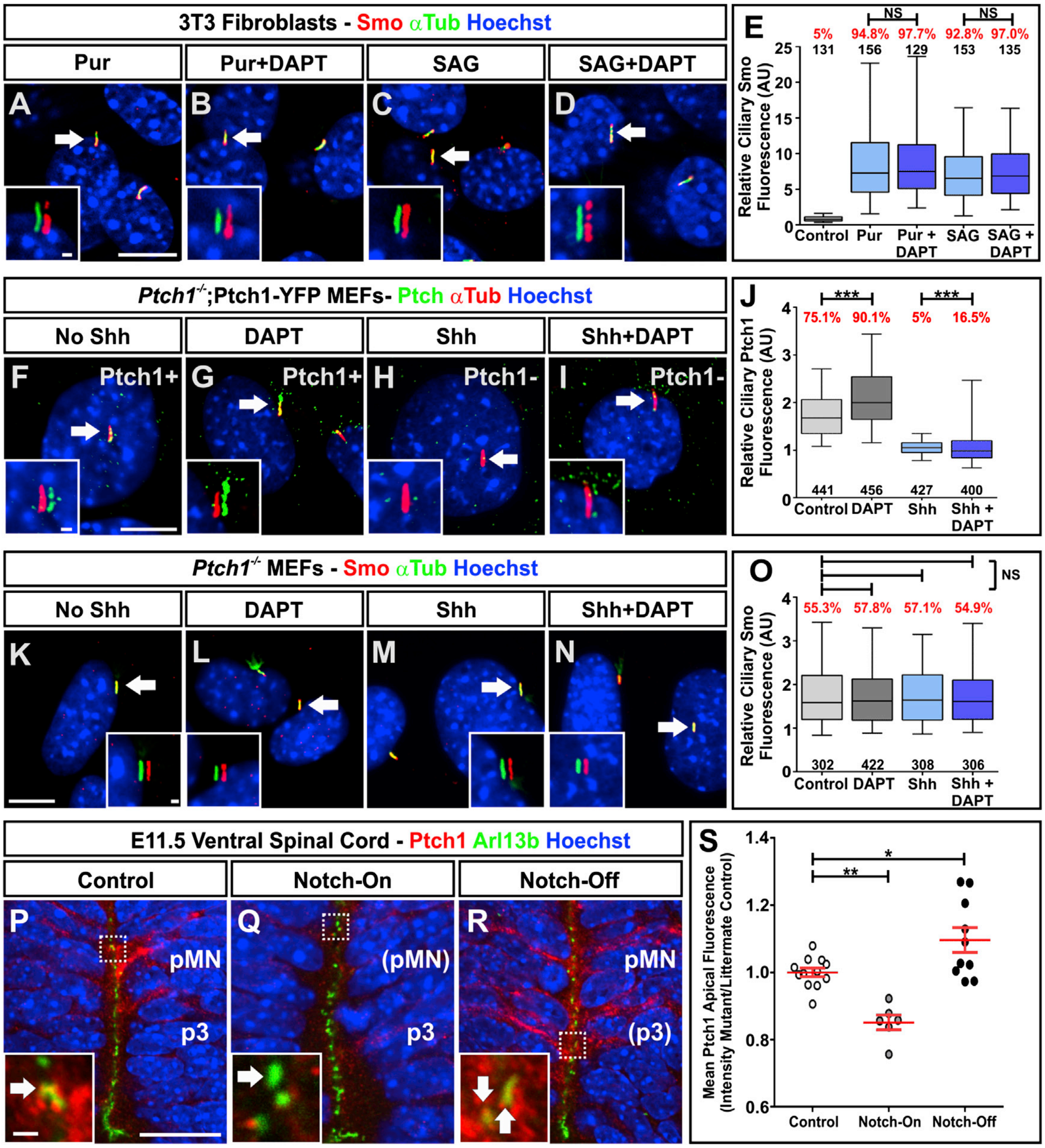

Figure 7. Notch Signaling Regulates Ptch1 Presence in and around Primary Cilia

(A–D) Analysis of Smo enrichment in primary cilia of NIH 3T3 cells treated with Pur (5 μM) or SAG (1 μM) ± DAPT (18.75 μM). Arrows denote cilia shown in the insets, in which Smo and αTub are offset to show colocalization. Low- and high-magnification scale bars represent 10 and 1 μm.

(E) Box and whisker plots of Smo fluorescence in the cilia of NIH 3T3 cells treated with Pur or SAG ± DAPT. The black numbers indicate the number of cilia analyzed. The red numbers indicate the percentage of cilia with Smo. NS, $p > 0.05$.

(F–I) Ciliary enrichment of Ptch1 in *Ptch1*$^{-/-}$;*Ptch1-YFP* MEFs after exposure to DAPT (18.75 μM) with or without Shh (50 nM). Low- and high-magnification scale bars represent 10 and 1 μm.

(J) Box and whisker plots of Ptch1 fluorescence in the cilia of *Ptch1*$^{-/-}$;*Ptch1-YFP* MEFs. ***$p < 0.001$.

(K–N) Analysis of Smo localization in *Ptch1*$^{-/-}$ MEFs treated with or without Shh (50 nM) ± DAPT (18.75 μM). Arrows denote cilia shown in the insets, in which Smo and αTub channels are offset to show colocalization. Scale bars represent 10 and 1 μm (insets).

(O) Box and whisker plots of Smo fluorescence in the cilia of *Ptch1*$^{-/-}$ MEFs treated with or without Shh (50 nM) ± DAPT (18.75 μM). NS, $p > 0.05$.

(P–R) Apical Ptch1 staining in the ventral spinal cord of E11.5 embryos. The pMN and p3 labels were determined by serial section staining for Olig2 and Nkx2.2 (not shown). Insets show Ptch1 presence in Arl13b-stained primary cilia. Scale bars represent 20 and 1 μm (insets).

(S) Scatterplot of the mean intensity of apical Ptch1 staining in a 250 μm^2 area ± SEM. Each point represents the mean intensity from multiple sections collected from a single embryo. Each group is comprised of data from 6–12 embryos. The intensity of Ptch1 was normalized to littermate controls. *$p < 0.05$, **$p < 0.01$. See also Figure S8.

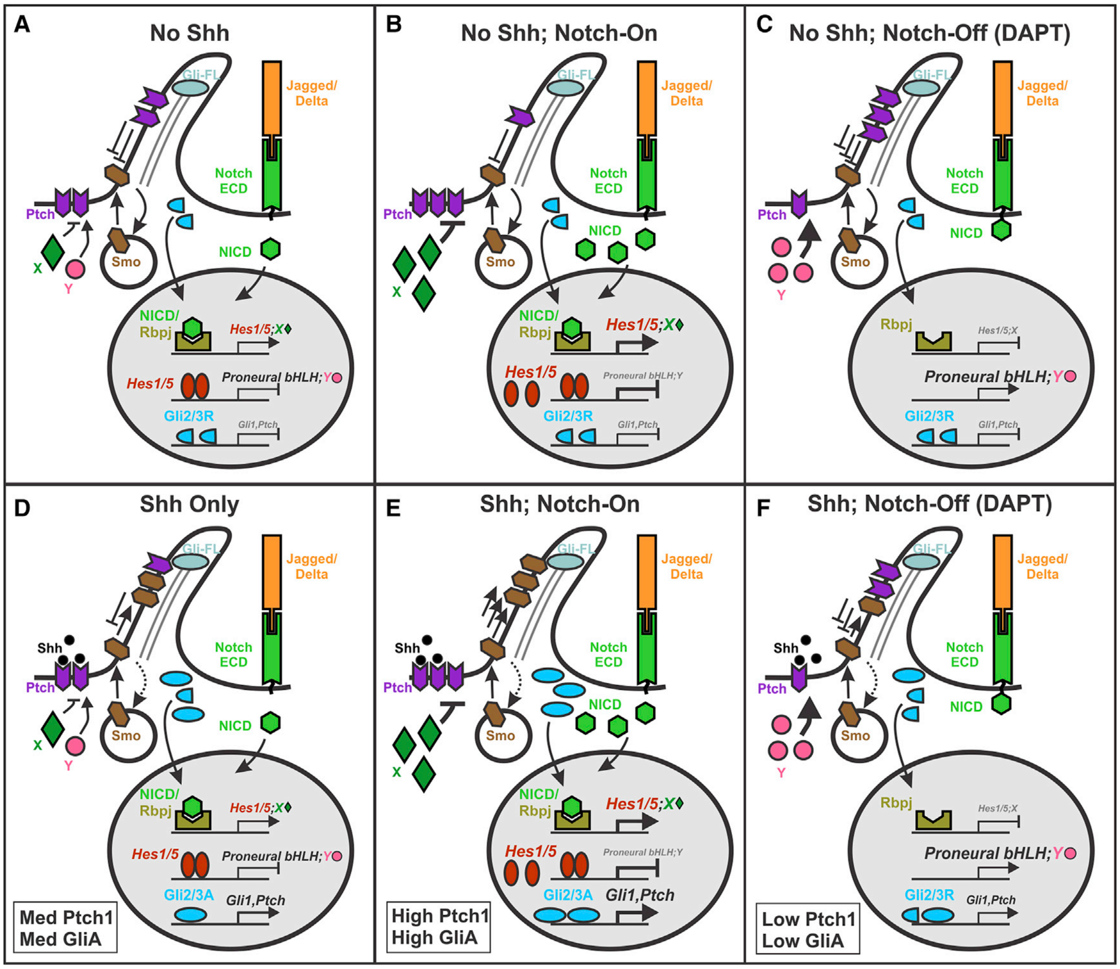

Figure 8. Models for Interactions between Notch and Shh Signaling

Models depicting how Notch signaling modulates cellular responses to Shh by regulating the movement of Ptch1 to primary cilia.

(A and D) In the absence of Shh, Ptch1 is present within and adjacent to primary cilia. Upon addition, Shh ligand binds to Ptch1, permitting Smo entry into the cilia where it stimulates Gli transcriptional activities. Direct downstream effectors of Notch signaling that promote Ptch1 clearance from primary cilia (X) and indirect effectors suppressed by Hes genes (Y) that increase Ptch1 ciliary accumulation are depicted.

(B and E) Notch activation via the ectopic expression of NICD reduces Ptch1 presence within primary cilia facilitating Smo entry and activation of Gli proteins.

(C and F) Notch inhibition, via the addition of DAPT or removal of Rbpj, elevates the presence of Ptch1 within primary cilia. Smo entry is impeded and Gli activities correspondingly reduced.

Olig2 expression and shifts in specific classes of neurons and glia. Importantly, multiple lines of evidence indicate that these changes are due to the ability of Notch to modulate how NPCs interpret the endogenous Shh signaling gradient rather than more direct effects on cell fate determination. First, all changes in NPC fates occurred within the context of Nkx6.1^{+} progenitors, which reflect the limit of endogenous Shh signaling in the spinal cord (Briscoe et al., 2000). Second, Notch manipulation in the intermediate spinal cord impacted NPC maintenance without any change in dorsoventral patterning or shift in glial cell types. Third, in fibroblasts, Notch activation and inactivation were unable to modulate Smo trafficking to primary cilia or Gli transcriptional activity without the coadministration of Shh ligand. Collectively, these data indicate that Notch plays a supporting role in tissue patterning by tuning the response of cells to Shh present in the developing embryo or culture media.

It has long been appreciated that the influences of Shh on neural fate selection are generally restricted to dividing cells (Ericson et al., 1996). Recent studies have provided molecular explanations for this relationship showing that most Shh/Gli-regulated genes are coregulated by SoxB1 transcription factors such as Sox2 that are broadly expressed by NPCs (Oosterveen et al., 2012; Peterson et al., 2012; Oosterveen et al., 2013). Some of the positive effects of Notch on Shh signaling could thus be accounted for by its ability to elevate SoxB1 levels as it maintains NPCs in an undifferentiated state. However, our data indicate that Notch can also act at a more proximal level, regulating the ciliary localization of at least two key components of the Shh transduction pathway, Ptch1 and Smo. Ptch1 appears to be the most directly impacted by Notch, as the addition of DAPT alone to fibroblasts promotes Ptch1 accumulation within primary cilia (Figures 7F, 7G, and 7J), and Ptch1 is known to block Smo

entry and downstream signaling events (Rohatgi et al., 2007). Moreover, DAPT was unable to reduce Smo accumulation within cilia in the absence of Ptch1 or in the presence of Pur and SAG, small molecules that bypass Ptch1 function (Figures 7A–7E and 7K–7O). These observations in fibroblasts also hold true for spinal cord NPCs, as *Rbpj* deletion increased Ptch1 protein in and around primary cilia, whereas NICD misexpression reduced it. These changes correspondingly impacted Smo presence in cilia and, ultimately, the expression of specific NPC fate determinants (Figures 7P–7S).

Notch as a Modulator of Ciliary Trafficking

How might Notch signaling alter Ptch1 and Smo trafficking? In epidermal cells, Notch receptors and processing enzymes are located in and adjacent to primary cilia, and ciliary transport is required for Notch pathway activity (Ezratty et al., 2011). Based on this proximity, Notch signaling components could conceivably impact the interactions of ciliary transport proteins with Shh signaling components. However, our results point to Notch acting through a transcriptional mechanism. First, changes in NPC fates and Gli transcriptional activity were seen with either removal of Rbpj function or increased expression of NICD, components whose main sites of action are known to be in the nucleus. Second, the Shh-potentiating activities seen with NICD misexpression were recapitulated by the forced expression of Hes1, one of the best known downstream transcriptional effectors of the Notch pathway. Third, the effects of DAPT administration on Ptch1 and Smo trafficking were not immediate, but rather required at least 12 hr of exposure—more than sufficient time for a transcriptionally mediated response. Finally, DAPT effects on Smo trafficking were blocked by the addition of the transcriptional inhibitor α-amanitin. Together, these results lead us to propose that Notch and Hes genes modulate Shh signaling by regulating the expression of genes whose products impact the trafficking of Ptch1, Smo, and potentially other Shh signaling components to primary cilia, designated as "X" for direct Notch effectors and "Y" for Hes-suppressed effectors (Figure 8).

While a great deal is known about the transcriptional control of *Ptch1* in response to Shh pathway activation, relatively little is known about the regulation of Ptch1 protein trafficking. Some insights into this process have been recently made by observations that Ptch1 exit from primary cilia requires the function of the intraflagellar transport (IFT) protein Ift25 (Keady et al., 2012), and endocytic turnover mediated by the ubiquitin E3 ligases Smurf1 and Smurf2 (Yue et al., 2014). Loss of these components results in Ptch1 accumulation within primary cilia and reduced cellular responses to Shh (Keady et al., 2012; Yue et al., 2014), reminiscent of the effects seen with the loss of Notch signaling. However, none of these genes were changed by our Notch manipulations (J.H.K. and B.G.N., unpublished data). A better understanding of the downstream targets of Notch and Hes1 should yield important new insights into how the localization and function of Ptch1 and other Shh signaling components may be controlled.

Is a Role for Notch Gating Responses to Other Developmental Signals Dependent on Cilia?

The primary cilium is a nonmotile organelle that is present on almost all vertebrate cells (Pazour and Witman, 2003). Although primary cilia were first observed over a century ago (Zimmermann, 1898), their function as an antenna-like organelle that allows cells to detect extracellular environmental stimuli and modulate an appropriate intracellular response has only recently been realized. In addition to Shh signaling, primary cilia are thought to be essential for Hippo, mTor, Notch, Pdgfrα, and Wnt signaling (Schneider et al., 2005; Boehlke et al., 2010; Ezratty et al., 2011; Habbig et al., 2011; Lancaster et al., 2011). The importance of primary cilia is perhaps best illustrated through ciliopathies, a group of genetic disorders that are due to defects in the generation or function of cilia, which collectively affect nearly every major organ in the human body (Novarino et al., 2011). As no protein synthesis occurs within the cilium, the formation of the cilium and the accumulation of signaling pathway components within the cilium are entirely dependent on the IFT system to shuttle proteins to their proper areas (Pedersen and Rosenbaum, 2008).

While our study focused on the impact of Notch on Shh signaling by altering the localization of Ptch1 and Smo, the mechanisms used to achieve this result are likely to have a broader impact on other signaling pathways that depend upon the IFT system. Consistent with this hypothesis, we have carried out a series of preliminary expression profiling experiments in NIH 3T3 cells, which indicate that DAPT addition reduces the expression of several proteins known to be associated with primary cilia (Ishikawa et al., 2012), including components of the Pdgfrα and Wnt signaling pathways, and various extracellular matrix proteins (J.H.K. and B.G.N., unpublished data). In this regard, the mechanism through which Notch gates the responsiveness of cells to Shh might signify a more general role for Notch modulating ciliary transport that could impact multiple signaling pathways involved in both development and disease.

EXPERIMENTAL PROCEDURES

Animal Preparation and Tissue Analysis

Olig2^{Cre} and *Dbx1*^{Cre} mice were generated as previously described (Bielle et al., 2005; Dessaud et al., 2007). Cre mice were crossed with *R26R*^{GFP} transgenic reporter mice (B6;129-*Gt(ROSA)26Sor*^{tm2Sho}/J; Jackson Labs Stock #004077) (Mao et al., 2001); *R26R*$^{NICD\text{-}nGFP}$ transgenic floxed mice (*Gt(ROSA)26Sor*$^{tm1(Notch1)Dam}$/J; Jackson Labs Stock #008159) (Murtaugh et al., 2003), or *Rbpj*^{CKO} mice (Han et al., 2002). *Olig2*$^{-/-}$, *Nkx2.2*$^{-/-}$, and *Pax6*$^{Sey/Sey}$ mutant mice were generated as previously described (Novitch et al., 2001; Rousso et al., 2012). All mice were maintained and tissue collected in accordance with guidelines set forth by the UCLA Institutional Animal Care and Use Committee. Chick neural plate explants were generated as previously described (Dessaud et al., 2007). All spinal cord tissues were fixed, cryoprotected, sectioned, and processed for immunohistochemistry or in situ hybridization as previously described (Novitch et al., 2001; Gaber et al., 2013). Antibodies and probes used are listed in the Supplemental Experimental Procedures.

Cell Culture and Primary Cilia Analysis

NIH 3T3 fibroblasts (CRL-1658) and C2C12 myoblasts (CRL-1772) were purchased from ATCC. Shh-LIGHT2 cells were used as previously described (Taipale et al., 2000). *Ptch1*$^{-/-}$ and *Ptch1*$^{-/-}$;Ptch1-YFP MEFs were generated as previously described (Rohatgi et al., 2007, 2009). Primitive human neuroepithelial progenitors were generated from embryonic stem cells as previously described (Hu et al., 2009). For cilia analysis in fibroblasts, cells were plated onto glass coverslips, grown to 80%–100% confluency in DMEM containing 10% bovine calf serum (BCS) and then changed to low serum media (0.5% BCS) at the beginning of experiments. Cells were fixed in 4% paraformaldehyde, incubated with indicated primary and secondary antibodies, and

mounted in Prolong Gold (Invitrogen). See also Supplemental Experimental Procedures.

Statistical Analyses

Unless otherwise stated, cell counts, luciferase assays, and qPCR analyses are presented as mean values ± SEM. For Figures 1Q, 2M, 2N, 3M, 3N, 5I, 6K, 6L, 7S, S3J, S3K, S4G, S4AF–S4AI, S7Q, S7R, and S8I, experimental conditions were compared with the control, and an ANOVA with a Dunnett's post hoc test was performed. For the data shown in Figures 4G, 4H, 5C, 5L, 5N, S5D, S5I–S5K, and S8H–S8J, unpaired, two-tailed t test were performed. All ciliary Smo fluorescence data sets did not pass the Shapiro-Wilk normality test. Thus, for all ciliary Smo analyses between two groups (Figures 5M, 7E, 7J, S8B, S8D, and S8F) two-tailed nonparametric Mann-Whitney tests were performed. For analyses between three or more groups (Figures 5J, 5K, 7O, S5A, S6D, S6H, and S6L), nonparametric Kruskal-Wallis tests were used along with Dunnett's post hoc tests. All statistical analyses were calculated using Graphpad Prism 6 software. Significance was assumed when $p < 0.05$.

SUPPLEMENTAL INFORMATION

Supplemental Information includes Supplemental Experimental Procedures and eight figures and can be found with this article online at http://dx.doi.org/10.1016/j.devcel.2015.03.005.

AUTHOR CONTRIBUTIONS

J.H.K., L.Y., E.D., K.C., and D.M.M. performed the experiments. R.R. contributed vital reagents and insights. J.H.K., L.Y., J.B., and B.G.N. designed the experiments and wrote the paper.

ACKNOWLEDGMENTS

We thank S. Butler, P. Niewiadomski, H. Kornblum, and G. Weinmaster for helpful discussions and comments on the manuscript; T. Honjo, S. Morrison, C. Murtaugh, and A. Pierani for mice; and L. Cheng, T. Jessell, Y. Nakagawa, K. Phan, S. Pfaff, and T. Sudo for reagents. We also thank K. Dale for communications before submission of the manuscript. This work was supported by the UCLA Broad Center for Regenerative Medicine and Stem Cell Research, the Rose Hills Foundation, and grants to B.G.N. from the March of Dimes Foundation (6-FY10-296) and the NINDS (NS053976 and NS072804). J.H.K. was also supported by a UCLA Dissertation Year Fellowship. R.R. was supported by grants from the NIGMS (DP2GM10544) and the March of Dimes Foundation (6-FY13-104). E.D. and J.B. were supported by the Medical Research Council (UK) (U117560541) and the Wellcome Trust (WT098326MA).

Received: September 11, 2014
Revised: December 29, 2014
Accepted: March 3, 2015
Published: April 30, 2015

REFERENCES

Balaskas, N., Ribeiro, A., Panovska, J., Dessaud, E., Sasai, N., Page, K.M., Briscoe, J., and Ribes, V. (2012). Gene regulatory logic for reading the Sonic Hedgehog signaling gradient in the vertebrate neural tube. Cell *148*, 273–284.

Bielle, F., Griveau, A., Narboux-Nême, N., Vigneau, S., Sigrist, M., Arber, S., Wassef, M., and Pierani, A. (2005). Multiple origins of Cajal-Retzius cells at the borders of the developing pallium. Nat. Neurosci. *8*, 1002–1012.

Boehlke, C., Kotsis, F., Patel, V., Braeg, S., Voelker, H., Bredt, S., Beyer, T., Janusch, H., Hamann, C., Gödel, M., et al. (2010). Primary cilia regulate mTORC1 activity and cell size through Lkb1. Nat. Cell Biol. *12*, 1115–1122.

Briscoe, J., and Novitch, B.G. (2008). Regulatory pathways linking progenitor patterning, cell fates and neurogenesis in the ventral neural tube. Philos. Trans. R. Soc. Lond. B Biol. Sci. *363*, 57–70.

Briscoe, J., and Thérond, P.P. (2013). The mechanisms of Hedgehog signalling and its roles in development and disease. Nat. Rev. Mol. Cell Biol. *14*, 416–429.

Briscoe, J., Pierani, A., Jessell, T.M., and Ericson, J. (2000). A homeodomain protein code specifies progenitor cell identity and neuronal fate in the ventral neural tube. Cell *101*, 435–445.

Butler, S.J., and Bronner, M.E. (2015). From classical to current: Analyzing peripheral nervous system and spinal cord lineage and fate. Dev. Biol. *398*, 135–146.

Chen, J.K., Taipale, J., Young, K.E., Maiti, T., and Beachy, P.A. (2002). Small molecule modulation of Smoothened activity. Proc. Natl. Acad. Sci. USA *99*, 14071–14076.

Corbit, K.C., Aanstad, P., Singla, V., Norman, A.R., Stainier, D.Y., and Reiter, J.F. (2005). Vertebrate Smoothened functions at the primary cilium. Nature *437*, 1018–1021.

Dessaud, E., Yang, L.L., Hill, K., Cox, B., Ulloa, F., Ribeiro, A., Mynett, A., Novitch, B.G., and Briscoe, J. (2007). Interpretation of the sonic hedgehog morphogen gradient by a temporal adaptation mechanism. Nature *450*, 717–720.

Dessaud, E., McMahon, A.P., and Briscoe, J. (2008). Pattern formation in the vertebrate neural tube: a sonic hedgehog morphogen-regulated transcriptional network. Development *135*, 2489–2503.

Dessaud, E., Ribes, V., Balaskas, N., Yang, L.L., Pierani, A., Kicheva, A., Novitch, B.G., Briscoe, J., and Sasai, N. (2010). Dynamic assignment and maintenance of positional identity in the ventral neural tube by the morphogen sonic hedgehog. PLoS Biol. *8*, e1000382.

Dovey, H.F., John, V., Anderson, J.P., Chen, L.Z., de Saint Andrieu, P., Fang, L.Y., Freedman, S.B., Folmer, B., Goldbach, E., Holsztynska, E.J., et al. (2001). Functional gamma-secretase inhibitors reduce beta-amyloid peptide levels in brain. J. Neurochem. *76*, 173–181.

Eggenschwiler, J.T., and Anderson, K.V. (2007). Cilia and developmental signaling. Annu. Rev. Cell Dev. Biol. *23*, 345–373.

Ericson, J., Morton, S., Kawakami, A., Roelink, H., and Jessell, T.M. (1996). Two critical periods of Sonic Hedgehog signaling required for the specification of motor neuron identity. Cell *87*, 661–673.

Ezratty, E.J., Stokes, N., Chai, S., Shah, A.S., Williams, S.E., and Fuchs, E. (2011). A role for the primary cilium in Notch signaling and epidermal differentiation during skin development. Cell *145*, 1129–1141.

Fuccillo, M., Joyner, A.L., and Fishell, G. (2006). Morphogen to mitogen: the multiple roles of hedgehog signalling in vertebrate neural development. Nat. Rev. Neurosci. *7*, 772–783.

Furukawa, T., Mukherjee, S., Bao, Z.Z., Morrow, E.M., and Cepko, C.L. (2000). rax, Hes1, and notch1 promote the formation of Müller glia by postnatal retinal progenitor cells. Neuron *26*, 383–394.

Gaber, Z.B., Butler, S.J., and Novitch, B.G. (2013). PLZF regulates fibroblast growth factor responsiveness and maintenance of neural progenitors. PLoS Biol. *11*, e1001676.

Gaiano, N., and Fishell, G. (2002). The role of notch in promoting glial and neural stem cell fates. Annu. Rev. Neurosci. *25*, 471–490.

Gaiano, N., Nye, J.S., and Fishell, G. (2000). Radial glial identity is promoted by Notch1 signaling in the murine forebrain. Neuron *26*, 395–404.

Ge, W., Martinowich, K., Wu, X., He, F., Miyamoto, A., Fan, G., Weinmaster, G., and Sun, Y.E. (2002). Notch signaling promotes astrogliogenesis via direct CSL-mediated glial gene activation. J. Neurosci. Res. *69*, 848–860.

Geling, A., Steiner, H., Willem, M., Bally-Cuif, L., and Haass, C. (2002). A gamma-secretase inhibitor blocks Notch signaling in vivo and causes a severe neurogenic phenotype in zebrafish. EMBO Rep. *3*, 688–694.

Habbig, S., Bartram, M.P., Müller, R.U., Schwarz, R., Andriopoulos, N., Chen, S., Sägmüller, J.G., Hoehne, M., Burst, V., Liebau, M.C., et al. (2011). NPHP4, a cilia-associated protein, negatively regulates the Hippo pathway. J. Cell Biol. *193*, 633–642.

Han, H., Tanigaki, K., Yamamoto, N., Kuroda, K., Yoshimoto, M., Nakahata, T., Ikuta, K., and Honjo, T. (2002). Inducible gene knockout of transcription factor recombination signal binding protein-J reveals its essential role in T versus B lineage decision. Int. Immunol. *14*, 637–645.

Hatakeyama, J., Bessho, Y., Katoh, K., Ookawara, S., Fujioka, M., Guillemot, F., and Kageyama, R. (2004). Hes genes regulate size, shape and histogenesis

of the nervous system by control of the timing of neural stem cell differentiation. Development *131*, 5539–5550.

Hochstim, C., Deneen, B., Lukaszewicz, A., Zhou, Q., and Anderson, D.J. (2008). Identification of positionally distinct astrocyte subtypes whose identities are specified by a homeodomain code. Cell *133*, 510–522.

Hu, B.Y., Du, Z.W., and Zhang, S.C. (2009). Differentiation of human oligodendrocytes from pluripotent stem cells. Nat. Protoc. *4*, 1614–1622.

Huang, P., Xiong, F., Megason, S.G., and Schier, A.F. (2012). Attenuation of Notch and Hedgehog signaling is required for fate specification in the spinal cord. PLoS Genet. *8*, e1002762.

Ingram, W.J., McCue, K.I., Tran, T.H., Hallahan, A.R., and Wainwright, B.J. (2008). Sonic Hedgehog regulates Hes1 through a novel mechanism that is independent of canonical Notch pathway signalling. Oncogene *27*, 1489–1500.

Ishikawa, H., Thompson, J., Yates, J.R., 3rd, and Marshall, W.F. (2012). Proteomic analysis of mammalian primary cilia. Curr. Biol. *22*, 414–419.

Kageyama, R., Ohtsuka, T., and Kobayashi, T. (2007). The Hes gene family: repressors and oscillators that orchestrate embryogenesis. Development *134*, 1243–1251.

Kageyama, R., Ohtsuka, T., Shimojo, H., and Imayoshi, I. (2009). Dynamic regulation of Notch signaling in neural progenitor cells. Curr. Opin. Cell Biol. *21*, 733–740.

Keady, B.T., Samtani, R., Tobita, K., Tsuchya, M., San Agustin, J.T., Follit, J.A., Jonassen, J.A., Subramanian, R., Lo, C.W., and Pazour, G.J. (2012). IFT25 links the signal-dependent movement of Hedgehog components to intraflagellar transport. Dev. Cell *22*, 940–951.

Kutejova, E., Briscoe, J., and Kicheva, A. (2009). Temporal dynamics of patterning by morphogen gradients. Curr. Opin. Genet. Dev. *19*, 315–322.

Lancaster, M.A., Schroth, J., and Gleeson, J.G. (2011). Subcellular spatial regulation of canonical Wnt signalling at the primary cilium. Nat. Cell Biol. *13*, 700–707.

Le Dréau, G., and Martí, E. (2013). The multiple activities of BMPs during spinal cord development. Cell. Mol. Life Sci. *70*, 4293–4305.

Mao, X., Fujiwara, Y., Chapdelaine, A., Yang, H., and Orkin, S.H. (2001). Activation of EGFP expression by Cre-mediated excision in a new ROSA26 reporter mouse strain. Blood *97*, 324–326.

Mizuguchi, R., Sugimori, M., Takebayashi, H., Kosako, H., Nagao, M., Yoshida, S., Nabeshima, Y., Shimamura, K., and Nakafuku, M. (2001). Combinatorial roles of olig2 and neurogenin2 in the coordinated induction of pan-neuronal and subtype-specific properties of motoneurons. Neuron *31*, 757–771.

Moellering, R.E., Cornejo, M., Davis, T.N., Del Bianco, C., Aster, J.C., Blacklow, S.C., Kung, A.L., Gilliland, D.G., Verdine, G.L., and Bradner, J.E. (2009). Direct inhibition of the NOTCH transcription factor complex. Nature *462*, 182–188.

Muroyama, Y., Fujiwara, Y., Orkin, S.H., and Rowitch, D.H. (2005). Specification of astrocytes by bHLH protein SCL in a restricted region of the neural tube. Nature *438*, 360–363.

Murtaugh, L.C., Stanger, B.Z., Kwan, K.M., and Melton, D.A. (2003). Notch signaling controls multiple steps of pancreatic differentiation. Proc. Natl. Acad. Sci. USA *100*, 14920–14925.

Novarino, G., Akizu, N., and Gleeson, J.G. (2011). Modeling human disease in humans: the ciliopathies. Cell *147*, 70–79.

Novitch, B.G., Chen, A.I., and Jessell, T.M. (2001). Coordinate regulation of motor neuron subtype identity and pan-neuronal properties by the bHLH repressor Olig2. Neuron *31*, 773–789.

Oosterveen, T., Kurdija, S., Alekseenko, Z., Uhde, C.W., Bergsland, M., Sandberg, M., Andersson, E., Dias, J.M., Muhr, J., and Ericson, J. (2012). Mechanistic differences in the transcriptional interpretation of local and long-range Shh morphogen signaling. Dev. Cell *23*, 1006–1019.

Oosterveen, T., Kurdija, S., Enserö, M., Uhde, C.W., Bergsland, M., Sandberg, M., Sandberg, R., Muhr, J., and Ericson, J. (2013). SoxB1-driven transcriptional network underlies neural-specific interpretation of morphogen signals. Proc. Natl. Acad. Sci. USA *110*, 7330–7335.

Pazour, G.J., and Witman, G.B. (2003). The vertebrate primary cilium is a sensory organelle. Curr. Opin. Cell Biol. *15*, 105–110.

Pedersen, L.B., and Rosenbaum, J.L. (2008). Intraflagellar transport (IFT) role in ciliary assembly, resorption and signalling. Curr. Top. Dev. Biol. *85*, 23–61.

Peterson, K.A., Nishi, Y., Ma, W., Vedenko, A., Shokri, L., Zhang, X., McFarlane, M., Baizabal, J.M., Junker, J.P., van Oudenaarden, A., et al. (2012). Neural-specific Sox2 input and differential Gli-binding affinity provide context and positional information in Shh-directed neural patterning. Genes Dev. *26*, 2802–2816.

Petit, A., Bihel, F., Alvès da Costa, C., Pourquié, O., Checler, F., and Kraus, J.L. (2001). New protease inhibitors prevent gamma-secretase-mediated production of Abeta40/42 without affecting Notch cleavage. Nat. Cell Biol. *3*, 507–511.

Pierfelice, T., Alberi, L., and Gaiano, N. (2011). Notch in the vertebrate nervous system: an old dog with new tricks. Neuron *69*, 840–855.

Ribes, V., and Briscoe, J. (2009). Establishing and interpreting graded Sonic Hedgehog signaling during vertebrate neural tube patterning: the role of negative feedback. Cold Spring Harb. Perspect. Biol. *1*, a002014.

Rogers, K.W., and Schier, A.F. (2011). Morphogen gradients: from generation to interpretation. Annu. Rev. Cell Dev. Biol. *27*, 377–407.

Rohatgi, R., Milenkovic, L., and Scott, M.P. (2007). Patched1 regulates hedgehog signaling at the primary cilium. Science *317*, 372–376.

Rohatgi, R., Milenkovic, L., Corcoran, R.B., and Scott, M.P. (2009). Hedgehog signal transduction by Smoothened: pharmacologic evidence for a 2-step activation process. Proc. Natl. Acad. Sci. USA *106*, 3196–3201.

Rousso, D.L., Gaber, Z.B., Wellik, D., Morrisey, E.E., and Novitch, B.G. (2008). Coordinated actions of the forkhead protein Foxp1 and Hox proteins in the columnar organization of spinal motor neurons. Neuron *59*, 226–240.

Rousso, D.L., Pearson, C.A., Gaber, Z.B., Miquelajauregui, A., Li, S., Portera-Cailliau, C., Morrisey, E.E., and Novitch, B.G. (2012). Foxp-mediated suppression of N-cadherin regulates neuroepithelial character and progenitor maintenance in the CNS. Neuron *74*, 314–330.

Rowitch, D.H., and Kriegstein, A.R. (2010). Developmental genetics of vertebrate glial-cell specification. Nature *468*, 214–222.

Scheer, N., Groth, A., Hans, S., and Campos-Ortega, J.A. (2001). An instructive function for Notch in promoting gliogenesis in the zebrafish retina. Development *128*, 1099–1107.

Schneider, L., Clement, C.A., Teilmann, S.C., Pazour, G.J., Hoffmann, E.K., Satir, P., and Christensen, S.T. (2005). PDGFRalphaalpha signaling is regulated through the primary cilium in fibroblasts. Curr. Biol. *15*, 1861–1866.

Sinha, S., and Chen, J.K. (2006). Purmorphamine activates the Hedgehog pathway by targeting Smoothened. Nat. Chem. Biol. *2*, 29–30.

Small, D., Kovalenko, D., Soldi, R., Mandinova, A., Kolev, V., Trifonova, R., Bagala, C., Kacer, D., Battelli, C., Liaw, L., et al. (2003). Notch activation suppresses fibroblast growth factor-dependent cellular transformation. J. Biol. Chem. *278*, 16405–16413.

Stamataki, D., Ulloa, F., Tsoni, S.V., Mynett, A., and Briscoe, J. (2005). A gradient of Gli activity mediates graded Sonic Hedgehog signaling in the neural tube. Genes Dev. *19*, 626–641.

Sun, T., Dong, H., Wu, L., Kane, M., Rowitch, D.H., and Stiles, C.D. (2003). Cross-repressive interaction of the Olig2 and Nkx2.2 transcription factors in developing neural tube associated with formation of a specific physical complex. J. Neurosci. *23*, 9547–9556.

Taipale, J., Chen, J.K., Cooper, M.K., Wang, B., Mann, R.K., Milenkovic, L., Scott, M.P., and Beachy, P.A. (2000). Effects of oncogenic mutations in Smoothened and Patched can be reversed by cyclopamine. Nature *406*, 1005–1009.

Taylor, M.K., Yeager, K., and Morrison, S.J. (2007). Physiological Notch signaling promotes gliogenesis in the developing peripheral and central nervous systems. Development *134*, 2435–2447.

Tsuchida, T., Ensini, M., Morton, S.B., Baldassare, M., Edlund, T., Jessell, T.M., and Pfaff, S.L. (1994). Topographic organization of embryonic motor neurons defined by expression of LIM homeobox genes. Cell *79*, 957–970.

Tukachinsky, H., Lopez, L.V., and Salic, A. (2010). A mechanism for vertebrate Hedgehog signaling: recruitment to cilia and dissociation of SuFu-Gli protein complexes. J. Cell Biol. *191*, 415–428.

Wall, D.S., Mears, A.J., McNeill, B., Mazerolle, C., Thurig, S., Wang, Y., Kageyama, R., and Wallace, V.A. (2009). Progenitor cell proliferation in the retina is dependent on Notch-independent Sonic hedgehog/Hes1 activity. J. Cell Biol. *184*, 101–112.

Wang, S., Sdrulla, A.D., diSibio, G., Bush, G., Nofziger, D., Hicks, C., Weinmaster, G., and Barres, B.A. (1998). Notch receptor activation inhibits oligodendrocyte differentiation. Neuron *21*, 63–75.

Yue, S., Tang, L.Y., Tang, Y., Tang, Y., Shen, Q.H., Ding, J., Chen, Y., Zhang, Z., Yu, T.T., Zhang, Y.E., and Cheng, S.Y. (2014). Requirement of Smurf-mediated endocytosis of Patched1 in sonic hedgehog signal reception. eLife *3*, e02555.

Zimmermann, K.W. (1898). Beiträge zur Kenntniss einiger Drüsen und Epithelien. Arch. Mikr. Anat. *52*, 552–706.

Developmental Cell
Supplemental Information

Notch Activity Modulates the Responsiveness of Neural Progenitors to Sonic Hedgehog Signaling

Jennifer H. Kong, Linlin Yang, Eric Dessaud, Katherine Chuang, Destaye M. Moore, Rajat Rohatgi, James Briscoe, and Bennett G. Novitch

Figure S1. Temporal and spatial documentation of *Olig2^{Cre}*-mediated recombination (related to Figure 1).

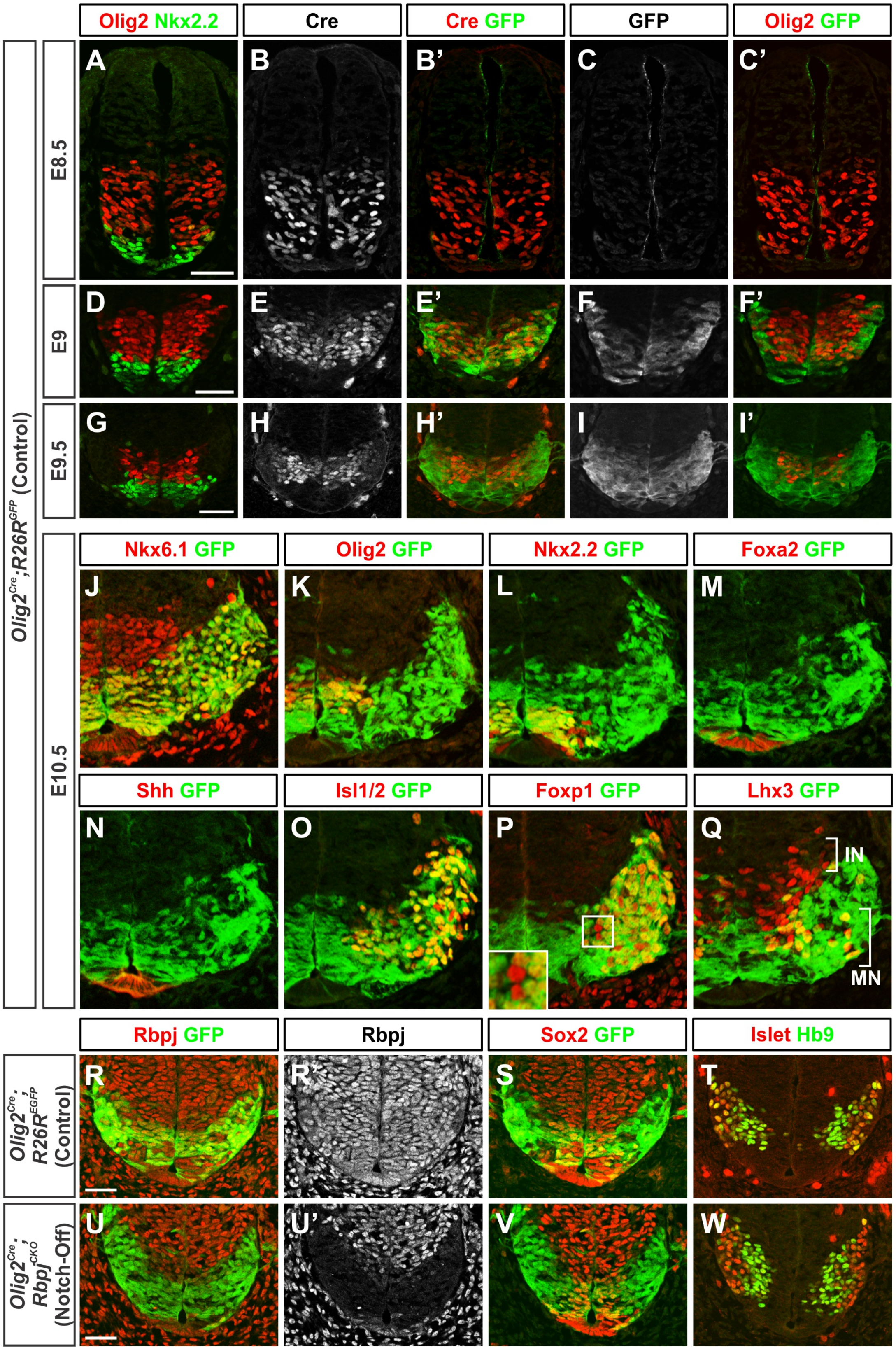

(A-I) Serial transverse spinal cord sections from E8.5-E9.5 Control (*Olig2^{Cre}; R26R^{GFP}*) embryos. Tissue was immunostained with Nkx2.2, Olig2, Cre, and GFP antibodies to assess where and when recombination occurred. Scale bars = 50 μm.
(J-Q) Analysis of E10.5 *Olig2^{Cre}; R26R^{GFP}* Control embryos shows that Cre recombination takes place in both the pMN (Nkx6.1^{+}/Olig2^{+}) and p3 (Nkx6.1^{+}/Nxk2.2^{+}) domains, but not in the floor plate (FoxA2^{+} or Shh^{+}). At this time point, Cre recombination had occurred in nearly all MNs (Isl1/2^{+}), including both LMC (FoxP1^{+}) and MMC (Lhx3^{+}) subgroups. Inset in (P) reveals a small population of non-recombined MNs. Note that Lhx3 is also prominently expressed by newborn V2 interneurons that form above the motor columns.
(R-W) Notch-Off embryos display an absence of Rbpj protein from pMN, p3 progenitors, and differentiated (Islet^{+}/Hb9^{+}) MNs starting around E9.5. Note that Sox2 is maintained in pMN/p3 cells despite the loss of Rbpj. Scale bars = 50 μm.

Figure S2. Notch signaling is activated in Notch-On embryos and reduced in Notch-Off embryos without any major disruptions to the neuroepithelial organization (related to Figures 1 and 2).

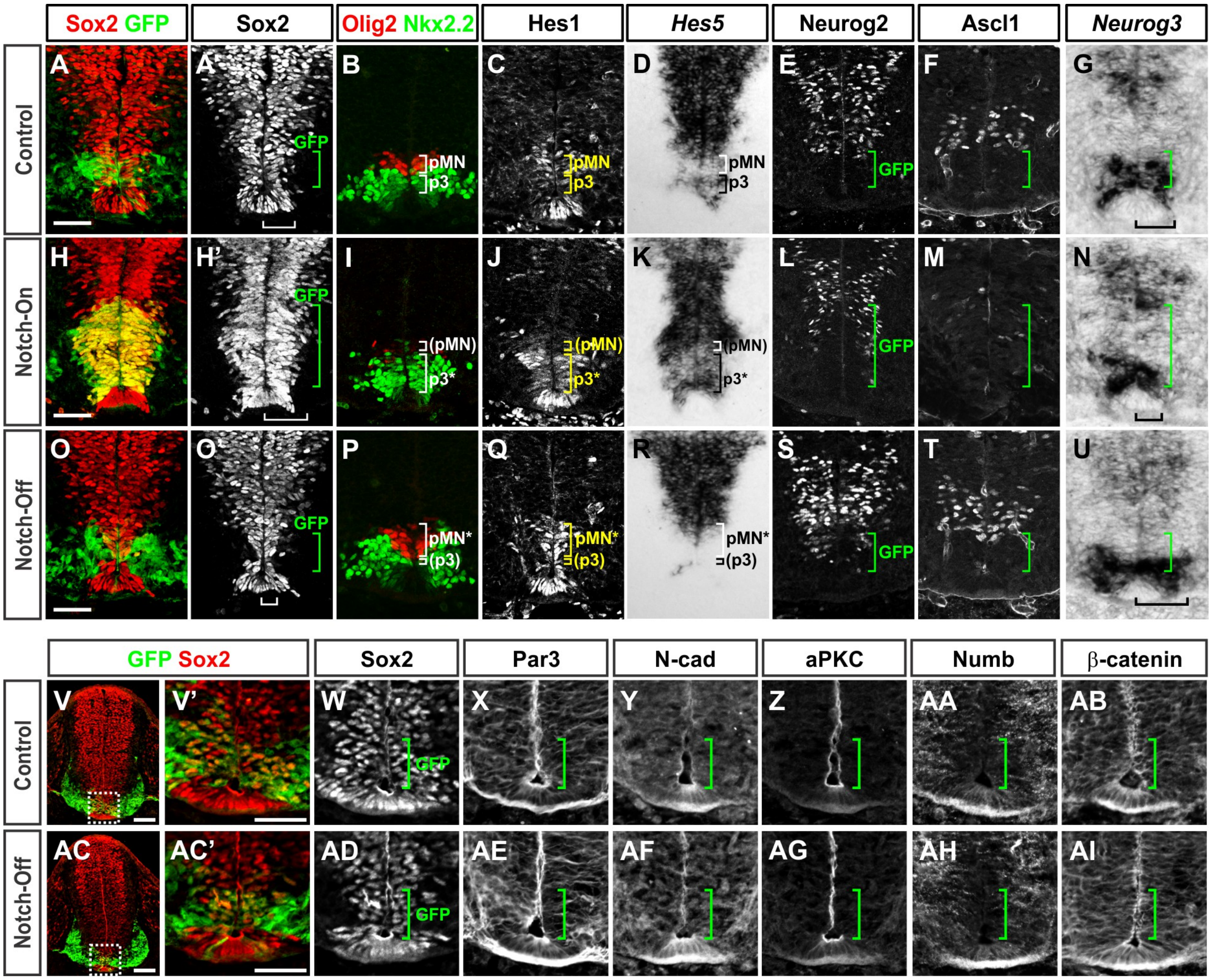

(A-U) Analysis of Notch signaling activity in Control (A-G), Notch-On (H-N), and Notch-Off (O-U) E11.5 embryos. (A, H, O) Green brackets demarcate the dorsoventral extent of the GFP^+ region of recombination and white brackets indicate the thickness of the $Sox2^+$ ventricular zone (VZ) within this same region. As predicted, NICD misexpression (Notch-On) expands the thickness of the $Sox2^+$ VZ, increases expression of canonical downstream Notch effectors (Hes1 and *Hes5*), and reduces expression of proneural transcription factors (Neurog2, Ascl1, and *Neurog3*). Conversely, Rbpj deletion (Notch-Off) has the opposite effect. Note, however, that Sox2 and Hes1 expression are maintained in the ventral spinal cord despite the removal of Rbpj (panels O and Q). Scale bars = 50 μm.

(V-AI) The neuroepithelial architecture and apicobasal polarity of neural progenitors is preserved in the ventral spinal cord of Notch-Off (AC-AI) E11.5 embryos. Within the region of recombination (marked by the green brackets), the lasting integrity of the ventricular zone is apparent through the maintenance of $Sox2^+$ progenitors and the continued presence of various cell polarity components (Par3, aPKC, and Numb) and cell adhesion molecules (N-cadherin and β-catenin). Scale bars = 100 μm.

Figure S3. The effect of Notch signaling manipulations on MN and glial cell fates (related to Figure 3).

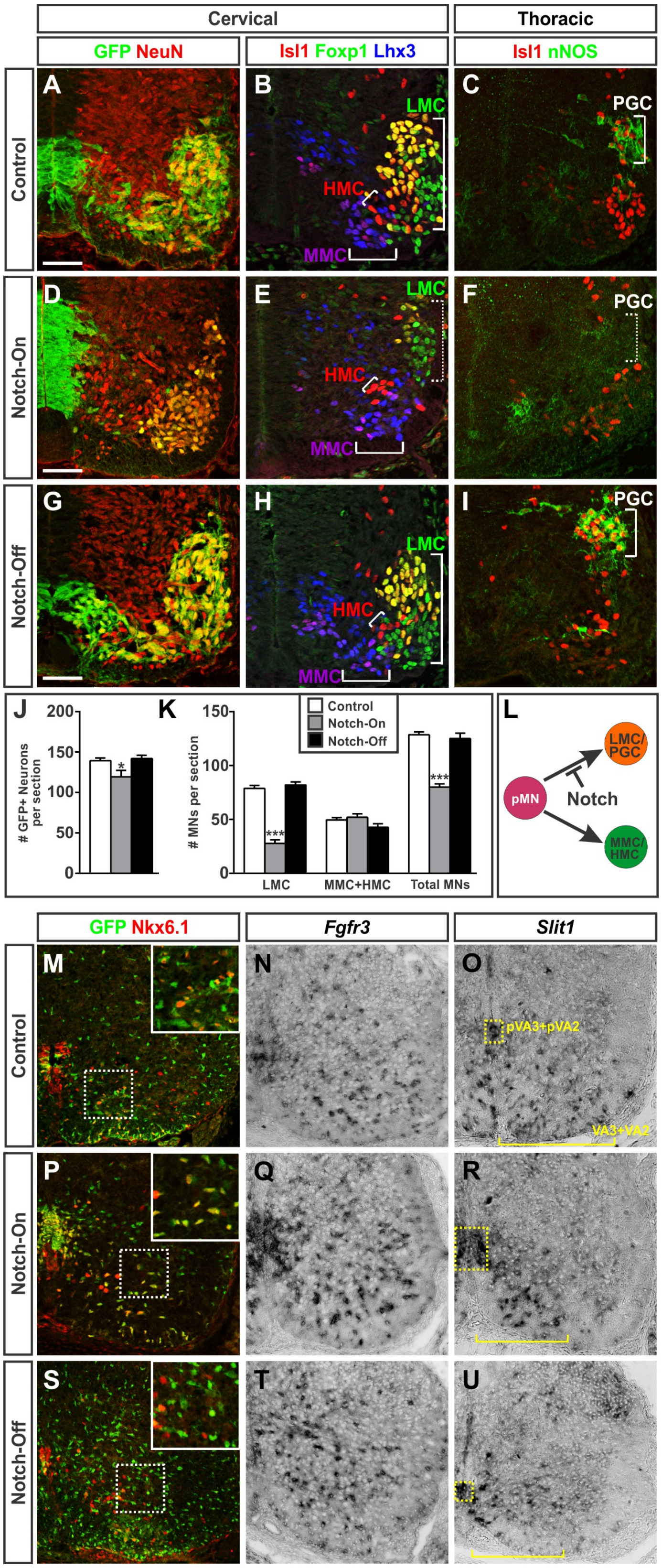

(A-I) In E11.5 embryos, *Olig2*^{Cre}-derived neurons (GFP^{+}/NeuN^{+}) include LMC (FoxP1^{+}), HMC (Isl1^{+}/FoxP1^{-}/Lhx3^{-}), MMC (Isl1^{+}/FoxP1^{-}/Lhx3^{+}), and PGC (Isl1^{+}/nNOS^{+}) MNs. Scale bars = 50 µm.
(J) Quantification of the total number of recombined neurons (GFP^{+}/NeuN^{+}) per spinal cord half. Plots show the mean ± SEM from multiple sections collected from 11-13 embryos from each experimental condition. NS, $p > 0.05$ and *$p < 0.05$.
(K) Quantification of the total number of MNs (Isl1^{+} and/or Hb9^{+}), LMC MNs (FoxP1^{+}), and MMC plus HMC MNs (Foxp1^{-} Isl1^{+} Lhx3^{+} and Foxp1^{-} Isl1^{+} Lhx3^{-}) per spinal cord half. In this analysis, all MNs were counted regardless of their GFP expression. Plots show the mean ± SEM from multiple sections collected from 8-30 embryos from each experimental condition. NS, $p > 0.05$ and ***$p < 0.001$.
(L) Summary of the role of Notch signaling in directing MN cell fates.
(M-U) Analysis of the impact of Notch manipulations on glial cell fates at E18.5.
(M-O) In Control embryos, *Olig2*^{Cre}-derivatives marked by GFP expression include pVA3 astrocyte progenitors and differentiated VA3 astrocytes that express Nkx6.1 (Hochstim et al, 2008). Serial section analysis reveals the presence of *Fgfr3*, which is expressed by multiple groups of astrocyte progenitors, and *Slit1*, which is selectively expressed by VA3 progenitors and differentiated astrocytes (Hochstim et al, 2008).
(P-U) Notch activation increases while Notch inactivation decreases the production of cells expressing the pVA3 and VA3 markers Nkx6.1, *Fgfr3*, and *Slit1*.

Figure S4. Manipulation of Notch signaling alters neuronal differentiation and progenitor maintenance in the intermediate spinal cord without overt changes in dorsoventral patterning or glial identities (related to Figures 2 and 3)

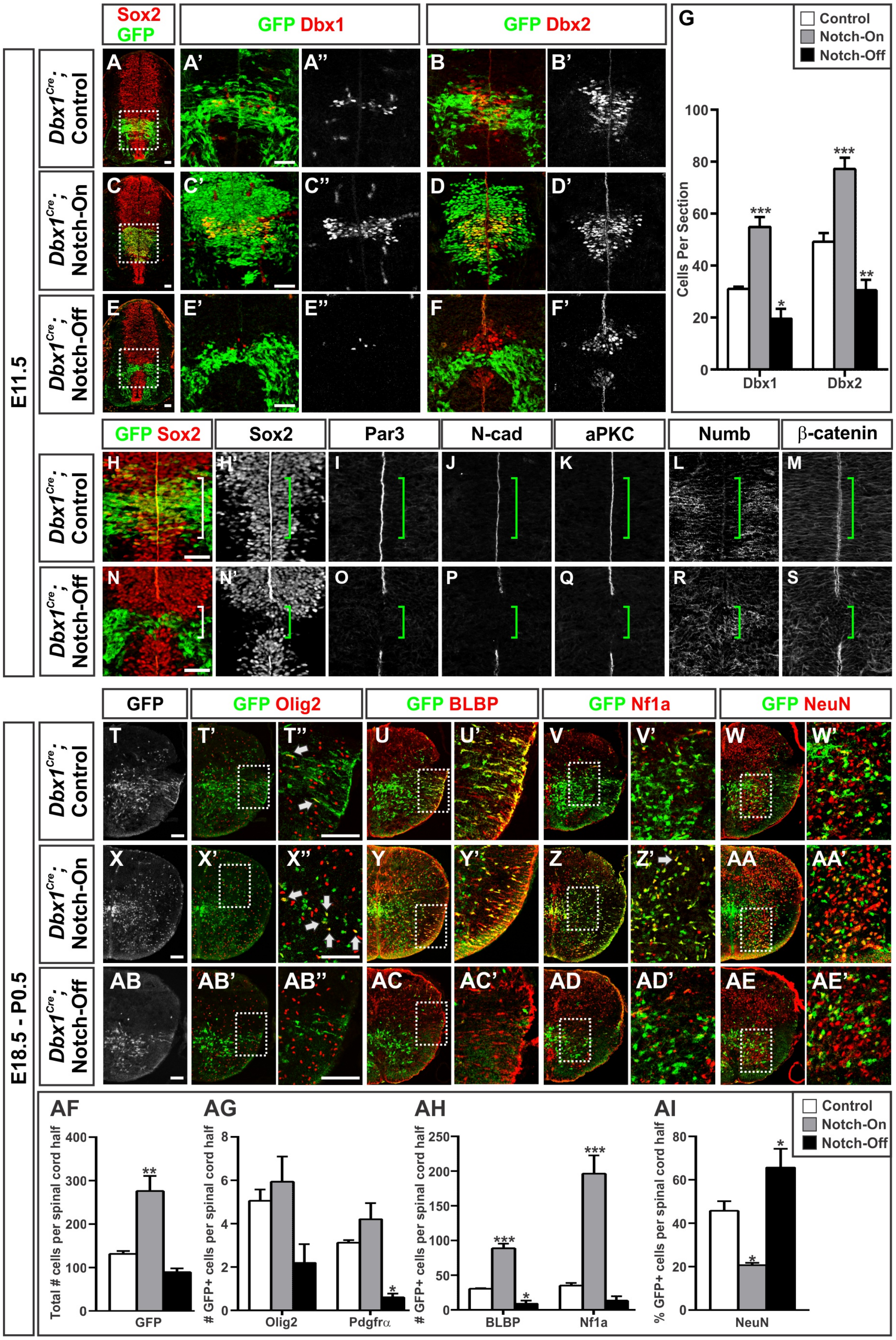

(A-F) In E11.5 control embryos, *Dbx1*^{Cre}-mediated recombination occurs within p0 (Dbx1^{+} Dbx2^{+}) and p1 (Dbx1^{-} Dbx2^{+}) progenitors. Notch activation increases while Notch inactivation decreases the formation of these cells. Scale bars = 50 µm.
(G) Quantification of the total number of Dbx1^{+} and Dbx2^{+} progenitors present in E11.5 Control, Notch-On, and Notch-Off embryos. For this analysis, progenitors were counted regardless of their GFP expression. Plots represent mean cell counts ± SEM from multiple sections collected from 6-8 embryos from each transgenic line. *$p < 0.05$, **$p < 0.01$, ***$p < 0.001$.
(H-S) The neuroepithelial architecture and apicobasal polarity of neural progenitors is greatly disrupted in the intermediate spinal cord of E11.5 *Dbx1*^{Cre}; Notch-Off embryos (N-S). Within the region of recombination, this disruption can be observed through the complete loss of Sox2^{+} progenitors, cell polarity components (Par3 and aPKC), and cell-to-cell adhesion molecules (N-cadherin and β-catenin). Green brackets demarcate the region of recombination. Scale bars = 100 µm.
(T-AE) In E18.5 Control embryos and P0.5 neonates, GFP^{+} Dbx1^{Cre}-derivatives include astrocyte precursors and astrocytes (BLBP^{+}/Nf1a^{+}), differentiated neurons (NeuN^{+}), but only a small number of oligodendrocyte precursors (OLPs) (Olig2^{+} or Pdgfrα^{+}). Notch activation increases the formation of astrocyte progenitors and astrocytes while at the same time reducing neurogenesis. Notch inactivation has the opposite effect. In contrast to *Olig2*$^{Cre-}$mediated manipulations, *Dbx1*^{Cre}-mediated Notch activation does not show any suppressive effect on oligodendrocyte formation. Likewise, *Dbx1*^{Cre}-mediated Notch inactivation does not lead to the formation of ectopic oligodendrocyte progenitors. Scale bars = 100 µm.
(AF-AI) Quantification of the total number of GFP^{+} cells per spinal cord half, GFP^{+} OLPs (GFP^{+}/Olig2^{+} and GFP^{+}/Pdgfrα^{+}), astrocyte precursors and astrocytes (GFP^{+}/BLBP^{+} and GFP^{+}/Nf1a^{+}), and neurons (GFP^{+}/NeuN^{+}). Plots display the mean ± SEM from multiple sections collected from 4-5 embryos for each experimental condition. *$p < 0.05$, **$p < 0.01$, ***$p < 0.001$.

Figure S5. Inhibition of Notch signaling reduces Smo trafficking to the primary cilia of NIH-3T3 fibroblasts with no disruption to apicobasal polarity (related to Figure 5).

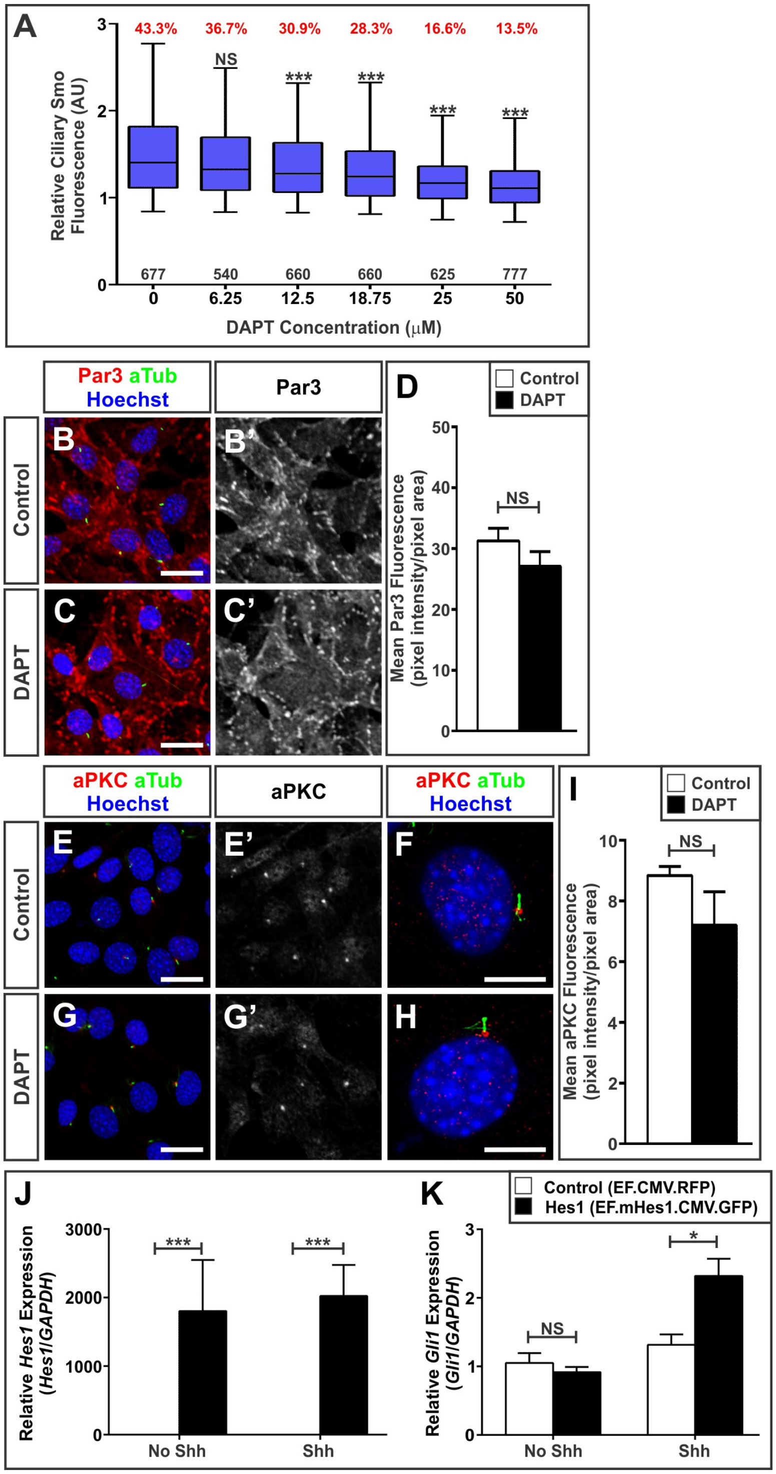

(A) Box and whisker plots of Smo fluorescence in the cilia of NIH-3T3 cells cultured in the presence of 50 nM Shh and various doses of DAPT (0-50 μM). The box extends from the 25th to 75th percentile, the line through the box represents the median, and the whiskers encompass

the 5th to 95th percentile. The number of cilia analyzed is indicated in black below the box plots and the percentage of Smo^+ cilia indicated in red above the box plots. DAPT reduces Smo presence within the primary cilia in a dose-dependent manner. For statistical analyses, all DAPT doses were compared to the Control (DMSO) group. NS, $p > 0.05$ and *** $p < 0.001$.
(B-C) Par3 immunofluorescence analysis of Control (DMSO) and DAPT (18.75 μM) treated NIH-3T3 cells. Scale bars = 20 μm.
(D) Quantification of the mean intensity ± SEM of Par3 fluorescence in a 320 μm x 320 μm area.
(E-H) aPKC immunofluorescence analysis of Control (DMSO) and DAPT (18.75 μM) treated NIH-3T3 cells. Low mag scale bars = 20 μm. High mag scale bars = 10 μm.
(I) Quantification of the mean intensity ± SEM of aPKC fluorescence in a 320 μm x 320 μm area.
(J-K) qPCR analysis of *Hes1* and *Gli1* mRNA in NIH-3T3 cells transiently transfected with either EF.CMV.RFP or EF.mHes1.CMV.GFP expression vectors and then cultured in the presence or absence of Shh for 12 hr. Plots represent mean *Gapdh*-normalized expression levels relative to EF.CMV.RFP controls ± SEM from 4-6 samples for each condition.

Figure S6. Inhibition of Notch signaling reduces Smo trafficking to primary cilia in a range of cell types (related to Figure 5).

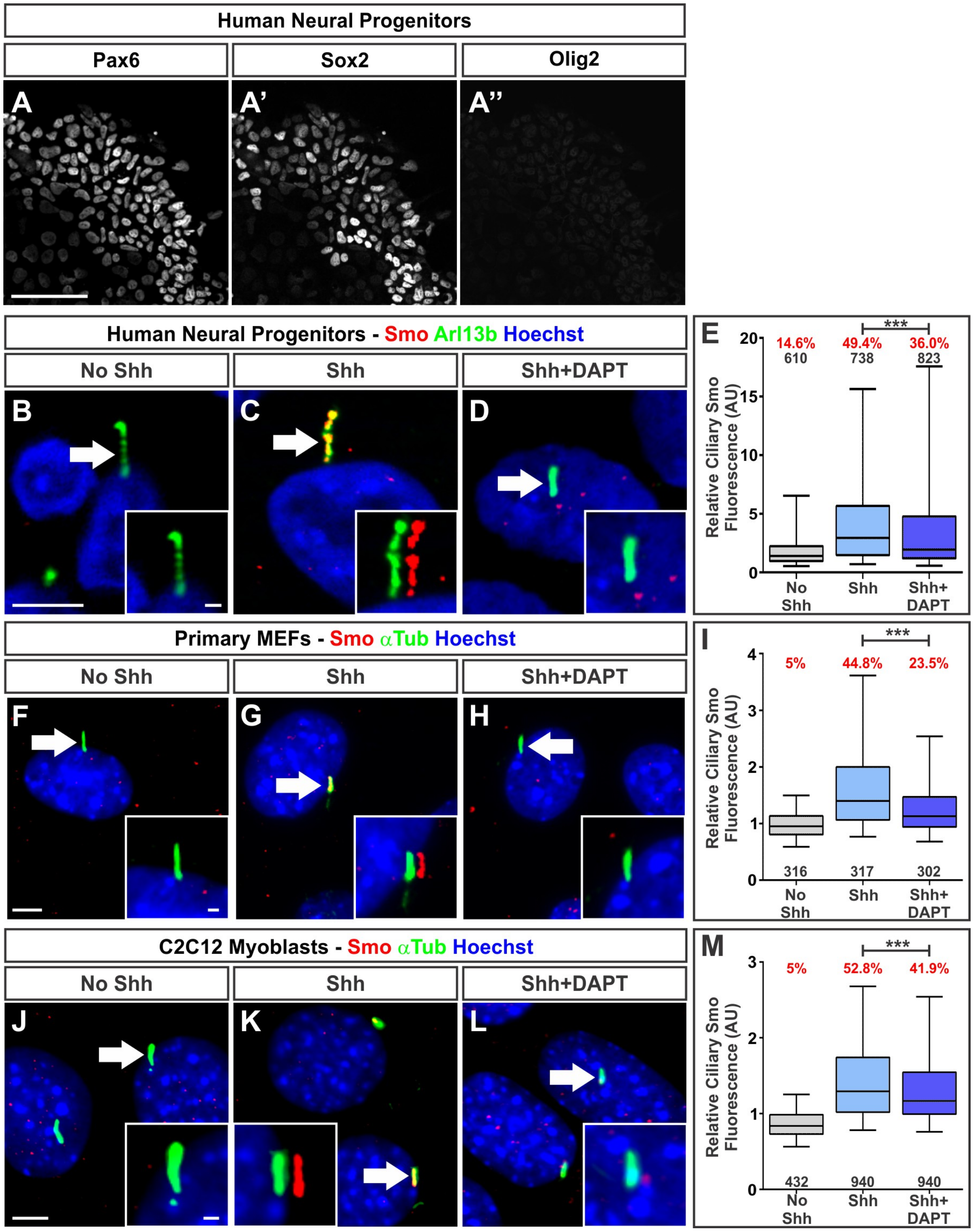

(A-A'') Immunofluorescence analysis of day 11 human spinal neural progenitors generated from the directed differentiation of H9 human embryonic stem cells. The majority of these cells express canonical neural progenitor markers such as SOX2 and PAX6, but not OLIG2 prior to the addition of Shh. Scale bars = 100 µm.

(B-M) Human neural progenitors (B-D), primary mouse embryonic fibroblasts (MEFs) (F-H), and mouse C2C12 skeletal myoblasts (J-L) exposed to 50 nM Shh with or without DAPT for 12 hr. Plated cells were immunostained for Smo (red), Arl13b or αTubulin (green, primary cilia), and Hoechst (blue). Arrows denote cilia shown in the insets, in which Smo and Arl13b/αTubulin channels are offset to better show colocalization. Low mag scale bars = 5 μm. High mag scale bars in insets = 1 μm.
(E, I, M) Box and whisker plots of Smo fluorescence in the cilia of various cell types. The box represents the 25th to 75th percentile, the line through the box represents the median, and the whiskers encompass the 5th to 95th percentile. The number of cilia analyzed is indicated in black and the percentage of Smo^{+} cilia indicated in red. ***$p < 0.001$.

Figure S7. Manipulating neural progenitor identities in a manner independent of Notch signaling does not alter Smo accumulation within primary cilia (related to Figure 6).

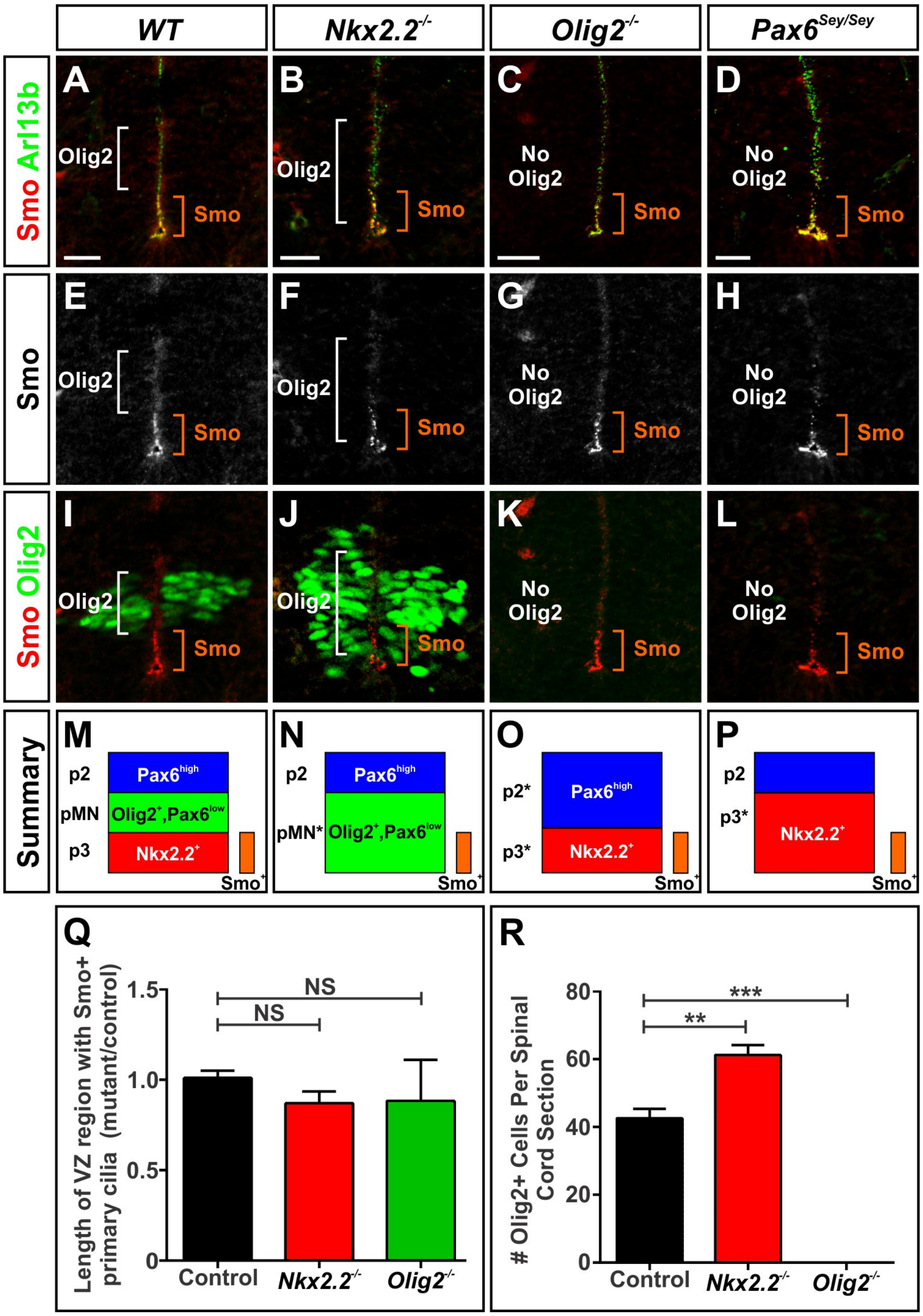

(A-L) Analysis of Smo^{+} primary cilia present on ventral spinal cord progenitors in E11.5 *WT* (A, E, I), *Nkx2.2*$^{-/-}$ (B, F, J), *Olig2*$^{-/-}$ (C, G, K), and *Pax6*$^{Sey/Sey}$ (D, H, L) mutants. Tissues were stained with antibodies against Smo, Arl13b, and Olig2. White brackets denote the limits of the Olig2^{+}/pMN progenitor domain and the orange brackets illustrate the dorsoventral extent of progenitor cells with Smo^{+} primary cilia. All scale bars = 20 μm.
(M-P) Summary of the mutant spinal cord data.

(Q) Quantification of the dorsoventral limits of Smo^{+} primary cilia in *WT*, *Nkx2.2*$^{-/-}$, and *Olig2*$^{-/-}$ mutants. Plots represent the mean length ± SEM of Smo^{+} cilia normalized against littermate controls. For this analysis, multiple sections were imaged from at least 3 embryos from each experimental group. NS, $p > 0.05$.

(R) Quantification of the number of $Olig2^{+}$ cells per spinal cord. Plots represent the mean ± SEM from multiple sections collected from 3-4 embryos from each group. $^{**}p < 0.01$ and $^{***}p < 0.001$.

Figure S8. DAPT Inhibition of Smo translocation to the primary cilia requires at least 12 hr exposure and is dependent on Ptch1 function (related to Figure 7).

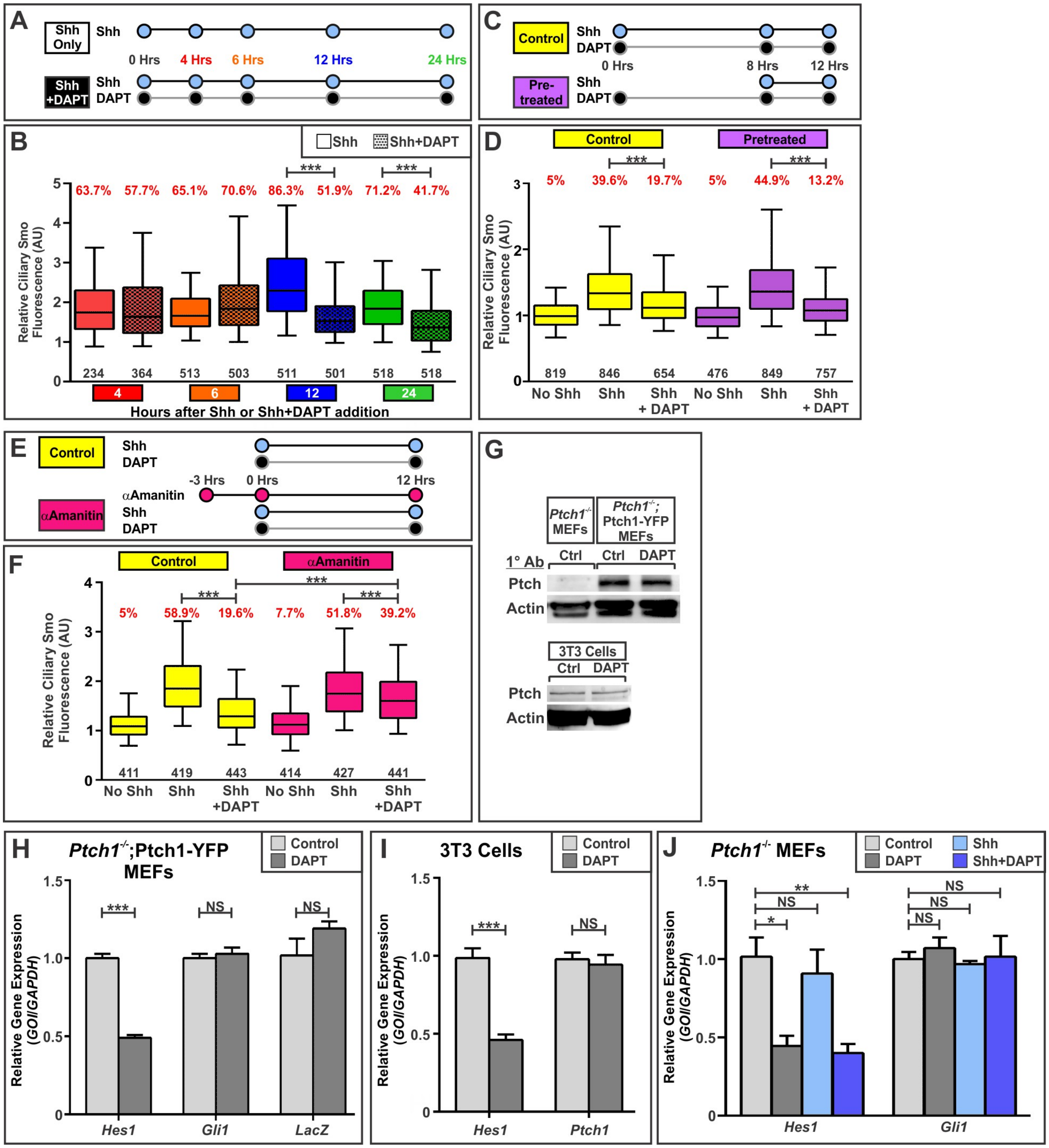

(A) Outline of the time course experiments in which NIH-3T3 cells were exposed to 50 nM Shh alone or in the presence of 18.75 µM DAPT for 4, 6, 12, and 24 hr.

(B) Box and whisker plots of Smo fluorescence in the cilia of cells treated with Shh ± DAPT for 4, 6, 12, and 24 hr. The box represents the 25th to 75th percentile, the line through the box represents the median, and the whiskers encompass the 5th to 95th percentile. The number of cilia analyzed is indicated in black below the box plots, and percentage of Smo^{+} cilia indicated in

red above the box plots. Reductions in Smo^+ primary cilia were first observed at 12 hr. ***p < 0.001.

(C) Outline of the pretreatment experiment, in which NIH-3T3 cells were exposed to either Shh+DAPT for 12 hr (Control) or exposed to DAPT alone for 8 hr and then Shh+DAPT for an additional 4 hr (Pretreatment).

(D) Box and whisker plots of ciliary Smo fluorescence under Control and DAPT Pretreated conditions. ***p < 0.001.

(E) Outline of the α-amanitin experiment. NIH-3T3 cells were exposed to the transcriptional inhibitor α-amanitin 3 hr prior to the addition of Shh with or without DAPT.

(F) Box and whisker plots of ciliary Smo fluorescence in the presence or absence of α-amanitin. ***p < 0.001.

(G) Immunoblotting for Ptch1 and Actin in cell lysates of *Ptch1*$^{-/-}$ MEFs, *Ptch1*$^{-/-}$*; Ptch1-YFP* MEFs, and NIH-3T3 cells treated with either DMSO (Control) or DAPT for 24 hr.

(H) qPCR analysis of *Ptch1*$^{-/-}$*;Ptch1-YFP* MEFs treated with either DMSO (Control) or DAPT for 12 hr. Plot shows mean *Gapdh*-normalized gene expression levels relative to the Control group ± SEM from 4 samples. NS, p > 0.05, ***p < 0.001, unpaired t-test.

(I) qPCR analysis of NIH-3T3 cells treated with DMSO (Control) or DAPT for 24 hr. Plot represents mean *Gapdh*-normalized gene expression levels relative to the control group ± SEM from 3 samples. NS, p > 0.05, *p < 0.05, **p < 0.01, ***p < 0.001.

(J) qPCR analysis of *Ptch1*$^{-/-}$ MEFs treated with DMSO (Control), DAPT, Shh, or Shh+DAPT for 24 hr. Plot represents mean *Gapdh*-normalized gene expression levels relative to the Control group ± SEM from 3 samples. NS, p > 0.05, *p < 0.05, **p < 0.01, ***p < 0.001.

SUPPLEMENTAL EXPERIMENTAL PROCEDURES

Antibodies and in situ hybridization probes

Primary antibodies used for immunohistochemistry were as follows: mouse anti-Arl13b (NeuroMab 75-287), 1:100; mouse anti-Ascl1 (Lo et al., 1991), 1:200; rabbit anti-BLBP (Chemicon/Millipore AB9558), 1:2,000; rabbit anti-β-Catenin (Sigma C2206), 1:1,000; guinea pig anti-Chx10 (Vsx2) (Thaler et al., 1999), 1:5,000; mouse anti-Cre (Covance MMS-106P), 1:2,000; rabbit anti-Dbx1 (Vue et al., 2007), 1:1,000; rabbit anti-Dbx2 (Abcam ab25554), 1:4,000; mouse anti-Foxa2 (Developmental Studies Hybridoma Bank 4C7), 1:200; rabbit anti-Foxa2 (Weinstein et al., 1994), 1:4,000; guinea pig anti-Foxp1 (Rousso et al., 2008), 1:16,000; goat anti-Gata3 (Santa Cruz Biotechnology sc-1236), 1:200; chicken anti-GFP (Aves Labs GFP-1020), 1:1,000; rabbit anti-GFP (Invitrogen A6455), 1:4000; sheep anti-GFP (AbD Serotec 4745-1051), 1:800; rabbit anti-Hb9 (Mnx1) (Arber et al., 1999), 1:8000; rabbit anti-Hes1 (Ito et al., 2000), 1:2,000; rabbit anti-Irx3 (Novitch et al., 2003), 1:8,000; goat anti-Islet1 (R&D Systems AF1837), 1:8,000; rabbit anti-Islet1/2 (Tsuchida et al., 1994), 1:20,000; rabbit anti-Lhx3 (Ericson et al., 1997), 1:4,000; rat anti-N-cadherin (Developmental Studies Hybridoma Bank MNCD2), 1:50; rabbit anti-N-cadherin (Abcam ab12221), 1:1,000; mouse anti-NeuN (Millipore MAB377B), 1:2,000; goat anti-Neurog2 (Santa Cruz Biotechnology sc-19233), 1:1,000; rabbit anti-Nf1a (Kang et al., 2012), 1:3,000; mouse anti-Nkx2.2 (Developmental Studies Hybridoma Bank 74.545), 1:25; rabbit anti-Nkx2.2 (Briscoe et al., 1999), 1:10,000; guinea pig anti-Nkx6.1 (Briscoe et al., 2000), 1:4,000; mouse anti-Nkx6.1 (Developmental Studies Hybridoma Bank F55A10), 1:25; rabbit anti-nNos (Immunostar 24287), 1:10,000; rabbit anti-Cleaved-Notch1 (Cell Signaling Technology 2421), 1:500; rabbit anti-Numb (Abcam ab14140), 1:4,000; rabbit anti- Olig2 (chick) (Novitch et al., 2001), 1:8,000; guinea pig anti-Olig2 (mouse) (Novitch et al., 2003), 1:20,000; rabbit anti-Olig2 (mouse) (Millipore AB9610), 1:5,000; rabbit anti-Par3 (Millipore 07-330), 1:100; mouse anti-Pax6 (Developmental Studies Hybridoma Bank), 1:100; rabbit anti-Pax6, (Covance), 1:4,000; rat anti-Pdgfrα (eBiosciences 14-1401), 1:1,000; rabbit anti-aPKC (PKCζ), (Santa Cruz Biotechnology SC-216), 1:1600; rabbit anti-Ptch1 (Rohatgi et al., 2007), 1:500; rat anti-Rbpj (Active Motif 61506), 1:100; mouse anti-Shh (Developmental Studies Hybridoma Bank 5E1), 1:100; rabbit anti-Smo (Abcam ab38686), 1:3,000; rabbit anti-Smo (Rohatgi et al., 2007), 1:500; goat anti-Sox2 (Santa Cruz Biotechnology sc-17320), 1:2,000; goat anti-Sox10 (R&D Systems AF2864), 1:300; mouse anti-αTubulin (Sigma T6793), 1:1,000; rabbit anti-βIII-Tubulin (TUJ1) (Covance MRB-435P), 1:5,000. Alexa^{488}-, FITC-, Cy3-, Cy5-, and Dylight^{649}-conjugated secondary antibodies were obtained from Jackson Immunoresearch Laboratories, Inc. (West Grove, PA). RNA probes were generated by in vitro transcription of PCR products amplified from mouse spinal cord cDNA. Primers against the following genes were designed using the program Primer3 (http://bioinfo.ut.ee/primer3/). *Hes5*, forward 5'-GGATGAGCTCGTTCCTCTGG-3' and reverse 5'-GAGATTAACCCTCACTAAAGGGAGCAGGCTGAGTGCTTTCCTA-3' and *Neurog3*, forward 5'-AGGCTTCTCATCGGTACCCT-3' and reverse 5'-GAGATTAACCCTCACTAAAGGGCATAGGCTAGGGCTTTCCGG-3'. The underlined text denotes a T3 polymerase binding site incorporated into the reverse primer. Probes for *Fgfr3* (Gaber et al., 2013), *Slit1* (Hochstim et al., 2008), *Gli1* and *Ptch1* (Ribes et al., 2010) were used as previously described. All probes were generated using a Digoxigenin (DIG) RNA Labeling Kit (Roche) and visualized using a combination of Anti-DIG-alkaline phosphatase (AP) Fab fragments (Roche) and NBT/BCIP (Roche).

Chick neural explant culture

Neural tissue was isolated from chick embryos (HH stage 10) and cultured as previously described (Yamada et al., 1993; Dessaud et al., 2007). Two types of explants were prepared: intermediate spinal cord explants (i) and ventral neural plate plus floor plate explants (vf). Shh

was generated as previously described (Ericson et al., 1996) and the concentration of each new batch determined by comparison with previous batches. NKX2.2 and OLIG2 quantifications consist of data collected from 7-10 explants per condition. Plotted data represents the mean ± SEM.

Quantitative PCR

qPCR was carried out as previously described (Rousso et al., 2012). Briefly, total RNA was extracted using the RNeasy Mini Kit (Qiagen). For each sample, ~500-1000 ng of total RNA was used to synthesize cDNA by reverse transcription using the SuperScript III First-Strand Synthesis System (Invitrogen). In each qPCR reaction, cDNA was combined with LightCycler 480 SYBR Green I Master Mix (Roche) and the following exon-spanning primer pairs: mouse *Gli1*, forward 5'-CACCGTGGGAGTAAACAGGCCTTCC-3' and reverse 5'-CCAGAGCGTTACACACCTGCCCTTC-3' (Regl et al., 2002); mouse *Gli1*, forward 5'-ATCTCTCTTTCCTCCTCCTCC-3' and reverse 5'-CGAGGCTGGCATCAGAA-3' (Lelievre et al., 2008); mouse *Patched1*, forward 5'-CTGCCTGTCCTCTTATCCTTC-3' and reverse 5'-AGACCCATTGTTCGTGTGAC-3'; mouse *Sox2*, forward 5'-CACATGGCCCAGCACTAC-3' and reverse 5'-CCCTCCCAATTCCCTTGTATC-3'; mouse *Gapdh*, forward 5′–GGCCTTCCGTGTTCCTAC–3′ and reverse 5′–TGTCATCATACTTGGCAGGTT–3' (Lelievre et al., 2008); mouse *Hes1*, forward 5'-GGCGAAGGGCAAGAATAAATG-3' and reverse 5'-GTGCTTCACAGTCATTTCCAG-3'. The 18 to 20-mer primers were either chosen from papers as cited or designed using the IDT Primer Quest Program (http://www.idtdna.com/primerquest/Home/Index). All primer pairs were experimentally validated using E10.5 whole spinal cord cDNA. In this validation process each pair was shown to have amplification efficiency between 1.8-2.2 and possess a single gene-specific product using melting curve analysis. All samples were run using a Roche LightCycler 480 real-time PCR system in duplicates or triplicates, and relative mRNA expression levels determined by normalizing the crossing points of each gene of interest to *Gapdh*. Unless otherwise indicated relative gene expression profiles were plotted by comparison to the average value of control samples, set to 1.0.

Cell Culture

Reagents: Shh (Akron Biotech) was reconstituted in 0.1% BSA, DAPT and Purmorphamine (Calbiochem) in DMSO, and SAG (Calbiochem) in water. To account for these different vehicles, all control samples were treated with equivalent volumes of DMSO and BSA to experimental samples. The concentrations of Shh, Pur, SAG, and DAPT used were empirically determined by exposing fibroblast cells to a range of doses and assessing Shh and Notch signaling activity using qPCR and luciferase assays. Across batches, Shh (50 nM), Pur (5 μM), and SAG (1 μM) consistently activated Shh signaling. However, there were some potency differences between batches of DAPT (5-25 μM). To account for these differences, we optimized the concentration of each lot of DAPT using qPCR measurement of *Hes1* mRNA to readout Notch signaling activity. In each experiment, the amount of DAPT used was found to reduce *Hes1* expression by at least 50%.

NIH-3T3 cells and primary MEFs: NIH-3T3 cells were cultured in high glucose (4.5 g/L glucose) Dulbecco's modified Eagle's medium (DMEM) with 110 mg/L sodium pyruvate (Gibco), 10% bovine calf serum (BCS, Gibco), 1x glutamax (Gibco), 1% Penicillin:Streptomycin (Gibco), and 0.2% Primocin (InvivoGen). Shh-LIGHT2 cells were cultured in high glucose (4.5 g/L glucose) DMEM with 110 mg/L sodium pyruvate (Gibco), 10% BCS (Gibco), 0.15 mg/ml zeocin (Invitrogen), and 0.4 mg/ml geneticin (Gibco), as previously described (Taipale et al., 2000). Wild-type, *Ptch1*$^{-/-}$*, and Ptch1*$^{-/-}$;Ptch1-YFP mouse embryo fibroblasts were cultured as previously described (Rohatgi et al., 2007). For Shh induction experiments, cells were plated onto 8 mm coverslips in 24-well plates (40,000 cells/well) in regular (10% BCS) growth media.

Upon reaching 80-100% confluency, cells were moved into low serum media (0.5% BCS) supplemented with one or more of the following: Shh ligand (50 nM, Akron Biotech), Purmorphamine (5 μM, Calbiochem), SAG (1 μM, Calbiochem), DAPT (18.75 μM, Calbiochem), SAHM (20 μM, Calbiochem), and JLK6 (20 μM, Calbiochem).
C2C12 myoblasts: C2C12 cells were plated directly onto 8 mm coverslips in a 24-well plate (40,000 cells/well) in DMEM with L-glutamine (Gibco) supplemented with 10% FBS. Upon reaching 80-100% confluency, the cells were switched to a low serum media (0.5% FBS) and treated with Shh ± DAPT for 12 hr.
Human neural progenitors: Primitive human neuroepithelial progenitors were generated from embryonic stem cells as previously described (Hu and Zhang, 2009). After 10 days of culture in vitro, neural rosettes were manually picked and plated onto poly-ornithine/laminin coated coverslips in DMEM/F12 (Hyclone), 1x N2 (Life Technologies), 0.1mM NEAA (Life Technologies), 1 mg/mL heparin (Sigma), and 10% FBS (Hyclone), and allowed to attach for 24 hr. The following day, FBS was removed and the neural rosettes were exposed to Shh ± DAPT for 12 hr.

Transient transfection of NIH-3T3 cells
NIH-3T3 cells were transiently transfected with either pCIG, pCIG-NICD, EF.CMV.RFP (Addgene plasmid # 17619), or EF.mHes1.CMV.GFP (Addgene plasmid # 17622) constructs (Megason and McMahon, 2002; Yu et al., 2003; Yu et al., 2006) using the transfection reagent FuGENE6 (Roche) at a DNA:FuGENE6 ratio of 1:3.

Luciferase assays
In chick explants: Gli activity was measured in chick neural explants as previously described (Dessaud et al., 2007). Briefly, chick embryos (HH stage 10) were coelectroporated with a Gli-binding site firefly luciferase reporter and a cytomegalovirus promoter::renilla luciferase plasmid (Promega) to normalize for transfection efficiency, returned to the incubator for 2 hr, and then collected for explant culture. Upon isolation, neural explants were exposed to Shh ± DAPT for 12 or 24 hr. Tissue was then homogenized on ice in Passive Lysis Buffer and processed using a Dual-Luciferase Reporter Assay System (Promega). Each data point represents the mean ± SEM from 7-10 explants.
In cells: All luciferase assays were conducted using Shh-LIGHT2 cells, NIH-3T3 cells that have been stably cotransfected with Gli-binding site::firefly luciferase and herpes simplex virus thymidine kinase (HSV TK) promoter::renilla luciferase plasmids (Taipale et al., 2000). Shh-LIGHT2 cells were cultured in 96-well plates (30,000 cells/well) in regular serum media (10% BCS). Upon reaching 80-100% confluency, the cells were moved into a low serum media (0.5% BCS) containing Shh ligand (0-50 nM, Akron Biotech) ± DAPT (0-50 μM, Calbiochem). After 24 hr, cells were rinsed in PBS and then lysed in Passive Lysis Buffer (100 μl/well, Promega Dual-Luciferase Reporter Assay System). Luciferase activities were measured in a Tecan M1000 microplate reader equipped with an automatic injector. For each sample, Gli-dependent firefly luciferase activity was normalized to HSV TK::renilla luciferase activity and the resulting ratio reported in relative luciferase units (RLU). All luciferase conditions were run as duplicate or triplicate samples. Each data point represents the mean ± SEM.

Tissue image analysis
Fluorescence and DIC images were collected using either a Zeiss LSM5 Exciter or LSM780 confocal imaging system or a Zeiss AxioImager M2 fluorescence microscope equipped with Apotome attachment and motorized stage. Images were collected and processed using LSM Exciter, Zeiss AxioVision, Zeiss Efficient Navigation (ZEN), and Adobe Photoshop software. Fluorescence intensity quantifications and cell number counts were performed using the NIH

developed image processing program ImageJ with an Image-based Tool for Counting Nuclei (ITCN) plugin. Composite images were assembled using CorelDRAW X7 software.

Analysis of Smo and Ptch1 presence in primary cilia

Fixed cells were imaged on an inverted Zeiss LSM 780 laser scanning confocal microscope or an upright Zeiss AxioImager M2 fluorescence microscope equipped with Apotome attachment and motorized stage. Images were taken with 20x, 40x oil, and 63x oil immersion objectives. For each experiment, coverslips from each condition were processed side by side to ensure that the cells were fixed and stained for the same duration of time. To ensure uniformity in imaging, the gain, offset, and laser power settings on the microscope were held constant for Smo and Ptch1. Quantification of relative ciliary Smo and Ptch1 fluorescence levels were performed as previously described (Mukhopadhyay et al., 2013; Nachtergaele et al., 2013), with minor modifications. Briefly, using the program ImageJ, an outline was first drawn around each cilium (labeled by α-acetylated tubulin or Arl13b staining), and the corresponding intensity of Smo or Ptch1 fluorescence within and adjacent to the cilium measured. From these measurements, a ratio of the intensity was then calculated for each cilium (ratio = intensity of fluorescence within the cilium ÷ fluorescence adjacent to the cilium). When this ratio (i.e. relative ciliary Smo or Ptch1 fluorescence) is close to 1, the intensity of Smo or Ptch1 fluorescence within the cilium is not above background levels. The relative fluorescence values within a primary cilium that visually appeared to be Smo or Ptch1 positive varied widely based on cell type. In NIH-3T3 cells, the relative Smo or Ptch1 fluorescence within a positive primary cilium was at least 1.3-1.5. In human neural progenitors, the relative Smo fluorescence within a positive primary cilium was at least 3. Smo and Ptch1 were measured in approximately 100-800 primary cilia per each experimental group. These 100-800 relative ciliary fluorescence values were then represented in a box and whisker graph. In each graph, the upper and lower limits of the box represent the 25th to 75th percentiles, the line in the center is the median, and the whiskers encompass the 5th to the 95th percentiles. In each graph, the black numbers present above or below the box plots are the number of primary cilia analyzed for each group and the red numbers are the percentage of Smo or Ptch1 positive cilia. Although all statistical evaluations were done using the relative ciliary fluorescence values, Smo or Ptch1 positive cilia percentages were included on each plot to distill the data into binary “positive” or “negative” cilia values. To determine if a primary cilium was Smo or Ptch1 positive, we established a “cut-off” ciliary fluorescence value unique to each experiment, as there was frequently variance in background staining values. We then grouped all cilia above the cut-off as positive and those below as negative. In most experiments, the cut-off was determined to be the value at which there were 5% Smo^+ or $Ptch1^+$ primary cilia in the negative control group (i.e. the group that possessed the fewest Smo or Ptch1-positive cilia). The negative control for Smo was the No Shh group and the negative control for Ptch1 was the Shh-only treated group. In some experiments, no true negative control group was present and in these cases the cut-off value was set to 1.5 for NIH-3T3 cells and 3.0 for human neural progenitors, based on the background staining values in surrounding regions away from the cilia.

Analysis of primary cilia lengths

Primary cilia of fixed cells were labeled using α-acetylated tubulin and Arl13b antibodies, and imaged on an inverted Zeiss LSM 780 laser scanning confocal microscope equipped with either a 63x or 100x oil immersion objective. To ensure the full length of each cilium was accurately measured, thin z-stacks were acquired and 3D surface reconstructions of the primary cilia generated using ZEN software (Carl Zeiss, Germany). All experimental cilia measurements were normalized against control cilia measurements. The values reported thus represent the mean changes in the percentage of cilia length ± SEM. For each condition, the lengths of over 100 cilia were individually analyzed. The reduction in primary cilia length observed with DAPT

addition and extension with NICD transfection was observed with both α-acetylated tubulin and Arl13b labeled cilia. Data was analyzed using an unpaired, two-tailed t-test. Significance was assumed when $p < 0.05$.

Immunoblotting
Cell cultures were scaled up to 100 mm plates and grown as described above. Cells were manually scraped, rinsed once in PBS, lysed in a modified RIPA buffer (1% NP40, 1% sodium deoxycholate, 0.3% SDS, 150 mM NaCl, 1 mM EDTA, 50 mM Tris pH6.8, 1 mM PMSF, 1x complete protease cocktail (Roche)) for 2 hr on ice with vortexing every 10 min, and clarified by centrifugation for 30 min at 14,000 RPM at 4ºC. Protein lysates were resolved on an 8-10% polyacrylamide gel and processed for immunoblotting with the following antibodies: Rabbit anti-Notch1 (Val1744, Cell Signaling Technologies), 1:1000; rabbit anti-Ptch1 (Rohatgi Lab), 1:1000; mouse anti-actin (Millipore), 1:1000, followed by HRP-conjugated anti-mouse and anti-rabbit secondary antibodies (Jackson Immunoresearch), 1:50,000. Membranes were processed using ECL 2 Western Blotting Substrates (Thermo Scientific) and bands detected using a Typhoon FLA 7000 imaging system (GE Healthcare Life Sciences).

SUPPLEMENTAL REFERENCES

Arber, S., Han, B., Mendelsohn, M., Smith, M., Jessell, T.M., and Sockanathan, S. (1999). Requirement for the homeobox gene Hb9 in the consolidation of motor neuron identity. Neuron *23*, 659-674.

Briscoe, J., Sussel, L., Serup, P., Hartigan-O'Connor, D., Jessell, T.M., Rubenstein, J.L., and Ericson, J. (1999). Homeobox gene Nkx2.2 and specification of neuronal identity by graded Sonic hedgehog signalling. Nature *398*, 622-627.

Ericson, J., Rashbass, P., Schedl, A., Brenner-Morton, S., Kawakami, A., van Heyningen, V., Jessell, T.M., and Briscoe, J. (1997). Pax6 controls progenitor cell identity and neuronal fate in response to graded Shh signaling. Cell *90*, 169-180.

Ito, T., Udaka, N., Yazawa, T., Okudela, K., Hayashi, H., Sudo, T., Guillemot, F., Kageyama, R., and Kitamura, H. (2000). Basic helix-loop-helix transcription factors regulate the neuroendocrine differentiation of fetal mouse pulmonary epithelium. Development *127*, 3913-3921.

Kang, P., Lee, H.K., Glasgow, S.M., Finley, M., Donti, T., Gaber, Z.B., Graham, B.H., Foster, A.E., Novitch, B.G., Gronostajski, R.M., *et al.* (2012). Sox9 and NFIA coordinate a transcriptional regulatory cascade during the initiation of gliogenesis. Neuron *74*, 79-94.

Lelievre, V., Seksenyan, A., Nobuta, H., Yong, W.H., Chhith, S., Niewiadomski, P., Cohen, J.R., Dong, H., Flores, A., Liau, L.M., *et al.* (2008). Disruption of the PACAP gene promotes medulloblastoma in ptc1 mutant mice. Dev Biol *313*, 359-370.

Lo, L.C., Johnson, J.E., Wuenschell, C.W., Saito, T., and Anderson, D.J. (1991). Mammalian achaete-scute homolog 1 is transiently expressed by spatially restricted subsets of early neuroepithelial and neural crest cells. Genes Dev *5*, 1524-1537.

Megason, S.G., and McMahon, A.P. (2002). A mitogen gradient of dorsal midline Wnts organizes growth in the CNS. Development *129*, 2087-2098.

Mukhopadhyay, S., Wen, X., Ratti, N., Loktev, A., Rangell, L., Scales, S.J., and Jackson, P.K. (2013). The ciliary G-protein-coupled receptor Gpr161 negatively regulates the Sonic hedgehog pathway via cAMP signaling. Cell *152*, 210-223.

Nachtergaele, S., Whalen, D.M., Mydock, L.K., Zhao, Z., Malinauskas, T., Krishnan, K., Ingham, P.W., Covey, D.F., Siebold, C., and Rohatgi, R. (2013). Structure and function of the Smoothened extracellular domain in vertebrate Hedgehog signaling. Elife *2*, e01340.

Novitch, B.G., Wichterle, H., Jessell, T.M., and Sockanathan, S. (2003). A requirement for retinoic acid-mediated transcriptional activation in ventral neural patterning and motor neuron specification. Neuron *40*, 81-95.

Regl, G., Neill, G.W., Eichberger, T., Kasper, M., Ikram, M.S., Koller, J., Hintner, H., Quinn, A.G., Frischauf, A.M., and Aberger, F. (2002). Human GLI2 and GLI1 are part of a positive feedback mechanism in Basal Cell Carcinoma. Oncogene *21*, 5529-5539.

Ribes, V., Balaskas, N., Sasai, N., Cruz, C., Dessaud, E., Cayuso, J., Tozer, S., Yang, L.L., Novitch, B., Marti, E., *et al.* (2010). Distinct Sonic Hedgehog signaling dynamics specify floor plate and ventral neuronal progenitors in the vertebrate neural tube. Genes Dev *24*, 1186-1200.

Thaler, J., Harrison, K., Sharma, K., Lettieri, K., Kehrl, J., and Pfaff, S.L. (1999). Active suppression of interneuron programs within developing motor neurons revealed by analysis of homeodomain factor HB9. Neuron *23*, 675-687.

Vue, T.Y., Aaker, J., Taniguchi, A., Kazemzadeh, C., Skidmore, J.M., Martin, D.M., Martin, J.F., Treier, M., and Nakagawa, Y. (2007). Characterization of progenitor domains in the developing mouse thalamus. J Comp Neurol *505*, 73-91.

Weinstein, D.C., Ruiz i Altaba, A., Chen, W.S., Hoodless, P., Prezioso, V.R., Jessell, T.M., and Darnell, J.E., Jr. (1994). The winged-helix transcription factor HNF-3 beta is required for notochord development in the mouse embryo. Cell *78*, 575-588.

Yamada, T., Pfaff, S.L., Edlund, T., and Jessell, T.M. (1993). Control of cell pattern in the neural tube: motor neuron induction by diffusible factors from notochord and floor plate. Cell *73*, 673-686.

Yu, X., Alder, J.K., Chun, J.H., Friedman, A.D., Heimfeld, S., Cheng, L., and Civin, C.I. (2006). HES1 inhibits cycling of hematopoietic progenitor cells via DNA binding. Stem Cells *24*, 876-888.

Yu, X., Zhan, X., D'Costa, J., Tanavde, V.M., Ye, Z., Peng, T., Malehorn, M.T., Yang, X., Civin, C.I., and Cheng, L. (2003). Lentiviral vectors with two independent internal promoters transfer high-level expression of multiple transgenes to human hematopoietic stem-progenitor cells. Mol Ther *7*, 827-838.
